# Pseudogenization of the rhizobium-responsive *EXOPOLYSACCHARIDE RECEPTOR* in *Parasponia* is a rare event in nodulating plants

**DOI:** 10.1186/s12870-022-03606-9

**Published:** 2022-04-30

**Authors:** Simon Dupin, Joël Klein, Luuk Rutten, Rik Huisman, Rene Geurts

**Affiliations:** 1 Laboratory of Molecular Biology, Department of Plant Science, Wageningen University, Droevendaalsesteeg 1, 6703PB Wageningen, The Netherlands; 2 Department of Ecological Science. Faculty of Earth and Life Sciences, Vrije Universiteit, De Boelelaan 1085, 1081HV Amsterdam, The Netherlands

**Keywords:** EXOPOLYSACCHARIDE RECEPTOR, EPR3, *Parasponia*, *Trema*, Nodulation

## Abstract

**Background:**

Nodule symbiosis with diazotrophic *Frankia* or rhizobium occurs in plant species belonging to ten taxonomic lineages within the related orders Fabales, Fagales, Cucurbitales, and Rosales. Phylogenomic studies indicate that this nitrogen-fixing nodulation trait has a single evolutionary origin. In legume model plants, the molecular interaction between plant and rhizobium microsymbiont is mapped to a significant degree. A specific LysM-type receptor kinase, LjEPR3 in *Lotus japonicus* and MtLYK10 in *Medicago truncatula*, was found to act in a secondary identity-based mechanism, controlling intracellular rhizobium infection. Furthermore, LjEPR3 showed to bind surface exopolysaccharides of *Mesorhizobium loti*, the diazotrophic microsymbiont of *L. japonicus*. *EPR3* orthologous genes are not unique to legumes. Surprisingly, however, its ortholog *EXOPOLYSACCHARIDE RECEPTOR* (*EPR*) is pseudogenized in *Parasponia,* the only lineage of non-legume plants that nodulate also with rhizobium.

**Results:**

Analysis of genome sequences showed that *EPR3* orthologous genes are highly conserved in nodulating plants. We identified a conserved retrotransposon insertion in the *EPR* promoter region in three *Parasponia* species, which associates with defected transcriptional regulation of this gene. Subsequently, we studied the *EPR* gene of two *Trema* species as they represent the sister genus of *Parasponia* for which it is assumed it lost the nitrogen-fixing nodulation trait. Both *Trema* species possess apparently functional *EPR* genes that have a nodulation-specific expression profile when introduced into a *Parasponia* background. This indicates the *EPR* gene functioned in nodulation in the *Parasponia-Trema* ancestor.

**Conclusion:**

We conclude that nodule-specific expression of *EPR3* orthologous genes is shared between the legume and *Parasponia-Trema* lineage, suggesting an ancestral function in the nitrogen-fixing nodulation trait. Pseudogenization of *EPR* in *Parasponia* is an exceptional case in nodulating plants. We speculate that this may have been instrumental to the microsymbiont switch -from *Frankia* to rhizobium- that has occurred in the *Parasponia* lineage and the evolution of a novel crack entry infection mechanism.

**Supplementary Information:**

The online version contains supplementary material available at 10.1186/s12870-022-03606-9.

## Introduction

The ability to engage in a nodule endosymbiosis with diazotrophic *Frankia* or rhizobium soil bacteria is a trait present in ten plant lineages within the taxonomic orders Fabales, Fagales, Cucurbitales and Rosales [1]. These four orders are commonly known as the nitrogen-fixing clade (NFC), but also represent multiple lineages of non-nodulating plants [2]. Recent phylogenomic studies indicated that the absent-present pattern of nitrogen-fixing root nodules in the NFC is the result of a single evolutionary gain of the nodulation trait, followed by multiple parallel losses [3–5]. In such a scenario, switches of microbial partners may have occurred.

A key feature of the nodulation trait is the potential to form a partnership with diazotrophic bacteria, in which the bacteria are carried to a newly formed root organ -the nodule- to establish an endosymbiosis. This bacterial infection is typically supported by plant-derived tubular structures, called infection threads, that transport bacteria to dividing root cortical cells that form the nodule primordium. Finally, infection threads penetrate into nodule cells allowing bacteria to fill most of the cytoplasmic space. The plant host provides carbohydrates to symbiotic bacteria that then fix di-nitrogen gas (N_2_) to ammonia, which is metabolized by the plant.

Nodule formation relies on a complex cross-talk between plant and microbial partners. This cross-talk can vary in its specificity depending on which partners are involved. For example, in *Lotus japonicus* – an important legume model species—infection thread progression and cell infection are granted by recognition of compatible rhizobia surface exopolysaccharides (EPS) by the host’s trans-membrane lysin motif (LysM) receptor kinase EXOPOLYSACHARIDE RECEPTOR 3 (LjEPR3) [6, 7]. LjEPR3 harbours a singular configuration of its three LysM domains (LysM1-LysM2-LysM3) due to the atypical topology of LysM1 [6, 8]. As a result, the extracellular domain of LjEPR3 is specific to EPS and does not bind to fungal and rhizobia chitooligosaccharide and lipo-chitooligosaccharide signal molecules (COs and LCOs) [8]. Thus, LjEPR3 works as a secondary identity-based mechanism in the establishment of nitrogen-fixing nodule symbiosis between *L. japonicus* and its microsymbiont *Mesorhizobium loti.*

Studies on EPR3-type LysM receptors in species other than *L. japonicus* are limited. In *Medicago truncatula*, the *LjEPR3* ortholog *MtLYK10* is crucial for the progression of the infection thread to the nodule primordia. But recognition of succinoglycan -the surface EPS of the *M. truncatula* compatible microsymbiont *Sinorhizobium meliloti*- was not found [9]. EPR3-type receptors do occur also in non-nodulating plant species [8, 9], however, surprisingly is lost in the nodulating Cannabaceae species *Parasponia *[4].

*Parasponia* is the only taxonomic lineage outside the legume clade that can establish nitrogen-fixing root nodules with rhizobium. *Parasponia* represents five nodulating tropical tree species growing on volcanic islands of Indonesia and Papua New Guinea [10, 11]. *Parasponia* is closely related to the genus *Trema,* which includes 18 species that do not nodulate [4, 12]. Comparative analysis of *Trema* and *Parasponia* species showed that loss of the EPR3-type receptor *EPR* is specific to *Parasponia* species [4]. Here we aim to characterise the evolutionary trajectory of *EPR* in the *Parasponia—Trema* lineage. Specifically, we ask whether *EPR* may have functioned in nodulation in an ancestral *Parasponia—Trema* species, and how common loss of *EPR3* orthologous genes is in nodulating species. Furthermore, we discuss whether the loss of *EPR* in *Parasponia* was instrumental to the microsymbiont switch that occurred in this lineage.

## Results

### A retrotransposon insertion caused *epr* pseudogenization in *Parasponia* species

*Parasponia* represents five species, three for which genome sequence data have been generated; *P. andersonii, P. rigida*, and *P. rugosa*, respectively [4]. Earlier analysis revealed that these *Parasponia* species, as well as close relatives of the genus *Trema,* possess a single *LjEPR3/MtLYK10* orthologous gene named *EXOPOLYSACCHARIDE RECEPTOR* (*EPR*). *P. andersonii* and *P. rigida EPR* accumulated different mutations in the first exon causing a disruption of the predicted open reading frame (ORF), whereas *P. rugosa EPR* experienced a large deletion affecting exons 1 to 5 (Table 1). As these mutations in *EPR* are not shared between the three *Parasponia* species, they must have occurred in parallel. This may suggest that the loss of *EPR* in *Parasponia* is the result of genetic erosion rather than specific selection. Alternatively, a shared, but yet unknown mutation occurred in the non-coding region of the gene affecting its functioning.

**Table 1:** Independent mutations in the presumed coding region of the *epr *pseudogene of three *Parasponia* species

**species**	**gene name**	**mutation in cds**	**encoded protein**	**GeneBank**
*P. andersonii*	*Panepr*	TAA^508−510^ stop codon	169 AA	KY786136.1
*P. rigida*	*Priepr*	A^214^ insertion	72 AA	KY786146.1
*P. rugosa*	*Pruepr*	362-1217 bp inframe deletion	326 AA	KY786178.1
*T. orientalis*	*TorEPR*^a^	no mutations	610 AA	JXTC01000021.1
*T. levigata*	*TleEPR*	no mutations	610 AA	KY786208.1

^a^Note: *TorEPR* is named *TorLYK2* in GeneBank

**Fig. 1:**
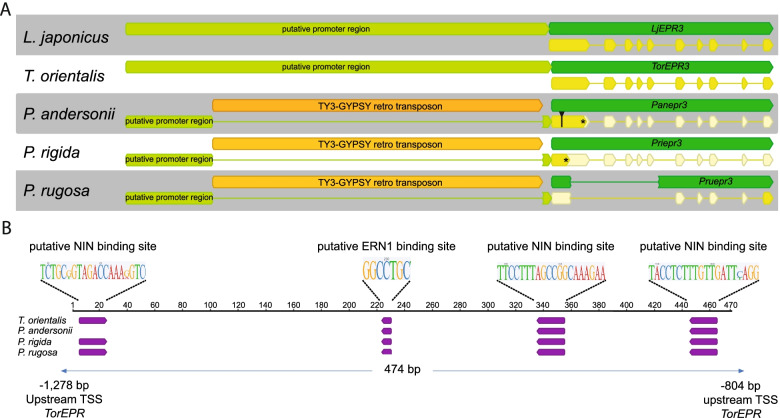
Gene structure of *Parasponia* and *Trema* *EPR*. **A** Gene structure of *L. japonicus LjEPR3, T. orientalis EPR,* and the *epr* pseudogene in *P. andersonii, P. rigida,* and *P. rugosa.* Note retrotransposon insertion (annotated in orange) in the putative promoter region of *Parasponia* species. Promoter (light green), gene (green), CDS = coding DNA sequence (yellow). **B** LOGOs of putative NIN and ERN1 binding motifs in the promoter region of *T. orientalis EPR* and the *epr* pseudogenes of *P. andersonii, P. rigida,* and *P. rugosa.* TSS: translational start site

To find evidence for this latter scenario, we investigated the putative promoter region of *EPR* in *Parasponia* and *Trema* species. In *L. japonicus* the functional promoter region of *LjEPR3* is relatively short, spanning only 329 bp upstream of the translational start codon [7]. We analysed the *EPR* promoter region in three *Parasponia* and two *Trema* species. The alignment of these promoters revealed a large 5,7 kb insertion in all three *Parasponia* species, just 154 bp upstream of the predicted translational start codon (Fig. 1A; Supplemental data file 1). Homology searches using BLAST revealed that this insertion represents a unique TY3-GYPSY-type retrotransposon element, which occurs only as a single copy in the genomes of the three *Parasponia* species, whereas it is absent in *Trema*. We compared the expression of the *P. andersonii epr* pseudogene to close homologs of the LysM-type receptor kinase (*LYK*) family [13]. This uncovered that in none of the samples, *Panepr* expression was observed, including roots and nodules at different stages of development (Figure S1). This supports that the retrotransposon insertion in the putative regulatory region of *EPR* could have been instrumental for the pseudogenization of this gene in the *Parasponia* lineage.

### *Trema EPR* is expressed in rhizobium-induced nodules

In *L. japonicus*, the *LjEPR3* promoter possesses putative binding sites for the nodulation-specific transcription factors NIN and ERN1 [7]. We analysed the putative promoter region of *Trema* and *Parasponia EPR* using MEME combined with manual curation [14, 15]. This predicted the occurrence of conserved putative transcription factor binding sites for ERN1 (1x) and NIN (3x), both in *Trema* and *Parasponia EPR* promoters in a confined ~ 500 bp region (Fig. 1B). This may suggest that transcriptional regulation of *EPR3* ortholog genes is conserved in legumes and non-legumes. As the putative NIN and ERN1 binding sites are present also in the *T. orientalis EPR* promoter, we questioned whether *Trema EPR* still possesses a nodule-enhanced expression profile, despite the loss of the nodulation trait.

**Fig. 2:**
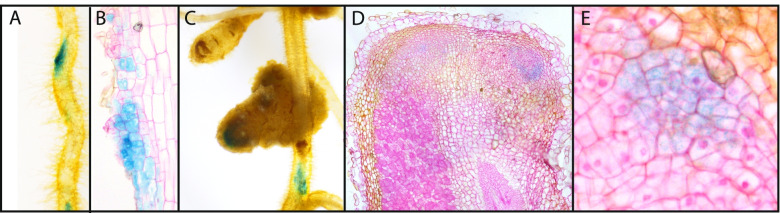
Temporal-spatial expression of *Trema orientalis EPR* promoter-GUS reporter constructs in *Parasponia andersonii. ***A** GUS staining in young a nodule primordium. **B** Longitudinal section of a rhizobium-induced young nodule primordium formed in the outermost cell layers of the root. **C** GUS staining in a narrow zone in the apical region of a nodule. **D** Longitudinal section of a mature nodule with pTorEPR:*GUS* activity in cells just below the meristem. **E** Enlargement o*f *pTorEPR:*GUS* expressing cells in a nodule. Note absence of intracellular infection in cells showing *TorEPR* promoter activity

To find support for the functioning of *EPR* in nodulation in a *Trema-Parasponia* ancestor, we generated transgenic *P. andersonii* lines carrying a *TorEPR* promoter GUS reporter construct (pTorEPR:*GUS*). As a putative promoter, a fragment of 1,730 bp upstream of the translational start codon was used, which includes the putative NIN and ERN1 binding sites. Two independent transgenic lines were studied. GUS staining of root tissue did not reveal any blue staining. Subsequently, plantlets (2 × *n* = 10) were inoculated with the compatible strain *Bradyrhizobium elkanii* WUR3 [16] and studied 4 and 8 weeks post-inoculation. *TorEPR* protomer GUS activity was observed in rhizobium-induced cell divisions (Fig. 2A,B), which in *P. andersonii* occur in the root epidermis and outer cortical cell layers [17]. In mature nodules, pTorEPR:*GUS* induced blue staining is confined to the meristematic zones in the apex of the nodule (Fig. 2C-E). In both cases, the *GUS* expressing cells were yet to be infected by rhizobium.

**Fig. 3:**
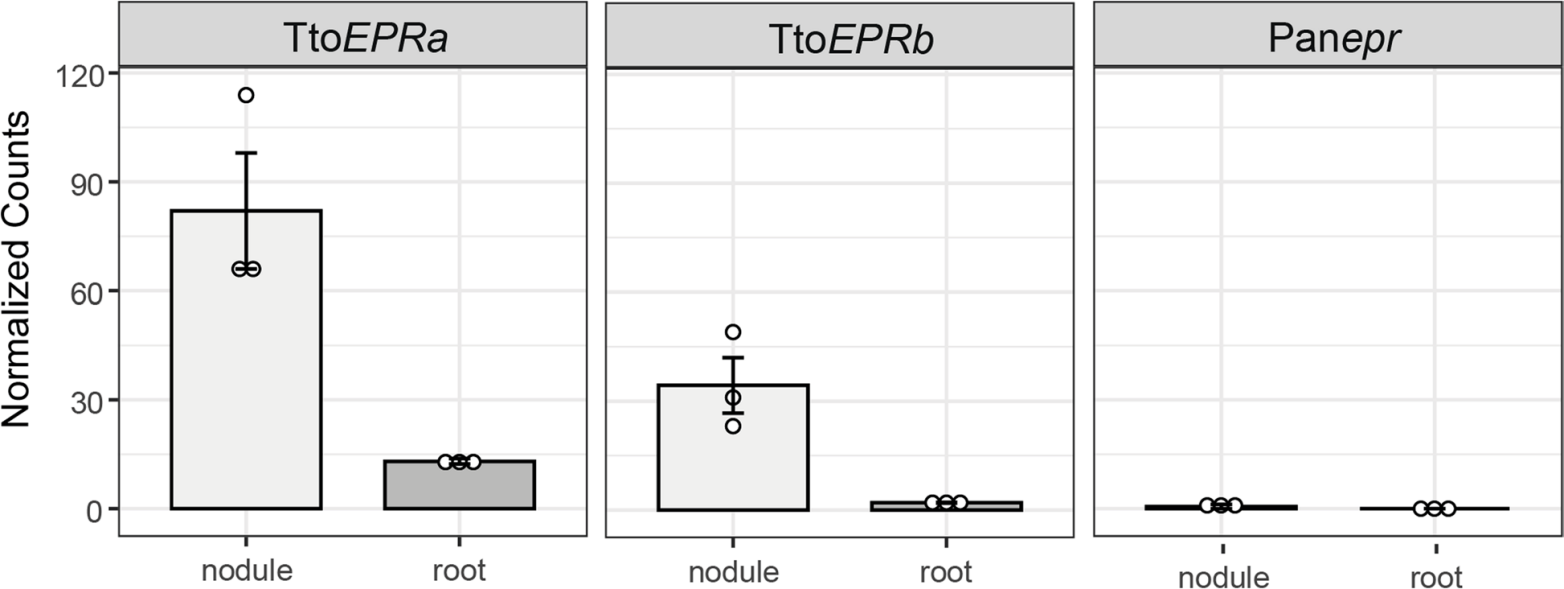
Nodule-enhanced expression of *Trema EPR* in *Parasponia andersonii* (2n) *x Trema tomentosa* (4n) F1 hybrid plants. Hybrid plants are triploid possessing two *T. tomentosa* genes (*TtoEPRa* and *TtoEPRb*) and one *P. andersonii* *epr* pseudogene (*Panepr*). Expression is given in DESeq2 normalized read counts, error bars represent the standard error of three biological replicates, and dots represent individual expression levels

To find additional support for *Trema EPR* expression in nodules, we studied gene expression in an intergeneric F1 hybrid of the cross *P. andersonii* x *Trema tomentosa*. Earlier studies showed such hybrid plants can be nodulated, but are hampered in hosting rhizobium intracellularly [4]. *T. tomentosa* is an allotetraploid. We analysed available genome sequence data and identified two *T. tomentosa EPR* genes, which were named *TtoEPRa* and *TtoEPRb* (Supplemental data file 2). Next, we studied *EPR* allele-specific expression in *P. andersonii* x. *T. tomentosa* F1 hybrid roots and nodules. This revealed a nodule-specific expression of *TtoEPRa* and *TtoEPRb* whereas no expression of *P. andersonii epr* was detected (Fig. 3).

Taken together, expression analysis of the *P. andersonii x T. tomentosa* F1 hybrid as well as pTorEPR:*GUS* reporter studies in *P. andersonii* confirm that *Trema EPR* possesses essential cis-regulatory elements allowing nodule specific expression. This suggests that in a *Trema-Parasponia* ancestor, *EPR* functioned in nodulation.

### The loss of *EPR* in nodulating species is specific to the *Parasponia* lineage

**Fig. 4:**
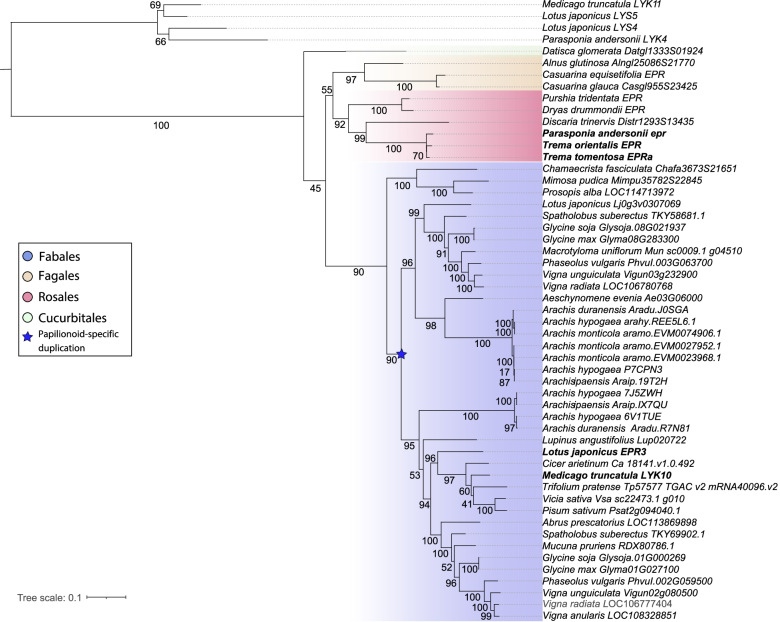
Phylogeny reconstruction of the *LjEPR3/MtLYK10* orthogroup of nodulating species. Color coding indicates taxonomic orders; Cucurbitales (green, 1 species), Fagales (yellow, 3 species), Rosales (red, 4 nodulating species and two non-nodulating Trema species), Fabales (purple, 26 species). Tree rooted on outgroup (*L. japonicus LYS4* and *LYS5*, *M. truncatula LYK11*, and *P. andersonii LYK4*). Asterisk indicates a duplication event in the legume Papilionoideae subfamily. Bootstrap values indicate IQ-tree UF-bootstrap support%; values. The scale bar presents substitutions per site. A complete list of species and accession numbers can be found in Table S1 and Supplemental Data file 3

In *L. japonicus* and *M. truncatula*, *LjEPR3* and *MtLYK10* commit essential functions in rhizobium infection, whereas in *Parasponia* the orthologous gene is pseudogenized. Earlier studies showed that also in the legume *Aeschynomene evenia* the *LjEPR3/MtLYK10* orthologous gene is absent [18]. However, this species possesses a close paralog, which possibly evolved as a result of a legume-specific duplication event and that may commit a similar function [9, 18]. To determine whether loss of *EPR3* occurred more often in nodulating species, we analysed genome sequences of 34 species; 26 legumes (including *A. evenia*, *L. japonicus,* and *M. truncatula*), 7 actinorhizal plant species that nodulate with *Frankia*, and *P. andersonii*. In all species, 1 to 4 putative *ERP3* orthologous genes were identified. Many of these gene models have been predicted based on automated bioinformatics, without manual curation. As *LjERP3*, *MtLYK10* and *TorEPR/Panepr* have a conserved gene structure consisting of 10 exons, we used these to manually curate the gene models in other species (Table S1). This revealed that all species investigated possess at least one gene copy that can encode a LysM-type receptor kinase that in length and structure is comparable to *LjEPR3/MtLYK10/TorEPR*. Subsequent phylogenetic reconstruction, based on a coding sequence alignment and using close paralogs *LjLYS4, LjLYS5, MtLYK11*, and *PanLYK4* as an outgroup, supported the orthologous relation (Fig. 4; Supplemental data file 3). Also, it supports the occurrence of a duplication event in the legume Papilionoid subfamily, and the subsequent loss of one copy in the so-called galagoid clade formed by *Cicer, Medicago, Trifolium, Vicia,* and *Pisum.* As all analysed plant genomes -except *Parasponia*- possess an *EPR3*-type gene, we conclude that loss of this gene in nodulating plant species is uncommon.

## Discussion

The legumes *L. japonicus* and *M. truncatula* use a specific LysM receptor kinase, namely LjEPR3 and MtLYK10, as a secondary identity-based mechanism to control rhizobium infection [6, 9]. We showed that the occurrence of *LjEPR3/MtLYK10* orthologous genes is highly conserved in nodulating plants, including actinorhizal plants that interact with diazotrophic *Frankia*. This suggests that a secondary identity-based mechanism allowing diazotrophic microsymbionts to infect is more generic. Strikingly, however, this mechanism seems to be mutated in *Parasponia* due to the pseudogenization of *LjEPR3/MtLYK10* orthologous gene *EPR*. This raises the question of why this gene was lost in the only non-legume lineage that nodulates with rhizobium?

First, we found that *epr* pseudogenization in the *Parasponia* lineage is associated with a unique retrotransposon insertion near the transcriptional start of the gene. Second, we studied the transcriptional regulation of the *EPR* gene in a nodulation context of the nearest non-nodulating sister species of *Parasponia*; namely *Trema* spp. It is hypothesized that *Trema* spp. lost the nodulation trait after the divergence of *Parasponia*, which is supported by the pseudogenization of several key nodulation genes in *Trema* [4]. We anticipated that *Trema EPR* may still possess the cis-regulatory elements critical for expression upon rhizobium-induced signalling since *T. orientalis EPR* contains putative ERN1 and NIN binding sites in its promoter region, similar as was reported for *L. japonicus EPR3* and *M. truncatula LYK10* [7, 9, 19–22]. Expression analysis using a *T. orientalis EPR* putative promoter GUS (pTorEPR:*GUS*) reporter construct in *P. andersonii* supported this view. The *TorEPR* putative promoter showed to be induced in the first dividing epidermal and cortical cells that occur upon rhizobium inoculation, whereas in the nodule expression is confined in the cell clusters that are about to be infected. Likewise, enhanced expression of the *Trema EPR* alleles was found in nodules formed on *T. tomentosa x P. andersonii* F1 hybrid plants, whereas the *Parasponia* allele is not expressed. Together, these findings support the hypothesis that *EPR* committed a function in nodulation in the last *Trema-Parasponia* ancestor.

Loss of *EPR* in the *Parasponia* lineage may have been instrumental for the microsymbiont switch from *Frankia* to rhizobium. We speculated earlier that such a switch occurred at the base of the *Parasponia* lineage, based on evolutionary signatures in the nodule-specific haemoglobin gene [4, 23]. The EPR receptor of the nodulating *Parasponia-Trema* ancestor could have been co-evolved with its (ancestral) *Frankia* microsymbiont. Such EPR receptor may have hampered the interaction of ancestral *Parasponia* with rhizobium in a somewhat similar manner as observed in *L. japonicus* where LjEPR3 hampers infection of the *M. loti exoU* exopolysaccharide mutant [6]. We initiated the first experiment to find evidence for this hypothesis and introduced the *T. orientalis EPR* gene in *P. andersonii* and quantified the nodulation efficiency of these transgenic lines. Though, despite nodule-specific expression of *TorEPR* (Figure S2), no phenotypes in nodulation were observed (Figures S3 and S4). This suggests that the effect of *trans TorEPR* in *P. andersonii* nodulation is only subtle and difficult to observe under given laboratory conditions. Alternatively, *trans TorEPR* in *P. andersonii* may contribute to the interaction potential withcertain *Frankia* species. Although it is a tempting hypothesis, we considered it experimentally extremely challenging to prove. This is because we anticipate that the ancestral *Frankia* microsymbiont of the nodulating *Trema-Parasponia* ancestor most probably belonged to the taxonomic cluster-2 [13], of which species possess LCO biosynthesis genes [24–26]. *Frankia* cluster-2 strains are notoriously difficult to culture [27], and current (non-sterile) inocula only have limited compatibility with actinorhizal plants of the Southern hemisphere [26, 28, 29].

*M. truncatula* and *L. japonicus*, knock mutants in *Mtlyk10* and *Ljepr3* are affected in the progression of root hair-based infection threads [6, 9]. This results in a reduced number of trans-cellular cortical infection threads. In the *L. japonicus Ljepr3* mutant, successful infection often occurs from large intercellular pockets of bacteria from which subsequently cell penetration can occur (so-called peg infections) [7]. *Parasponia* does not support a root hair-based infection mechanism. Instead, rhizobium enters the roots by a novel crack entry mechanism, not found in legumes or actinorhizal plants. Upon inoculation, root epidermal and most outer cortical cells will divide. The newly formed daughter cells remain only loosely attached creating openings that are colonized by rhizobium [17, 30, 31]. From such an infection pocket, infection threads are formed to enter the nodule primordial cells. In comparison, crack entry infection is also found in some legume species. However, in these cases rhizobium exploits disruptions in the epidermis, e.g. due to later root emergence, rather than actively inducing the formation of such openings [32]. The evolution of this unique crack entry mechanism in *Parasponia* may have coincided with the loss of *epr* and the acceptance of rhizobium as a microsymbiont.

Taken together, this study highlights that LjEPR3/MtLYK10 controlled secondary identity-based mechanism may predate the legumes, as cis-regulatory elements essential for a nodulation associated expression are present in the orthologous gene of *Trema*. Studies in *Parasponia* show, however, that the occurrence of a LjEPR3/MtLYK10-orthologous gene is not essential to allow effective rhizobium nodulation.

## Materials and methods

### Plant materials and nodulation

Experiments were conducted using *P. andersonii* WU1 or its offspring [33] and the interspecific hybrid *P. andersonii x T. tomentosa* line H9 [4]. *P. andersonii* plantlets used for nodulation experiment and qRT-PCR analysis were grown in 1 L clear polypropylene containers allowing for gas exchange (Duchefa Biochemie, The Netherlands). Pots were filled with agraperlite type 3 (Maasmond-Westland, The Netherlands), saturated with EKM nutrient solution (3 mM MES (C_6_H_13_NO_4_) pH 6.6, 0.88 mM KH_2_PO_4_, 2.07 mM K_2_HPO_4_, 2.08 mM MgSO_4_.7H_2_O, 0.7 mM Na_2_SO_4_, 0.375 mM NH_4_NO_3_, 1.45 mMCaCl_2_, 54 µM Fe-citrate, 6.6 µM MnSO_4_, 1.5 µM ZnSO_4_, 1,6 µM CuSO_4_, 4 µM H_3_BO_3_, 4.1 µM Na_2_MoO_4_). For nodulation assay, EKM was inoculated with *B. elkanii* WUR3 (OD_600_ 0.05) [16]. Plants were placed in a conditioned climate room set at 28˚ C and a 16/8 h day/night regime.

### Phylogenetic reconstruction

Protein sequences of publicly available genomes belonging to the Fabid clade (a taxonomic clade within the clade eurosids) were clustered into orthologous groups using Orthofinder (v2.5.1, Emms & Kelly, 2015). The orthogroup containing the *EPR3* orthologues was extracted by searching for th*e L.japonicus LjEPR3* (Lj2g3v14154105) gene name. The EPR3 orthologous proteins were aligned using MUSCLE, and a phylogenetic tree based on this alignment was made using RAxML on the CIPRES Science Gateway version 3.3 [34]. EPR protein alignment was then used to manually curate the data set by assessing the protein model integrity based on the MUSCLE alignment. A protein model was scored complete when all three key EPS receptor domains were present (LysM motifs, trans-membrane and kinase domains). Otherwise, it was scored truncated when missing part of a domain or elongated when it had additional amino acids at the N or C terminal. Any species without a complete orthologous EPR protein model was annotated as a putative gene loss.

### Plant transformation

*P. andersonii* stable transformation was conducted as described in Wardhani et al., (2019) [35]. For the *T. orientalis* promoter the 1,730 bp upstream region was cloned (Supplemental Data File 1) in a Golden Gate compatible level 0 clone (clone i.d. EC74289). This clone was subsequently used to assemble a pTorEPR:*GUS* level 2 binary vector EC74794. using the Moclo backbone pICH86966. As empty vector control, the binary vector EC74842 was used, expressing only a kanamycin resistance gene. Golden Gate constructs used in this study are listed in Table S2. Genotyping of transgenic lines was conducted using the Phire Plant Direct PCR Kit (Thermo Scientific, USA) and specific primers for *T. orientalis* pTorEPR. Amplicons were subsequently confirmed by sequencing.

### Microtome sectioning

Longitudinal sections of root nodules were made from root nodules 5 weeks post-inoculation. Plant tissue was fixed and embedded in technovit 7100 as previously described [35]. Thin Sects. (5 µm) were cut using a microtome (Leica Microsystems, Germany) placed on a glass slide and stained with 0.05% Toluidine blue for imaging. Pictures were taken using a Leica DM5500B microscope coupled with a DFC425C camera (Leica Microsystem, Germany).

### Library preparation and RNA sequencing

Nodules from the two *P. andersonii*stable transformation lines 1.3 and 2.1 containing the pTorEPR*:TorEPR trans* gene, as well as from an empty vector control line, were harvested and flash-frozen in liquid nitrogen in two biological replicates.

Frozen samples were homogenized for 2 min with a bead beater at 2000 rpm and the homogenized sample was immediately resuspended in modified RB buffer, 500 µl RB buffer, 10 µl beta-mercaptoethanol and 50 µl Plant RNA isolation aid (Thermo Fisher Scientific, USA). Then, RNA was extracted using the E.Z.N.A. Plant RNA Kit (Omega Bio-tek, USA) following manufacturer recommendations. Library preparation and sequencing were performed by Novogene (Cambridge, UK). In short, the mRNA is fragmented randomly by adding a fragmentation buffer, then the cDNA is synthesized by using mRNA template and random hexamers primer. Samples were barcoded and pooled according to their effective concentration determined with qPCR and expected data volume. The resulting libraries were sequenced on the Illumina NovaSeq 6000 s platform.

### RNA-sed data analysis and quantification

Paired-end 150 bp “raw reads” quality was assessed with FastQC (v0.11.9). Remaining adaptor sequences were removed from the reads and low-quality reads were filtered out (Q-score < Q20) with Trimmomatic (v0.39). Quantification of transcript abundance was done with Kallisto (v0.46.1) [36] by pseudo-mapping the cleaned reads to *P. andersonii* reference genome [4, 36] with 500 bootstrap replicates; other values were left to default. The Kallisto abundance files were loaded into R version 4.0.2 using tximport [37] and the transcript abundance was normalised using DESEQ2 version 1.34.0 [38].

A draft *T. tomentosa* genome assembly (PRJNA388567) was soft masked using RepeatMasker version 4.0.7 [39]. After softmasking *T. tomentosa* RNA-Seq samples were aligned to the assembly using Hisat version 2.2.1. Gene models for this assembly were generated using BRAKER2 version 2.1.5 [40]. BRAKER2 was trained using the RNA-seq alignment and the proteome of *T. orientalis* and *P. andersonii*. Transcript abundance of the *T. tomentosa* x *P. andersonii* F1 hybrid was quantified using Kallisto version 0.46.2 [36] by simultaneously pseudo mapping with 500 bootstrap resampling; otherwise default values were used. Each RNA-seq sample was separately (PRJNA388743) mapped to the assembled genome of *P. andersonii* and *T. tomentosa*. The Kallisto abundance files were loaded into R version 4.0.2 using tximport [37] and the transcript abundance was normalised using DESEQ2 version 1.34.0 [38]. Differential expression analysis was done by combining each root and nodule sample and comparing the expression between root and nodules.

## Supplementary Information

**Additional file 1:** **Table S1. **Gene structure of *LjEPR3/MtLYK10* orthologs in nodulating plants. Exon lengths indicated in green have been manually curated based on alignment with close homologs. Note: *Trema *spp. represent non-nodulating species.

**Additional file 2:** **Table S2. **Golden Gate compatible plasmids used in this study.

**Additional file 3:** **Figure S1. **The *Parasponia andersonii epr *pseudogene is not expressed. Expression of *Panepr *and its close homologs *PanLYK1, PanLYK3,* and *PanLYK4 *in different tissue types. Expression is given in DESeq2 normalized counts, error bars represent the standard error of 3 biological replicates. RNA-seq data are been described in Van Velzen et al (2018) [4].

**Additional file 4:** **Figure S2. ***TorEPR *is expressed in root nodules of transgenic* Parasponia andersonii* lines. Expression of *T. orientalis* pTorEPR*:TorEPR *in nodules and roots of the *P. andersonii* transgenic lines 1.3 and 2.1, and the empty vector control (ev-control). Expression is given in DESeq2 normalized counts, error bars represent the standard error of two biological replicates. Nodule RNA was isolated 34 days post-inoculation with *Bradyrhizobium elkanii *WUR3.

**Additional file 5:** **Figure S3. ***Trans TorEPR *in *Parasponia andersonii *doesn’t affect nodulation. Light microscopy images of *P. andersonii* transgenic lines harbouring pTorEPR*:TorEPR *thin nodule sections induced with *Bradyrhizobium elkanii *WUR3. (**A**, **B**) Empty vector control line expressing only the kanamycin selection marker. (**C**, **D**) transgenic line 1.3 containing pTorEPR:*TorEPR* (**E**, **F**) transgenic line 2.1. containing pTorEPR*:TorEPR *(**B**, **D**, **F**) Zoom imaging of the infection zone. Scale bars are 100 µm.

**Additional file 6:** **Figure S4. ***Parasponia andersonii* lines expressing *TorEPR *do not reveal a phenotype. (**A**) Plant dry weight boxplot (*n*=6). (**B**) Nodule number on *P. andersonii* root boxplot (*n* = 6). Nodule number is normalized by plant dry weight. (**C**) Total nodule volume per plant boxplot (*n* = 6). Nodule volume is normalized by plant dry weight. Two independent lines (1.3 and 2.1) expressing *TorEPR* have been analysed 34 dpi with *Bradyrhizobum elkanii *WUR3.

**Additional file 7:** **Supplemental data file 1. **Gene sequence of* Trema orientalis EPR, *the *epr* pseudogene of *Parasponia *species, and *LjEPR3 *gene of *Lotus japonicus *in Genbank format.

**Additional file 8:** **Supplemental data file 2. **CDS of *Trema tomentosa EPRa *and *EPRb*.

**Additional file 9:** **Supplemental data file 3. **EPR protein alignment of nodulating plants.

## Data Availability

RNA-seq datasets that were specifically generated and analysed during the current study are available in the NCBI SRA repository bioproject number PRJNA809142. Additionally, we analysed datasets available in bioprojects PRJNA388567, PRJNA272473, PRJNA272473, and PRJNA272482 generated by Van Velzen et al. 2018 [4]. Accession numbers of genes analysed can be found in Table 1.

## References

[1] Doyle JJ (2011). Phylogenetic perspectives on the origins of nodulation. Mol Plant Microbe Interact.

[2] Soltis DE, Soltis PS, Morgan DR, Swensen SM, Mullin BC, Dowd JM (1995). Chloroplast gene sequence data suggest a single origin of the predisposition for symbiotic nitrogen fixation in angiosperms. Proc Natl Acad Sci USA.

[3] Griesmann M, Chang Y, Liu X, Song Y, Haberer G, Crook MB (2018). Phylogenomics reveals multiple losses of nitrogen-fixing root nodule symbiosis. Science.

[4] van Velzen R, Holmer R, Bu F, Rutten L, van Zeijl A, Liu W (2018). Comparative genomics of the nonlegume Parasponia reveals insights into evolution of nitrogen-fixing rhizobium symbioses. Proc Natl Acad Sci U S A.

[5] van Velzen R, Doyle JJ, Geurts R (2019). A resurrected scenario: single gain and massive loss of nitrogen-fixing nodulation. Trends Plant Sci.

[6] Kawaharada Y, Kelly S, Nielsen MW, Hjuler CT, Gysel K, Muszyński A (2015). Receptor-mediated exopolysaccharide perception controls bacterial infection. Nature.

[7] Kawaharada Y, Nielsen MW, Kelly S, James EK, Andersen KR, Rasmussen SR (2017). Differential regulation of the Epr3 receptor coordinates membrane-restricted rhizobial colonization of root nodule primordia. Nat Commun.

[8] Wong JEMM, Gysel K, Birkefeldt TG, Vinther M, Muszyński A, Azadi P (2020). Structural signatures in EPR3 define a unique class of plant carbohydrate receptors. Nat Commun.

[9] Maillet F, Fournier J, Mendis HC, Tadege M, Wen J, Ratet P (2020). Sinorhizobium meliloti succinylated high-molecular-weight succinoglycan and the Medicago truncatula LysM receptor-like kinase MtLYK10 participate independently in symbiotic infection. Plant J.

[10] Becking JH, Stacey G, Burris RH, Evans HJ (1992). The Rhizobium symbiosis of the nonlegume Parasponia. Biological nitrogen fixation.

[11] Ishaq RM, Hairiah K, Alfian I, van Noordwijk M (2020). Natural regeneration after volcanic eruptions: resilience of the non-legume nitrogen-fixing tree Parasponia rigida. Front for Glob Change.

[12] Yang M-Q, Van Velzen R, Bakker FT, Sattarian A, Li D-Z, Yi T-S (2013). Molecular phylogenetics and character evolution of Cannabaceae. Taxon.

[13] Rutten L, Miyata K, Roswanjaya YP, Huisman R, Bu F, Hartog M (2020). Duplication of symbiotic lysin motif receptors predates the evolution of nitrogen-fixing nodule symbiosis. Plant Physiol.

[14] Bailey TL, Williams N, Misleh C, Li WW (2006). MEME: discovering and analyzing DNA and protein sequence motifs. Nucleic Acids Res.

[15] Bailey TL, Johnson J, Grant CE, Noble WS (2015). The MEME Suite. Nucleic Acids Res.

[16] Op den Camp RHM, Polone E, Fedorova E, Roelofsen W, Squartini A, Op den Camp HJM (2012). Nonlegume Parasponia andersonii deploys a broad rhizobium host range strategy resulting in largely variable symbiotic effectiveness. Mol Plant Microbe Interact.

[17] Shen D, Xiao TT, van Velzen R, Kulikova O, Gong X, Geurts R (2020). A homeotic mutation changes legume nodule ontogeny into actinorhizal-type ontogeny. Plant Cell.

[18] Quilbé J, Lamy L, Brottier L, Leleux P, Fardoux J, Rivallan R (2021). Genetics of nodulation in Aeschynomene evenia uncovers mechanisms of the rhizobium–legume symbiosis. Nat Commun.

[19] Breakspear A, Liu C, Roy S, Stacey N, Rogers C, Trick M (2014). The Root Hair “Infectome” of Medicago truncatula uncovers changes in cell cycle genes and reveals a requirement for auxin signaling in rhizobial infection. Plant Cell.

[20] Van Zeijl A, Op den Camp RHM, Deinum EEE, Charnikhova T, Franssen H, Op den Camp HJM (2015). Rhizobium lipo-chitooligosaccharide signaling triggers accumulation of cytokinins in Medicago truncatula roots. Mol Plant.

[21] Jardinaud M-F, Boivin S, Rodde N, Catrice O, Kisiala A, Lepage A (2016). A laser dissection-RNAseq analysis highlights the activation of cytokinin pathways by nod factors in the Medicago truncatula root epidermis. Plant Physiol.

[22] Liu CW, Breakspear A, Guan D, Cerri MR, Jackson K, Jiang S (2019). NIN acts as a network hub controlling a growth module required for rhizobial infection. Plant Physiol.

[23] Sturms R, Kakar S, Trent J, Hargrove MS (2010). Trema and parasponia hemoglobins reveal convergent evolution of oxygen transport in plants. Biochemistry.

[24] Persson T, Battenberg K, Demina IV, Vigil-Stenman T, Heuvel BV, Pujic P (2015). Candidatus Frankia datiscae Dg1, the actinobacterial microsymbiont of Datisca glomerata, expresses the canonical nod genes nodABC in symbiosis with its host plant. PLoS One.

[25] Nguyen TV, Wibberg D, Battenberg K, Blom J, Vanden Heuvel B, Berry AM (2016). An assemblage of Frankia Cluster II strains from California contains the canonical nod genes and also the sulfotransferase gene nodH. BMC Genomics.

[26] Van Nguyen T, Wibberg D, Vigil-Stenman T, Berckx F, Battenberg K, Demchenko KN (2019). Frankia-enriched metagenomes from the earliest diverging symbiotic Frankia cluster: they come in teams. Genome Biol Evol.

[27] Gtari M, Ghodhbane-Gtari F, Nouioui I, Ktari A, Hezbri K, Mimouni W (2015). Cultivating the uncultured: growing the recalcitrant cluster-2 Frankia strains. Sci Rep.

[28] Silvester WB (1977). Dinitrogen fixation by plant associations excluding legumes. Treatise on Dinitrogen Fixation.

[29] Benson DR, Silvester WB (1993). Biology of Frankia strains, actinomycete symbionts of actinorhizal plants. Microbiol Rev.

[30] Lancelle SA, Torrey JG (1984). Early development of Rhizobium-induced root nodules of Parasponia rigida. I. Infection and early nodule initiation. Protoplasma.

[31] Lancelle SA, Torrey JG (1985). Early development of Rhizobium-induced root nodules of Parasponia rigida. II. Nodule morphogenesis and symbiotic development. Can J Bot.

[32] Sprent JI (2007). Evolving ideas of legume evolution and diversity: a taxonomic perspective on the occurrence of nodulation. New Phytol.

[33] Op den Camp R, Streng A, De Mita S, Cao Q, Polone E, Liu W (2011). LysM-type mycorrhizal receptor recruited for rhizobium symbiosis in nonlegume Parasponia. Science.

[34] Miller MA, Pfeiffer W, Schwartz T. Creating the CIPRES Science Gateway for inference of large phylogenetic trees. In: 2010 Gateway Computing Environments Workshop (GCE). ieeexplore.ieee.org; 2010. p. 1–8.

[35] Wardhani TAK, Roswanjaya YP, Dupin S, Li H, Linders S, Hartog M (2019). Transforming, genome editing and phenotyping the nitrogen-fixing tropical Cannabaceae tree Parasponia andersonii. J Vis Exp.

[36] Bray NL, Pimentel H, Melsted P, Pachter L (2016). Near-optimal probabilistic RNA-seq quantification. Nat Biotechnol.

[37] Soneson C, Love MI, Robinson MD (2015). Differential analyses for RNA-seq: transcript-level estimates improve gene-level inferences. F1000Res.

[38] Love MI, Huber W, Anders S (2014). Moderated estimation of fold change and dispersion for RNA-seq data with DESeq2. Genome Biol.

[39] Smit AFA, Hubley R, Green P. 2013--2015. RepeatMasker Open-4.0. 2021.

[40] Brůna T, Hoff KJ, Lomsadze A, Stanke M, Borodovsky M (2021). BRAKER2: automatic eukaryotic genome annotation with GeneMark-EP+ and AUGUSTUS supported by a protein database. NAR Genom Bioinform.

