# Supplementary material for: Pseudogenization of the rhizobium-responsive EXOPOLYSACCHARIDE RECEPTOR in Parasponia is a rare event in nodulating plants
Source: BMC Plant Biol. 2022 Apr 30;22:225. doi: 10.1186/s12870-022-03606-9 (PMC9055685; doi:10.1186/s12870-022-03606-9)
Supplement: Supplementary file 2 — Additional file 2: Table S2. Golden Gate compatible plasmids used in this study. [file 12870_2022_3606_MOESM2_ESM.docx]

**Table S2: Golden Gate compatible plasmids used in this study**

| Level 0 Constructs synthesized | | | |  |  |  |  |
| --- | --- | --- | --- | --- | --- | --- | --- |
| Construct Number | **Gene Name** | **properties/function** | **Module Type** | **overhang 5'3'** | **overhang 5'3'** | **Backbone** | **Antibiotic** |
| EC74218 | TorEPR | cds | CDS1 | AATG | GCTT | lifetechL0 | spec |
| EC74289 | TorEPR | promoter-5’UTR | PU | GGAG | AATG | lifetechL0 | Kan |

| Level 1 modules | | | | | | | | |
| --- | --- | --- | --- | --- | --- | --- | --- | --- |
| Construct  Number | Backbone | Purpose | L2 direction:position | Antibiotic resistance | promoter + utr | gene | tag/fusion (Nt-Ct) | terminator |
| EC74577 | pICH47802 | plant selection marker | R1 | Ampicillin | pICH7004 kanamycin cassette | | | |
| EC74565 | pICH47742 | TorEPR | F2 | Ampicillin | PU TorEPR EC74218 | TorEPR CDS EX74289 |  | Tnos pICH41421 |

| Level 2 binary constructs | | | | | | | | | | | |
| --- | --- | --- | --- | --- | --- | --- | --- | --- | --- | --- | --- |
| Construct Number: | **Purpose** | **Backbone** | **Vector**  **Res** | **Pos1** | **Pos2** | **Pos3** | **Pos4** | **Pos5** | **Pos6** | **Pos7** | **EL** |
| EC74842 | Control | pICSL4723 | Kan | EC74577 Kan | - | - | - | - | - | - | EL-1 |
| EC74792 | TorEPR introduction | pICSL4723 | kan | EC74577 Kan | EC74565 TorEPR | - | - | -- | - | - | EL-2 |

| Level 2 Binary constructs single insert | | | | | | | | |
| --- | --- | --- | --- | --- | --- | --- | --- | --- |
| Construct  Number | **Backbone** | **Purpose** | **fixed insert** | **Antibiotic resistance** | **promoter + utr** | **gene** | **tag/fusion (Nt-Ct)** | **terminator** |
| EC74794 | pICH86966 | TorEPR:GUS | pNOS:kan:tNOS | Kanamycin | PU TorEPR EC74218 | GUS pICH75111 |  | T35s pICH41414 |
