# Supplementary material for: Pseudogenization of the rhizobium-responsive EXOPOLYSACCHARIDE RECEPTOR in Parasponia is a rare event in nodulating plants
Source: BMC Plant Biol. 2022 Apr 30;22:225. doi: 10.1186/s12870-022-03606-9 (PMC9055685; doi:10.1186/s12870-022-03606-9)
Supplement: Supplementary file 3 — Additional file 3: Figure S1. The Parasponia andersonii epr pseudogene is not expressed. Expression of Panepr and its close homologs PanLYK1, PanLYK3, and PanLYK4 in different tissue types. Expression is given in DESeq2 normalized counts, error bars represent the standard error of 3 biological replicates. RNA-seq data are been described in Van Velzen et al (2018) [4]. [file 12870_2022_3606_MOESM3_ESM.pdf]

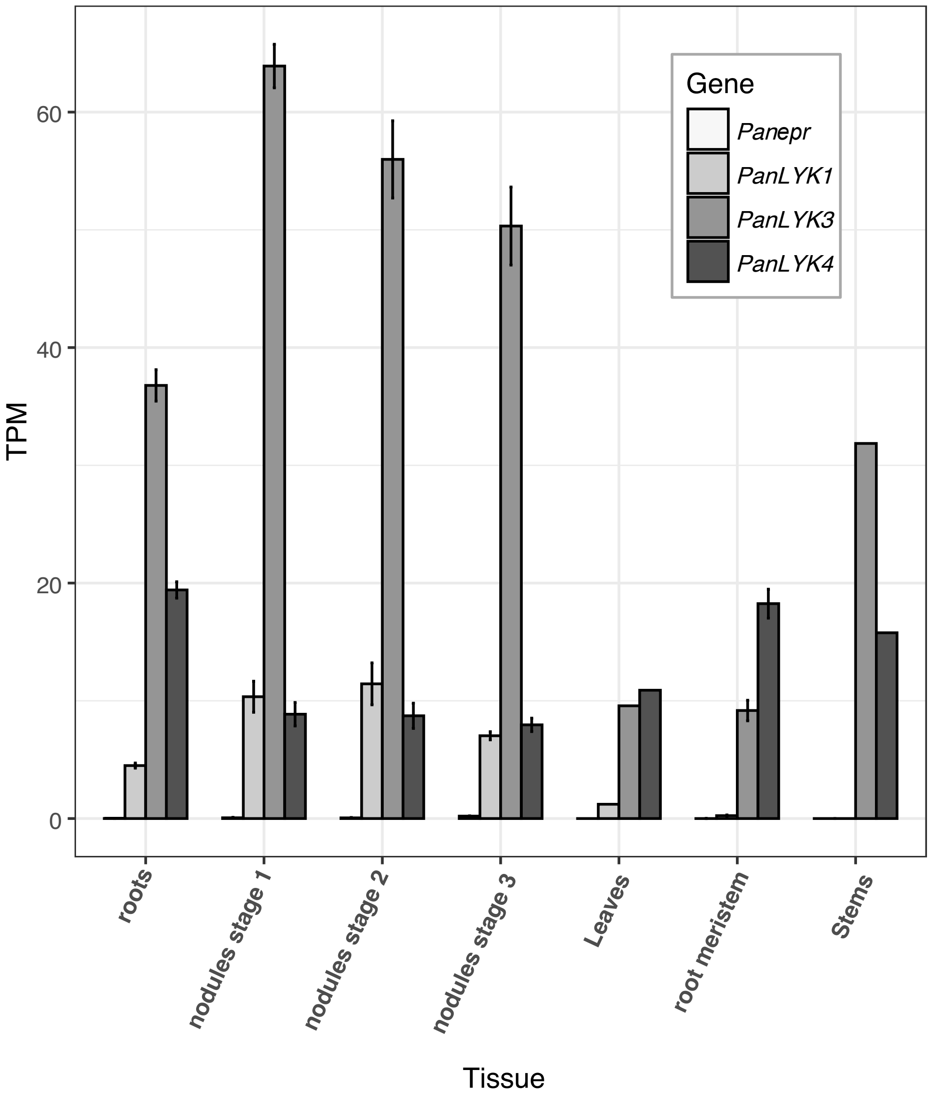

Figure S1: The *Parasponia andersonii Panepr* pseudogene is not expressed. Expression of *Panepr* and its close homologs *PanLYK1*, *PanLYK3*, and *PanLYK4* in different tissue types. Expression is given in DESeq2 normalized counts, error bars represent the standard error of 3 biological replicates. RNA-seq data are been described in Van Velzen et al (2018).
