# Supplementary material for: Pseudogenization of the rhizobium-responsive EXOPOLYSACCHARIDE RECEPTOR in Parasponia is a rare event in nodulating plants
Source: BMC Plant Biol. 2022 Apr 30;22:225. doi: 10.1186/s12870-022-03606-9 (PMC9055685; doi:10.1186/s12870-022-03606-9)
Supplement: Supplementary file 4 — Additional file 4: Figure S2. TorEPR is expressed in root nodules of transgenic Parasponia andersonii lines. Expression of T. orientalis pTorEPR:TorEPR in nodules and roots of the P. andersonii transgenic lines 1.3 and 2.1, and the empty vector control (ev-control). Expression is given in DESeq2 normalized counts, error bars represent the standard error of two biological replicates. Nodule RNA was isolated 34 days post-inoculation with Bradyrhizobium elkanii WUR3. [file 12870_2022_3606_MOESM4_ESM.pdf]

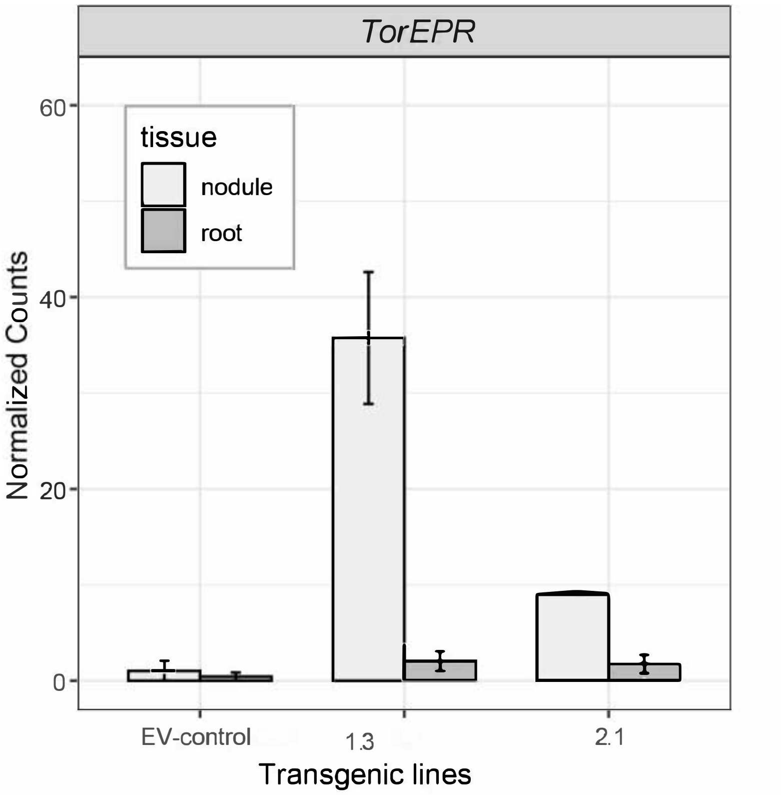

Figure S2: *TorEPR* is expressed in root nodules of transgenic *Parasponia andersonii* lines. Expression of *Trema orientalis* pTorEPR*:TorEPR* in nodules and roots of the *P. andersonii* transgenic lines 1.3 and 2.1, and the empty vector control (EV-control). Expression is given in DESeq2 normalized counts, error bars represent the standard error of two biological replicates. Nodule RNA was isolated 34 days post-inoculation with *Bradyrhizobium elkanii* WUR3.
