# Supplementary material for: Pseudogenization of the rhizobium-responsive EXOPOLYSACCHARIDE RECEPTOR in Parasponia is a rare event in nodulating plants
Source: BMC Plant Biol. 2022 Apr 30;22:225. doi: 10.1186/s12870-022-03606-9 (PMC9055685; doi:10.1186/s12870-022-03606-9)
Supplement: Supplementary file 5 — Additional file 5: Figure S3. Trans TorEPR in Parasponia andersonii doesn’t affect nodulation. Light microscopy images of P. andersonii transgenic lines harbouring pTorEPR:TorEPR thin nodule sections induced with Bradyrhizobium elkanii WUR3. (A, B) Empty vector control line expressing only the kanamycin selection marker. (C, D) transgenic line 1.3 containing pTorEPR:TorEPR (E, F) transgenic line 2.1. containing pTorEPR:TorEPR (B, D, F) Zoom imaging of the infection zone. Scale bars are 100 µm. [file 12870_2022_3606_MOESM5_ESM.pdf]

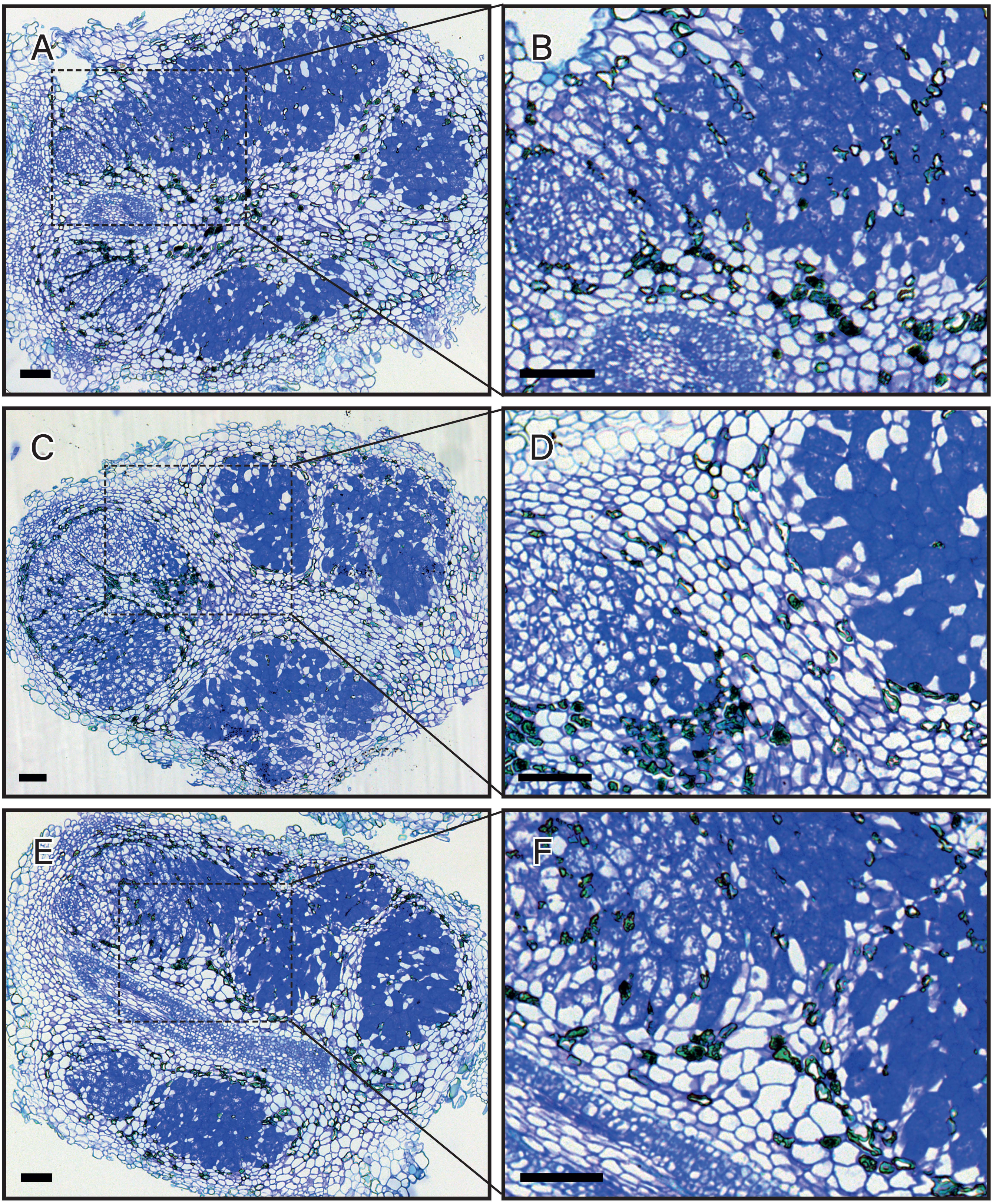

Figure S3: ***Trans TorEPR* in *Parasponia andersonii* doesn't affect nodulation.** Light microscopy images of *P. andersonii* transgenic lines harboring pTorEPR:*TorEPR* thin nodule sections induced with *Bradyrhizobium elkanii* WUR3. (**A, B**) Empty vector control line expressing only the kanamycin selection marker. (**C, D**) transgenic line 1.3 containing pTorEPR*:TorEPR* (**E, F**) transgenic line 2.1. containing pTorEPR:*TorEPR* (**B, D, F**) Zoom imaging of the infection zone. Scale bars are 100 µm.
