# Supplementary material for: Pseudogenization of the rhizobium-responsive EXOPOLYSACCHARIDE RECEPTOR in Parasponia is a rare event in nodulating plants
Source: BMC Plant Biol. 2022 Apr 30;22:225. doi: 10.1186/s12870-022-03606-9 (PMC9055685; doi:10.1186/s12870-022-03606-9)
Supplement: Supplementary file 6 — Additional file 6: Figure S4. Parasponia andersonii lines expressing TorEPR do not reveal a phenotype. (A) Plant dry weight boxplot (n=6). (B) Nodule number on P. andersonii root boxplot (n = 6). Nodule number is normalized by plant dry weight. (C) Total nodule volume per plant boxplot (n = 6). Nodule volume is normalized by plant dry weight. Two independent lines (1.3 and 2.1) expressing TorEPR have been analysed 34 dpi with Bradyrhizobum elkanii WUR3. [file 12870_2022_3606_MOESM6_ESM.pdf]

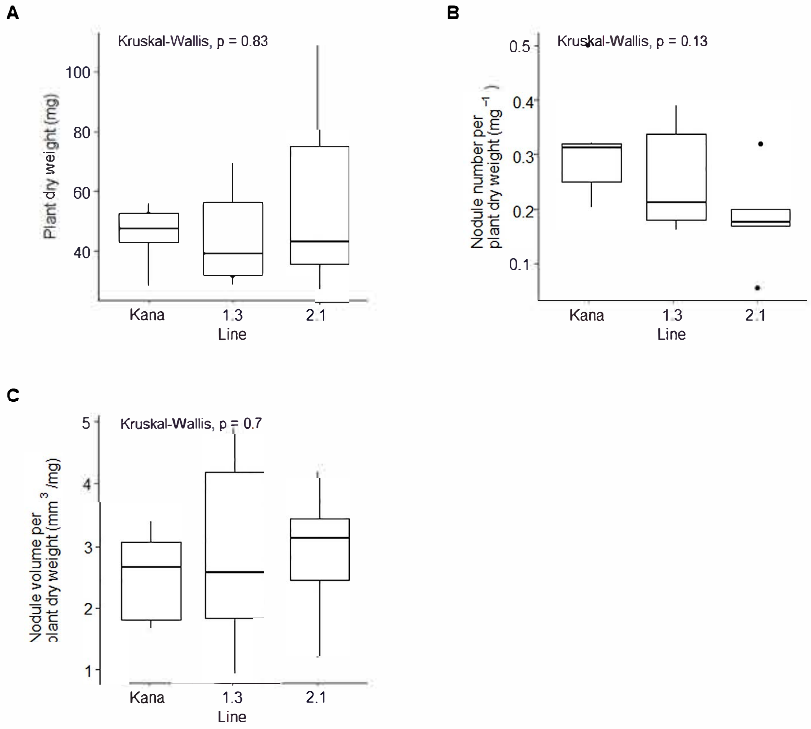

Figure S4. *Parasponia andersonii* lines expressing *TorEPR* do not reveal a phenotype. (**A**) Plant dry weight boxplot (n=6). (**B**) Nodule number on *P. andersonii* root boxplot (n = 6). Nodule number is normalized by plant dry weight. (**C**) Total nodule volume per plant boxplot (n = 6). Nodule volume is normalized by plant dry weight. Two independent lines (1.3 and 2.1) expressing *TorEPR* have been analysed 34 dpi with *Bradyrhizobum elkanii* WUR3.
