# Supplementary material for: Pseudogenization of the rhizobium-responsive EXOPOLYSACCHARIDE RECEPTOR in Parasponia is a rare event in nodulating plants
Source: BMC Plant Biol. 2022 Apr 30;22:225. doi: 10.1186/s12870-022-03606-9 (PMC9055685; doi:10.1186/s12870-022-03606-9)
Supplement: Supplementary file 7 — Additional file 7: Supplemental data file 1. Gene sequence of Trema orientalis EPR, the epr pseudogene of Parasponia species, and LjEPR3 gene of Lotus japonicus in Genbank format. [file 12870_2022_3606_MOESM7_ESM.docx]

**Supplemental data file 1: Gene sequence of *Trema orientalis TorEPR,* the *epr* pseudogene of *Parasponia* species, and *LjEPR3* gene of *Lotus japonicus* Genbank format.**

LOCUS TorEPR_flanks_(reversed) 6035 bp DNA linear UNA 26-JUL-2021

DEFINITION TorRG33x02_asm01_scf00021:1095001..1105280.

ACCESSION .

VERSION .

KEYWORDS .

SOURCE .

ORGANISM Trema orientalis

FEATURES Location/Qualifiers

Promoter 405..2145

/created_by="User"

/modified_by="User"

/label="Putative promoter"

CDS join(2146..2794,3049..3252,3424..3554,3624..3713,

3803..3908,4282..4433,4654..4751,4866..5004,5414..5512,

5771..5935)

/created_by="User"

/modified_by="User"

/label="CDS"

gene 2146..5935

/created_by="User"

/label="TorEPR"

ORIGIN

1 gcataaaacc gtgttgaatg gattttcctt ctctatggac gtaagagtta attcatgttc

61 attcactgat cggtttgtgc atggttgttt gcctgaggct tttcaatctc ttctgtctgt

121 ttgttgtggc tgttttaatg ggaggtagag ttgagattct gtttggtttc ttgtttttga

181 atgctttgaa ggcattatta ttagtttaag ttctaaatat agtttttcta gattttgtta

241 tgaccagtaa ttgaagaaga ggtctataag agcaaagact tctggggttg caaacagaaa

301 tgaaatattg caatattggt tgatcttggt ccgaaagaca aggtcgagac cggagattgc

361 cttaatgttg tgtgtcattt cctagtttgt ccgtgagaaa agagtcccag aaagcacact

421 aaattgtggt tcgattactt aatttggtca atggtttggt ttaagtatct taagtggcga

481 attcggattt gctttattaa gattggtact tacaaatctt cgttctccat tagataagtt

541 gctccatctt caaattaata tgtttaaaaa gatagtatta aagaattctg cggtagacca

601 aaggtccaaa gtgagatgat cattatagtc cgtttttgga tccacggcca agatatggag

661 ctcacaagtc aaacgtcgat ctgaatcata ggtgtgcaag tggaaccatt ccaaaacttt

721 cctacatgga aagaccaatg gaccaggcct gacaaactga tgggttggac ttaattggac

781 tcttttcatt cgtaggaagt gggcctgcta taatgtggta ggccttctgt cggacttgtc

841 ctccgaagta tatcctcctc caaaatgcat aatgtaactt gtcctggtcc tgaaatggca

901 aaatgtatgt agtttccttt agccggcaaa gaatatctat ccacagttgc atacaataag

961 aaaagactag tcagcacttg tttctgactc cctaattcta ataataattt aattaagttt

1021 cattacctct ttgttgattt aggagggcag tttgtttttc tcttgcttga caatattttg

1081 gactgctgct tagagtcaat actagactca agtacagtat ttagattgtt ttgctatgaa

1141 aagagaaaat aaaacaggag agagggtgaa atcaatgaaa ccaagtgaga aaaaaagggc

1201 tttcccacca aggagaatac cacattcatc ttattcattg acatagaatg aattaaaaag

1261 acagaacaaa cttagccctt catagagttg aacaggcctt attatttggg gtttgactta

1321 aagaaaatca tgttcttgtc cacataaaac aaatttccga aaatggactt aggaatagcc

1381 aaccatgcta gaagggccct tgtaattaat gttactatca aaattggctt acatggcaat

1441 tattgcatca caattaatta tttttgtaga cccataatgt tgaaatatga gcctatgaga

1501 atggatgatt gtgagcatag ttcatgttac aataaggctg gtttatatga attattgggt

1561 tacttatatg tccacaaaat tgtaagtaaa acttacactt acgtcttatg agcattgtat

1621 tggagcatgt aacactgccc gttaaagtaa agctgtatgt cgtgtaagtg ttgaactctg

1681 tttcatttag attgggaaat ttatggacat ttgactttag cttgttgtct gctaatacga

1741 cccggccatt cctgatacca tgaaaaagcc gtttgatgtt atcagatgaa aaagttgtaa

1801 tcatcgatga gatattcttt atataaaaat cctatctgaa aagagatttc tgtataaata

1861 acgataccct gttactctct ttgtctgata ttatacaact cactctctcc ctctctctct

1921 caaacaaaca cacacacaca cacttgtaac tcagcatttc tctatttaaa atgtcattta

1981 gaccaatgaa ctgcttttgt ttattgaaaa aacaaacaaa ccagttataa caaagagaac

2041 aaagtatggt gtgtaactcc aacattgcca aactttgttg tgtaacgcca acattgccat

2101 tacttctttc tgttcttcaa agagaaaaat ccaatgccaa aaaccatggc aacccatcat

2161 ctccttccct atcttctcct tatgttccct ctttgttctc gagtttttac actccatgtg

2221 tctatgaaag aatcccttat gtaccctttt agctgctcag cacaaatcaa gacgtgtaat

2281 gcttcattgt accatattaa tgaaggtctc acaatagaag aaattgctgc ttattacaag

2341 gtcaacgcat cctcccaaat tgagcccata atgcatgaca gcaggaaaga ttacctcata

2401 acagtacctt gttcctgctc atccatgttt ggcgtaaccg gatattttta caacacaacc

2461 tacaacgtca aattaaatga cacttttcta gatgtttcag ctaagtacta tagtggacaa

2521 gcttggagat ttgaagaaga agaccaatat ttcaagcctg ataaaaattt taccatgcat

2581 cttctttgtg gttgtctaga aagtgactcc gaaattgtgg taacctacac agttcaggac

2641 catgatacac tactaaatat tgcagctcgg ttgtcagcca atttcgacaa cttggtgaga

2701 ttgaatggac atttgactca aaaccctgct ttcattttgg taggctgggt cttgtttgtg

2761 cccaaggaga aaaatggaat tgaaacatca acgggtgagc tctgtttgat atatggaaca

2821 aatcatgggc ttgaaaatta aaaattgctt ggtcaaaaca catacacaca cacacacaca

2881 cgtctatatg tatgtatatc tgttttgagt aagaatgcat aagaaatgaa atacatctat

2941 acgtgatata cttacatgca ggaatcccag aatttagtac acgataattg atgatccttt

3001 ttaggaagtt aactatataa tgttgttgtt tattttcttt gccatcaggt atgagagaga

3061 agtggaaaat tgtaactggt atattgttaa ttgtgacgct gctttcaact ggtgcattga

3121 tggtcattct tctcagaaga aaaagattgc agcaaaagaa agtggaagat cctaaagtca

3181 tatccaaaat tgtgactttt aaaaaatctc tctccttgca gaatcctttc ctccctaaag

3241 aaaattttga aggtgagacc aaaaaaaaaa aaaaaaaaac acttgactta tgtccaaaac

3301 tagttttatt tatgtattga tctatgctta atatgccttg tattagctat gactagaaga

3361 ctttctggac taagatagat gattacggtt atactaatcc atgaaattat atatatgtgg

3421 cagattttga ttcagaaaaa actgtagtat ttagtcttga ggcgatcgaa gaggccactg

3481 gacactttga cgaaagtaag aaagtaggag aggggggata tgggtgtgtg tattttggca

3541 tactaggaga gaaggtatgg aaagtctcct ctacttattt ttaggttact catgactgaa

3601 aatgctaaac atttcaactt caggaggtgg ccataaagaa gatgatatcg aataaatcga

3661 aggagttctt tgcagagcta aaggtgttat gcaagatcca tcatataaac gtggtgagta

3721 aacaaataaa ttccatacaa gctcttttca ctaatatgtt gaaaggaaaa gctctcattc

3781 acatgcaaaa atttgtcttt aggtggagct tttggggtat gctagtggag atgaccacct

3841 ctacttggtt tacgagtatg ttccgaacgg atcactgagc gatcatcttc atgatccgtt

3901 actgaaaggt atttcgagta actggtgcaa atgcttttga gtttgttgga cgaattttct

3961 tggctcaaaa aattcagaga gtacttatct tttttctttc tgtgctagca ccaaaaatat

4021 ggaggctttg catatttatc tagagcaaga ttctagtgct ctttaccaga atacatctta

4081 attattaggc tttaaaaggg ctttatccat tcatccattc ctgttcaatg aacgagatta

4141 ttagcacaac attgcatgcc tccaattcat aaataatttc tcaagataga agttcaaaac

4201 ttcacttgag ctagaatcct tgtcttaagc tgtaaagcaa aaaaataaga aattgactag

4261 aaaagaatgc tattgaatca ggtcaccagc ctctgtcctg gactgcaaga gcacagattg

4321 cacttggcgc tgcaaacggt attgagtaca ttcatgacca tacaaaagca cggtatgtgc

4381 accgtgatat aaaaacaact aacatcctac ttgatgaggg cctcagagct aaggtatcta

4441 aatcatgatc ctgtataata tcaggaaatg cttatccgtt actcctatga tcaagttagc

4501 tatttcaaac cagttagttc cataatagtc atcttccgct atatgacaaa ttctgataaa

4561 aatttgagta gacaaccatg tttagatata acatagctct agttttaaaa ctatatacta

4621 aaactgacca ctactcggca tccgtttcaa caggtagcag attttgggtt agcaaagctt

4681 gttgaacgag ccaatgaaga tgatttcata gcgacacgac tagttggcac accaggctac

4741 cttcctccag agtaagttca atattacagt ggactcaagt tctcttcgat atcatttcga

4801 tcattttctc atgcaaacaa aatatactac tttgtttctc acattctata aaaatctttt

4861 gaaagatcgg tgaaggagct acaggtaacc ccgaagacag atgtatttgc atttggagtg

4921 gtactagcag agctgattac agggcaacgc gcacttatcc gtgacaaccg agagcctaac

4981 aagatgaaat ctctaaaaac agttgtaagt tgaaaagtat aattccttat gaaaagagaa

5041 aacaagatga aaatcattaa cagaacacat tatcttcttg aaaagctttt gtcccagccg

5101 ataacatgcg ggtcttttag gcctgcaatt tgaagctgcc ttgtctatca aagcattcaa

5161 agaacttttc cctatcgaat gagattggct cttcattgtt atataggaac ataacaaaag

5221 aaaagaaaag aaaatacaaa tcaaatttct ttgttgtatc ttcaattgtg aacatgaggc

5281 ctgcaatgca catacatgac atgtctgtgc aatcaggact gtccatcata ataatgcgtt

5341 acggctaaca aaatcctttt gaacgtaact aagaactaaa gaagctgact tctcttttgc

5401 accacatgat caggttaaga gaatattcca agatgaagat ccagaatcag ctttagatgc

5461 cgaaatagac ggaaatctca ggggcagcta ccctagtgag gatgtcttca aggttggtca

5521 cccaactcta agagtctcct ttatcttcct catttgagac atagatttaa cagagaaatg

5581 cccattattg ttatcaaagt tttattatgt tctgatcttt atatttgaaa aagttctaaa

5641 cacaagtttt caaggacccc atttatagaa ctaaaaattt aacagtgccc aactcaaaga

5701 attacctagt gggactttgt tttgtatata aagatgcctt attcttgctg ttgttggtac

5761 tatgtggcag atggcagaag ttgcggagtt gtgcttacgt gaagaagcag tggacagacc

5821 agagatgagg gatattgtgg tgacactgtc tcaaatagtg atgtcctcga tagagtggga

5881 agcatcactt ggagggaaca gccaggtctt cagtggggta ttcactggca gatgaacata

5941 ttttatctca gatgtatact attttttcag tgatacctta ctctatatac atatacatat

6001 atgtatatat acacacagct ttaaacaacg acagc

//

LOCUS Pruepr_flanks 10212 bp DNA linear UNA 04-NOV-2020

DEFINITION .

ACCESSION .

VERSION .

KEYWORDS .

SOURCE .

ORGANISM Parasponia rugosa

FEATURES Location/Qualifiers

Promoter join(191..>1884,<7647..7800)

/created_by="User"

/modified_by="User"

/label="Putative promoter"

misc_feature 1885..7646

/created_by="User"

/label="Retrotransposon"

/note="Geneious type: sequence variant"

CDS join(9927..10091,9565..9663,9012..9150,8800..8897,

8428..8579,7801..>8127)

/created_by="User"

/modified_by="User"

/label="CDS"

gene join(7801..>8127,<8128..10091)

/created_by="User"

/modified_by="User"

/label="Pruepr"

ORIGIN

1 aaccgtgttg aatggatttt ccttctcttt ggacgtaaca gttaattcat gttcattcac

61 tgatcggtta ttgcatggtt gtttgcttaa ggtttttcaa tctcttctgc ctgcttgttg

121 tggctgtttt aatgggaggt agagttgaga ttctgtttgg tttcttgttt ttgaatgctt

181 tgaaggcatt attattagtt taagttctaa atatagtttt ttctagattt tgttatgacc

241 agtaattgaa gaagaggtca ataagagcaa agacttctgg ggttgcgaac agaaatgaaa

301 tattgcaata ttgtttgatc ttggtccgaa agacaaggtc gagaccggag attgccttaa

361 tgtcgtgtgt catttcctag tttgtccggg agaaaagagt cccaaaaagc cactaaattg

421 tggttccatt acttaatttg gtcaatggtt tggttcaaat atcttaagtg gcgaatttgg

481 atttgcttta ttaagattgg tacttacaaa tctacgttct ccattagata agttgctcca

541 tcttcaaatt aatacgttta aaaagatact attaaataat tctgcggtag accaaaggtc

601 caacgtgaga tgatcattat cggtttttgg atccattgcc aagatatgga gctcacaagt

661 caaaagtcaa tctgaatcat aggtgtgcaa ctggaaccgc caatccaaaa gtttcctaca

721 tggaaagacc aatggaccag gcttgacaaa ctgatgggtt ggacttaatt ggactctttt

781 cattcgtagg aactgggcct gctataatgt agtaggcctc cggttggact tgtccttcta

841 agtagatcct cctccaaaat gcataatcca acttgtcctg gtcctgaaat ggcaaaatgc

901 atgtagtttc ctttagccgg caaagaatat ctatgcatag ttgcatacaa taagaaaaga

961 ctagtcagca cttgtttctg actccctaat tctaataata atttaattaa gtttcattac

1021 ctctttgttg attcaggagg gaagtttgtt tttctcttgg ttgacaatat tttggaccgc

1081 tgcttggatt caataataga ctcaagtaca gcatttagat tgctttgcaa tgaaaagaga

1141 aaataaaaca ggagagatgg tgaaatcagt gaaaccaagt gagaaaaaaa gggctttccc

1201 accaaggaga aaaccacatt cgtcttattc attgacatag aatgaattaa aaagacagaa

1261 caaacttagc ccttcgtaga gttgaacagg ccctattatt tggggtttga cttaaagaaa

1321 atcatgttct tgtccacata acacaaattt ccgaaaatgg acttaggaat agccaaccat

1381 gctagaaggg cccttgtaat taatgttact accaaaattg gcttacatga caattatcac

1441 attcatttat ttttgtagac ccataatgct gaaatatcag ctatggatga ttgtgagcat

1501 agttcatttt acactaagac tggtttagat gaattattgg gttacttata tgtccacaaa

1561 attgtaagta aaacttacac ttacgtctta tgcgcattgt attggagcat gtaacactgc

1621 ccgtcaaagt aaagctgtat gtcgtgtaag cgttgaactc tgtttcattt agattgagaa

1681 attttatgga catttgactt tagcttgttg tctgataata cgacccggcc attcctgata

1741 tcatgaaaaa tccgtttgat gttatcagat gaaaaagttg taatcatcga tgagatgttc

1801 tttatataaa aatcccatct gaaaagagat ttatgtataa ataacaatac cttgttactc

1861 tctttgtctg atattataca actctttctt tttcacgcag cgaattgtta aatttcgctc

1921 taacttgcgt tttcacaatg agaagaaaaa cgaataaaat tctcaaagag aatttagaag

1981 agaaaactaa gatgtgattg aagattagat gattacaact cttgtcaatg aaagttattt

2041 atacaagttt aagattaatc taaaccatcc aataaaataa ttctaagaaa cttacaacca

2101 tatacctagt acctaccaac caaacacact taaataaata aattgttcac ctagaatact

2161 ttaaataaac aaattgttca cataacacac gtcaaaaaaa taataaacaa gctaaataac

2221 ctaatacatt ttaaataaat aagtctataa tacctaacac acaaaagtat tctaccaacc

2281 aatgaaatgt tcattagatt gggtcttgga attttggacc ttggtttagg tcttatatgt

2341 ttgtgggcta ctgcatcagt tctccccctc ttgaaaaaaa aaaacttgtc ctcgagttac

2401 tcaaggtatg actttcggtc catgatgctc gtataggtct tgaacattga aggtcttaga

2461 aattatgtaa ctatctggta gatcaatgat atatgtttta tcattaattt tttgaataat

2521 gcgagagagg ctaattttct tttgcttgag tttattgtat tcttttgttg gaagtctttc

2581 tttacataaa tgaaacatta taaaatcgtc atctttgaaa gttttgaatc ttctatgctt

2641 gtcagcatta gtcttgtatt ttgcagtaga ctattctaac tttcttttca ctttttcatg

2701 gatgtggatc gtcttattaa tcaaatgatc gacaataatg ctcatccctg aaagttttgg

2761 taaagatacc aagtcgagag tatgcaacgg aggacgaata taaacaattt caaataaata

2821 tttactgctg gaataatttg gcacattgtt gtaagcaaac ttagcttgtg gtaggatatt

2881 ccctattgtc tagggctttc tttgatcaag caccgtaaaa tgttccccag tgtgtgatta

2941 gtgactttag tttgctcatc tatttgggga tgacaagtgc tactaaattg gaaggtgata

3001 tcaagtctcg tccataaagt atgccaaaaa tgactgagga atttactatc acgatcagag

3061 actattgtcc ttggtaacca tgcagactga ggaatttact atcacgatca gagactattg

3121 tccttggtaa cccatgcaga cgaacaatct cttgaaagaa caattgagca atataggtag

3181 catctgaagt tttcttgcag ggaataaaat aagccatttt tgaaaaacga tctaccacaa

3241 caaagataga atctgaacct ttttgtgttc gcagtagtcc taagatgaaa tccatgaaca

3301 aatcttccca atgtgcatca ggaataaaaa gggacatgta taacatagtg ttttgaactt

3361 gtccctttgt agtctggcat atataacaat atttgacaaa tttctcaaca tcatgcctaa

3421 gatatggcca aaagtattta gcttccacta atgagatagt ttttcctcgt ctcatgtgtc

3481 ctcctaaccc tcctgcatat agctcctgaa tgatgtgttc tctgagagat cctcgtagaa

3541 tacataaacg gatttgaaag tatagaaaac catcatgtat ttgatgtttt tttgcaactc

3601 cactaatgca cttctcccat tcttccttga aatcttcacc atcagtgtat aagtctttta

3661 aataatcaaa atcgattatc tcattttgaa caacagtcaa taatataact cgatgactca

3721 atacattggc aacgttgttt aattatcctg acttatgcct gagagaaaaa ttgaactttt

3781 ggatataggc aatccaccga gcatgcattc gatttagttt agaagagttg ttgaggtact

3841 taagagcttg atgatttgta ttgaggatga attccttctg tactagatgt gactcttatt

3901 gttgtaatgt acgaacaacc gtatataatt cttgttcgta cgttgaccac ttctttctgg

3961 cttccctaag cttttcacta aagaaagcta tcggcttcat atcttgagat agaacagctc

4021 taactcctac gccagaagca tcacagtcta gttcgaaaat tttattgaag tcataaagtg

4081 cacggatggg tgtatttata agtttttact tcaagatagc aaaactctgt tccacttctt

4141 ctgtccaaaa gaattgcttt tgcttcaaac attcagtgag tggctatagt actaaagttt

4201 ctcacaaatc atctatcaga acgttgcaag acataaaaac tccgaatttc tgtaatggtc

4261 ttcagtattg gctattctcg gatagctctt acctaatttt gatcaatttt gattccatca

4321 gaacttacaa taaagcctaa aaagactaag ctatcagtca tgaagctaca tttttcaggt

4381 tgacatacaa ctaattctta taaagtgtat taaaaacttc ggtcaagtgt tgtagatgtt

4441 cttttttatt ctgattatag accaaaatat catcaaaata aacgatgaca aatcttccaa

4501 tgaaggggtt gaaatacctg attcattaac ctcataaaag tactcgggac attggataga

4561 ccgaatgaca taactaacca cttgtatagt ctttcatgta ttttgaatgt agtcttccat

4621 tcatctccag gatgaatacg aatctgatgg tatccactct gcaaatcaat tttggaaaag

4681 agctttgatc ctcccaatct atcaagtatg tcatccaatc gtggaataga aaatcgatat

4741 cgtatcgtaa tcttgttgat ggcctgacta tcaatgcaca tcctctaact ttcgtctttc

4801 ttcggtatta ggagtgcagg tacaaccata gaactcatac tttctctaat catccctttc

4861 ttgaggaggt tttcgactta tatttgtagg atctcatact ccttaagact tatgtggtag

4921 tacagtagac ttgccctagg aataagatca atctagtatt gaatatttcg taggggtggt

4981 aagctagcag gtaacttctc aggaacgatg tcttcaaatt cagacaataa tatttgaact

5041 tacacaggaa tattggtagg agtatatact tttttaggta caaaaagaag gttgtttggt

5101 tattttgcac cttcatagga gctagaataa tttttatatt atccttatgg aagctatttg

5161 tgttagcttt tccattatgc atagtgttta aatcgaattg ccatggttga ccaagcaata

5221 tatgacaagt atctatatca aaaacatcac ataatacctc atctttgtat ttatcgatgg

5281 agaaaggaat ttgacattgt tatgtcacct ttatttcacc gacgtctttg atctagccaa

5341 tgcgatacat ggcagtatat ggttgggttg gtatttataa cttgtccatc atagtcttaa

5401 atactatgtt ctcactactc ctattatcaa tgagaagatc acacactcat tgttcaacca

5461 tataatgagt ctcgaagatg ttgtatcatt gtatgtcttc ttccttcttt agtgtataca

5521 tcaatcgtct tatcatcaag gaaatatctt catctccata aataatttct tcaggttcaa

5581 cttcatctac gtattcctca ttcttgaaat acgcctcgtc cccttcgaca tcatcatcat

5641 atgtggttag attaataggt ttattctacc gacattgatt tgaatagtag cttgtttgtc

5701 cacaacgata atacttatta cctctcagga ctgcataagg gttgttagtg ttgttgttgg

5761 ttattttcct ttgtagtcga gcagttggtt gattgtgaga aggagttggt cttactaatt

5821 cgtcgtgtgg tcttgctatg gccttttctt tattcaaatc agatttttat ggtggtgtag

5881 aagattgaga gattgttgaa ttacgatttg ctcctttgtt gagctgagtt tcaattttca

5941 aagtgagttg aattacacta gacaaagtat tatgagatta tttgaatacc tcgtcttata

6001 tgtttgcacg aagtccatca atgaatctag aaatcttgta actttcagat ttgcgaatat

6061 ccactcgggt agttagtcta tgaaactcgg ttatgtattc ttgtactgtc cagtatgctt

6121 gttggcagtg ttgatatcga ctaaaaagtg cttgtttgta atttggaggt agaagtgctc

6181 gattatcaat tgtttaatcc gtcgccaagt gtaaactttg tttctccctt accgaatacg

6241 tgtcgctttt aattgatccc accaagttaa tgcacctcct ttgagtcgat aagccactaa

6301 tttgactttc aagtattctg gagtgcccta ataatcaaag aacttttcta ctttagctag

6361 ctaattaagg aattcttcaa tcacgaatag atcattaaat tacagaatct cgatcttcat

6421 gcgatagtta ttttgccgaa gtccttggtt gtttctgagc gtagctggtt cttcatggtc

6481 ttcactagaa tctttatctt gaaaaaattg attttgatat gtctaaaatt tccaaaagga

6541 tcaacttctt catgtctctg tcgtttataa ttgttgtttc gtttcctaag aaagtgttga

6601 gtcaaatttg taagcattgt ctctaaattc tgcaatcttc tatcaagtgc attcactgac

6661 tcttctgttg caaactcttc tacaacggga ttttgttggc tagactctcc atccgacatt

6721 tttttatata tttttttacc tttttttttt gcttttatag gaaagaagat agtataaact

6781 tttaaaagta aagaaaaata taattatttt tttttactct ttttattttt ttttgctttt

6841 agaggaaaga aaatagtaca aactcttaaa agtaaagaaa aataaaactc tcaaaaattt

6901 aaacagtaaa aaaaaaatta aagattataa aaatatatat attaccttgt taaaggaatg

6961 aagcaaattc tctaaggagg aaaccttgaa gcaaattctc taaggaggaa accttgatat

7021 acacttcttg tatttttttt tttcggaaac aaagatagtg tatgaaagga ttatatatgt

7081 atgtgtggta aataataatt gtaggaaggt gatagtaaag attagatagc gaaggtgatg

7141 ataaaataag gatttttttt tatgaaaata atgtacagtg acgggaatag gaaagctctg

7201 tctgatacag aggtagtctc tgataccaat tgacgcagcg gattactaga tttcgctcta

7261 acttgcgttc tcacaacgaa aagagaaaca agtaaagttc tcaaagagaa tttaaaagag

7321 aaaactaaga tgtgattaaa gattggatga ttacaactct tgctaacgaa ggctatttat

7381 acaagtttag aattaatcta aaccatccaa taaaataatt ctaagaaact tacaatcata

7441 cacctgatat ctaccaacta aacatactta aataaataaa ttgttcacct tgaatacttt

7501 aaataaacaa attgttcaca taacacattt caaataaata agctaaagaa cctaatacat

7561 tttaaataaa taagtctata atacctaata cacaaaaata ttctactaac caataaaata

7621 gattgagttt tagaattttg ggcatttgct tttgtttatt gaaaaaacaa acaaaccagt

7681 tatgacaaag agaacaaagt atggtgtgta actccaacat tgccaaactt tgttgtgtaa

7741 cgccaacgtt gccattactt ctttctgttc ttcaaagaga aaaatccaat tccaaaaacc

7801 atggcaaccc atcatctcct tccctatctt ctccttatgt tccctctttg ttctcgagtt

7861 tttacactcc atgtgtctat gaaagaatct cttatgtacc cttttaactg ctcggcacaa

7921 atcaaggcgt gtaatgcttc attgtaccat atcaatgaag gtctcacaat agaagaaatt

7981 gctgcttatt acaaggtcaa cgcatcctcc caaattgagt ccataatgca taacaacagg

8041 gaagattacc tcataacagt tccttgttcc tgctcatcca tgtttggcgt aaccggatat

8101 ttttacaaca cgacctacaa cgtcaaggag tacttatctt tttcttcatg tgctagcact

8161 gaaatatgga ggctttgcat atttacctag agcaagattc tagtgctgtt taccagaata

8221 catcttaatt aacaagtctt agaagggctt tattcattca tacattcttg ttcaatgaat

8281 gagattatta gcacaacatt gcaagccttc aattcagaaa taatttctca agatagaact

8341 tcaaaacttc acttgagcaa gaatccttgt cttacgctgt aaagcaaaaa ataagaaatt

8401 gactagaaaa gaatgctatt gaatcaggtc accagcctct gtcctggact gcaagagcac

8461 agattgcact tggcgctgca aagggtattg agtacattca tgaccataaa aaagcacggt

8521 atgtgcaccg tgatataaaa acaactaaca tcctacttga tgagggcctc agagctaagg

8581 tatctaaatc atgatcctgt ataatatcaa gaaatgctta tccgttactc ctatgatcaa

8641 gttagctatt tcaaaccagt tagttccatg atagtcatct tccgctacat gacaaattct

8701 gataaaaatt tgagtagaca accatgttta aatataacat agctctagtt ttaaaactat

8761 atactaaaat tgaccactac ccggcatcca tttcaacagg taacagattt tgggttagca

8821 aagcttgttg aacgagccaa tgaagatgat ttcatagcga cacgactagt tggcacacca

8881 ggctaccttc ctccagagta agttcaatat tacagtggat tcaagttctc ttcgatatca

8941 tttcgatcat tttctcatgc aaacaaaata tactacattg tttctcacat tctataaaat

9001 tcttttgaaa gatcggtgaa ggagcttcag gtcaccccga aaacagatgt atttgcattt

9061 ggagtggtac tagctgagct gattacaggg caacgcgcac ttatccgtga caaccgagag

9121 cctaacaaga tgaaatctct aataacagtt gtaagttgaa aactataatt ccttatgaaa

9181 agagaaacaa gatgaaaatc attaacaaaa cacattatct tcttgaaaag cttttgtccc

9241 agccaataac atgtggtttc ttctaggcct gcaatttgaa gctgtcttgt ctatcaaaga

9301 atccaaacaa cttttcccta tcgaatggga ttggcttggc tcttcattgt tatataggaa

9361 cattacaaac ataaagaaaa gaaaatacaa atcaaagttc tgtattgtat cttcacttgt

9421 gaacatgagg cctgcaatgc acatacatga catgtctgtg caatcaggac tgtccatcat

9481 aataatgcgt tacggctaac aaaatccttt tgaacgtaac taagaactaa agaagctgac

9541 ttctgttttg caccacatga tcaggttaaa agaatattcc aagatgaaga tccagaatca

9601 gctttagaag ccgaaataga cggaaatctc cggggcagct accctagtga ggatgtcttc

9661 aaggttggtc acccaactct acgagtctcc tttatcttcc tcatttgaga catagattta

9721 acagagaaaa gcccattatt gttatcgaag ttttattatg tactgatctt tatacttgaa

9781 aaagttcaaa acacaagttt tcaaggaccc catttataga actaagaatt taacagtgcc

9841 caactcaaaa aattacctag tgggactttg ttttgtatat aaataaagat agtcttattc

9901 ttgatgttgt tggtactatt tggcagatgg cagaagttgc ggagttgtgc ttacgtgaag

9961 aagcagtgga cagaccagag atgagggata ttgtggtgac actgtctcaa atagtgatgt

10021 cctcaataga gtgggaagca tcacttggag ggaacggcca ggtcttcagt ggggtattca

10081 ctggcagata aacatatttt ttctcagatg tatactattt tttcaataat accttactat

10141 atatacatat acatatatgc atatatacac aaagctttaa acaacaacaa cctggacagg

10201 atggagaaaa aa

//

LOCUS Priepr_flanks 11440 bp DNA linear UNA 04-NOV-2020

DEFINITION .

ACCESSION .

VERSION .

KEYWORDS .

SOURCE .

ORGANISM Parasponia rigida

FEATURES Location/Qualifiers

Promoter join(74..>1768,<7445..7598)

/created_by="User"

/modified_by="User"

/label="Putative promoter"

misc_feature 1769..7444

/created_by="User"

/label="Retrotransposon"

/note="Geneious type: sequence variant"

CDS join(7599..8248,8500..8703,8868..8998,9068..9157,

9247..9352,9729..9880,10101..10198,10313..10451,

10866..10964,11228..11392)

/created_by="User"

/label="CDS"

gene 7599..11392

/created_by="User"

/label="Priepr"

ORIGIN

1 ttgtggctgt attaatggga ggtagagttg agattctgtt tggtttcttg tttttgaatg

61 ctttgaaggc attattatta gtttaagttc taaatatagt ttttttctag attttgttat

121 gaccagtaat tgaagaagag gtcaataaga gcaaagactt ctggggttgc gaacagaaat

181 gaaatattgc aatattgttt gatcttggtc cgaaagacaa ggtcgagacc ggagattgcc

241 ttaatgtcgt gtgtcatttc ctagtttgtc cgggagaaaa gagtcccaaa aagccactaa

301 attgtggttc cattacttaa tttggtcaat ggtttggttc aaatatctta agtggcgaat

361 ttggatttgc cttattaaga ttggtacgta caaatctacg ttctccatta gataagttgc

421 tccatcttca aattaatacg tttaaaaaga tactattaaa taattctgct gtagaccaaa

481 ggtccaaagt gagatgatca ttatccgttt ttggatccat tgccaagata tggagctcac

541 aagtcaaaag tcaatctgaa tcataggtgt gcaactggaa ccgccaatcc aaaagtttcc

601 tacatggaaa gaccaatgga ccaggcttga caaactgatg ggttggactt aattggactc

661 ttttcattcg taggaactgg gcctgctata atgtagtagg cctcctgttg gacttgtcct

721 tctaagtaga tcctcctcca aaatgcataa tccaacttgt cctggtcctg aaatggcaaa

781 atgcatgtag tttcctttag ccggcaaaga atatctatgc atagttgtat acaataagaa

841 aagactagtc agcacctgtt tctgactccc taattctaat aataatttaa ttaagtttca

901 ttacctcttt gttgattcag gaaggaagtt tgtctttctc ttggttgaca atattttgga

961 ccgctgcttg gattcaataa tagactcaag tacagcattt agattgcttt gcaatgaaaa

1021 gagaaaataa aacaggagag atggtgaaat cagtgaaacc aagtgagaaa aaaagggctt

1081 tcccaccaag gagaaaacca cattcgtctt attcattgac atagaatgaa ttaaaaagac

1141 agaacaaact tagcccttcg tagagttgaa caggccctat tatttggggt ttgacttaaa

1201 gaaaatcatg ttcttgtcca cataacacaa atttccgaaa atggacttag gaatagccaa

1261 ccatgctaga agggcccttg taattaatgt tactaccaaa attggcttac atgacaatta

1321 tcacaatcat ttatttttgt agacccataa tgctgaaata tcagctatgg atgattgtga

1381 gcatagttca ttttacacta agactggttt agatgaatta ttgggttact tatatgtcca

1441 caaaattgta agtaaaactt acacttacgt cttatgagca ttgtattgga gcatgtaaca

1501 ctgcccgtca aagtaaagct gtatgtcgtg taagcgttga actctgtttc atttagattg

1561 agaaatttta tggacatttg accttagctt gttgtctgat aatacgaccc ggccattcct

1621 gatatcatga aaaatccgtt tgatgttgtc agatgaaaaa gttgtaatca tcgatgagat

1681 gttctttata taaaaatccc atctgaaaag ggatttatgt ataaataaca ataccttgtt

1741 actctctttg tctgatatta tacaactctt tctttttcac gcagcgaatt gttaaatttc

1801 gttaacttgc gttttcacaa tgagaagaaa aacgagtaaa attctcaaag agaatttaga

1861 agagaaaact aagatgtgat tgaagattag ataataacaa ctcttgtcaa tgaaagttat

1921 ttatacaagt ttaagattaa tctaaatcat ctaataaaat aattctaaga aacttataac

1981 catacaccta atacttacca accaaacaca cttaaataaa taaattattc acctagaata

2041 ctttaaataa acaaattgtt catataacat acgtcaaaaa ataataaata agctaaataa

2101 cctaatacat ttcaaataaa taagtctata atacctaaca cacaaaatta ttctaccaac

2161 caatgaaatg gtcattgcat tggtcttgga attttggacc ttggctttag gtcttatatg

2221 tttgtgggct actgcattag ttctcccctt attgaaaata aaaaaacttg tcgtcgagtt

2281 actcaaggta tgacttccgg tctatgatgc tcgtataggt cttgaacatt gaaggtctta

2341 gaaattgtgt aactatctga tagatcaatg aaatatgttt tatcattaat tttttgaatg

2401 atgcgagaga ggccaatttt cttttacttg agtttattgt attttcctgt tggaagtctt

2461 tctttacata aatgaaacat tacaaaatcg tcctctttga aagttttgaa tcttctgtgc

2521 ttattagcat tagccttgta ttttgcagta gactcttcta actttctttt cactttttca

2581 tggatgtgga tcgtcttatt aatcaaatga tcaacaataa tgcttatccc tgaaagtttg

2641 gttaaagata ccaagtcgag agtatgcaac ggaagacgaa tataaacaat ttcaaataaa

2701 gatttactgt tggaataatt tggcacgttg ttgtaagcaa acttagcttg tggtaggata

2761 ttctccattg tctagggctt tctttgatca agcaccgtaa aatattcccc agtgtgtgat

2821 tagtgacttt agtttgctca tctatttggg gatgacaagt gctactaaat tggaaggtga

2881 tatcaagtct catccacaaa gtatgccaaa aatgactgag gaatttacta tcgcgatcaa

2941 agactattgt ccttggtaac ccatgcagac gaacaatctc tcgaaagaac aattgagcaa

3001 tataggtagc atctgaagtt ttcttgtagg gataaaataa gccatttttg aaaaatgttc

3061 taccacaaca aagatagaat ctaaacattt ttgtgttcgc agtagtccca agatgaaatc

3121 catgaacaaa tcttcctaag gtgtatcagg aataaaaagg gacatgtata acctagtgtt

3181 ttaagcttgt ccctttgtag tctggcatat ataacaatat ttgacaaatt tctcaacatc

3241 atgcctaaga tgtggccaaa agtatttagc ttccactaat gaaatagttt tttctcgtct

3301 catatgtctt tctaaccttc ctgtgtatag ctcatgaatg atgtgttctc tgagagatcc

3361 tcgtagaata cataaacgga tttgaaagta tagaaaacca tcatgtatta tatatttttt

3421 tgcaacttca ctaatgcact tctcccattc ttccttgaaa tctttaccat caatgtataa

3481 gtctttcaaa taatcaaaat cgattatctc attttgaaca acagtcaata gtataactcg

3541 atgactcaat atatcagcaa cattgtttaa ttatcctgac ttatgcctga gaggaaaagt

3601 aaacttttgg atataggcaa tctatcgagc atgcattcga tttagtttgg aagagttgtt

3661 aaggtactta agagcttgat gatctgtatt gaggatgaat tccttctgta ctagatgtgg

3721 ctctcgttga tgtaatgtat gaacaactgt atataattct tattcgtacg ttgaccactt

3781 ctttctggct tcactgagct tttcactaaa gaaaactatc ggctttatat cttgagctag

3841 aacagctcta actcctacgc cagaagtatc atagtctact tcaaaaattt tatcaaattc

3901 ataaagtgca cggataggtg tatttataag tttttacttc aagatagcaa aactctattc

3961 cgcttcttct gtccaaaaga attgcttttg ctttaaatat tcggtgagtg atgcagctat

4021 agtactaaag tttctcacaa atcatctata gaacgttgca agacatgaaa actccgaatt

4081 tctgtaatgg ttttcagtat tgaccattct cggatagccc ttaccttatt ttgatcaatt

4141 ttgattccat cagaacttat aataaagcct aaaacaacta agttgtcagt catgaagcta

4201 cattttttag gttgacatac aactaattct tataaagtgt attaaaaact tcggtcaagt

4261 gttgtagatg ttctttttga ttctgattat aaaccaaaat atcatcaaaa taaacgacga

4321 caaatcttcc aatgaagggg ttgaaatacc tgattcatta atctcatgaa agtactcggg

4381 acattggata gaccgaatga cataactaac cactcatgta gtctttcatg tattttgaat

4441 gtagtcttcc attcatctcc aggatgaata cgaatctgat ggtatccgct ccgcaaatca

4501 attttggaaa agagctttga tcctcccaat ctatcaagta tgtcatccaa tcgtaggata

4561 gaaaatcgat atcatatcgt aatcttattg atggcctgac tatcaatgca catcctctag

4621 ctttcgtctt tcttctgtat taggagtgta ggtataacca tggaactcat actttctcta

4681 atcatccctt tcttgaggaa gttttcgact tatatttgta ggatctcata ctccttaaga

4741 cttatgtggt agtacagtaa acttgctcta ggaataagat caatctagta ttgaatattt

4801 cgtaggggtg gtaagctagc aggtaactcc tcaggaacga tatcttcaaa ttcaaacaat

4861 aatatttgaa cttgcacagg aatattggta ggagtatata cttttttagg tacaaaaaaa

4921 aggttgtttg gttattttgc tgcttcatag gagctagaat aatttttata ttatccttat

4981 ggaagctgta tgtgttagct tttccattat gcatagtgtt taaatcgaat tgccatggtt

5041 gaccaagtaa tatatgacaa gtatctatat caaaaacatc acataatacc tcatctttgt

5101 atttatcgat ggagaaagga atttgatatt gttatgtcac ctttatttca ccgacgtctt

5161 tgatctagca aatgcgatac gtggcagtat atggttgggt tggtattggt aacttgttca

5221 tcatagtctt gaatactatg ttctcactac tcctattatc aatgagaaga tcacacactc

5281 attgttcaac catacaatga gtctcgaaga tgttgtgtca ttacatgtct tcttccttct

5341 ttagtgtata catcaatcgt cttatcatca aggaaatatc ttcatctcca taaataactt

5401 cttcaggttc aacttcatct acgtactcct cattcttgaa atatgccttg tccccttcga

5461 catcatcatc atgtgtaatt agattaatag gtttattcta ccgacattga tttgaatagt

5521 ggcctatttg tccacaacga taatatttat tacctctcaa gactgcataa gggttgttag

5581 tattgttgtt ggtcatttcc ctttgtagtc gggtggttgg ttgattatga gaaggagttg

5641 gtcttactaa ttcatcgtgt ggtcttgcta tggcctttcc tttattcaaa tcagattttt

5701 atggtggtgt agaagattga gaaattgttg aattacgatt tgctcctttg ttgagctgag

5761 tttcaatttt caaagcgagt taaattacac tagacaaagt attataagat tatttgaata

5821 cctcgtctta tatgtttgta cgaagtccat caatgaatct agaaatcttt taactttcag

5881 atttacgaat atctactggg gtagttagtc tatgaaactc ggttacgtat tcttgtattg

5941 tccagtatgc ttgttggcag tgttgatatt gactaaaaag tgcttgtttg taatttggag

6001 gtagaagtgc ttgatcatca attatttcat ccgtcgccaa gtgtaaactt tgtttctcct

6061 ttatcgaata cgtgtcgctt ttaattgatc ccaccaagtt aacgcacctc ctttgagtcg

6121 ataagccact aatttgactt tcaagtattc tggagtacct taataatcaa agaacttttc

6181 tacttcagct agctaattaa ggaattcttc aatcacgaat agatcattaa attacagaat

6241 ctcgatcttc atgcgatagt tattttgccg aagtccttgg ttgtttttga gtgtggctgg

6301 ttcttcatgg tctttactag aatctttatc ttgaaaaaat tgattttgat gtgcctaaaa

6361 tttccaaaag gatcaacttc ttcatgtctc tgtcgttcat aattgttgtt tcgtctccca

6421 agaaagtgtt gagtcaaatt tgtaagcatt gtctctagat tctgcaatct tctatcaagt

6481 gcatccactg actcttctgt tgcaaactct tctacagcag aattttgttg gctagacttt

6541 ccatccgaca ttttttttac cttttttttt tttttaactt ttataggaaa gaagatagta

6601 taaactttta aaagtaaaga aaaatataat tacatttttt tactcttttt ttttttttgc

6661 ttttagagga aagaaaatag tacaaacttt taaaagtaaa aaaaaaatag aactctcaaa

6721 aatttaaaca gtaaaaaaaa aaattaaaaa ttacaaaaat gtatatatta ccttgttaaa

6781 ggaatgaaga aaattctcta aggaggaaac cttgatatac acctcttgta tttttttttt

6841 ttcggaaaga aagatagtgt ataaaaggtt tatatgtgta tatgtgataa ataattattg

6901 taggaaggtg atggcaaaga ttagatagcg aaggtgatga tgaaataagg atttttttaa

6961 taaaaataat gtacagtgac gggaatagga aagctctgtc tgatacagag gtagtctctg

7021 gtaacaatta actcagcgaa ttactagatt tcgctctaac ttgtgttctt acaacgagaa

7081 gagaaacaag taaagttctc aaagagaatt taaaagagaa aactaagatg tgatcaaaga

7141 ttggatgatt acaactcttg ccaacgaatg ctatttatat aagtttagga ttaatctaaa

7201 ccatccaata aaataattct aagaaattta taaccataca catgatatct accaaccaaa

7261 tatacttaaa taaataaatt gttcaccttg aacactttaa ataaacaaat tgttcacgta

7321 acacatttca aataaataag ctaaagaact taatacattt taaataaata agtctataat

7381 acctaataca taaaaatatt ctactaacca ataaaataga ttgagtttta gaattttggg

7441 catttgcttt tgtttattta aaaaagaaac aaaccagtta tgacaaagag aacaaagtat

7501 ggtgtgtaac tccaacattg ccaaactttg ttgtgtaacg ccaacgttgc cattacttct

7561 ttctgttctt caaagagaaa aatccaattc caaaaaccat ggcaacccat catcgccttc

7621 cctatcttct ccttatgttc cctctttgtt ctcgagtttt tacactccat gtgtctatga

7681 aagaatctct tatgtaccct tttaactgct cggcacaaat caaggcgtgt aatgcttcat

7741 tgtaccatat caatgaaggt ctcacaatag aagaaattgc tgcttattac aaggtcaacg

7801 catcctccca aaattgagtc cataatgcat aacaacaggg aagattacct cataagagta

7861 ccttgttcct gctcatccat gtttggcgta accggatatt tttacaacac gacctacaac

7921 gtcaaattaa atgacacttt tctagatgtt tcagctaagt actataatgg acaagcttgg

7981 agatttgaag aagaagacca atatttcaag cctgataaaa attttaccat gcatcttctt

8041 tgtggttgtc tagaaagtga ctccggaatt gtggtaacct acacagttca ggaccatgat

8101 acactattaa atattgcaaa tctgttgtca gccaaattcg acaagttggt gagattgaat

8161 ggatatttga ctcaaaaccc tgctttcatt gagctaggct gggtcttgtt tgtgcccaag

8221 gagaaaaatg gaattaaaac atcaacgggt gagctctgtt tgatatatgg aacaaatcat

8281 tggcttgaaa attgaaaatt gcttagccaa aacacacaca cacacgcgcg cacgtctata

8341 tgtatgtata tctgttttga gtaagaatgc ataagaaatg aaatacatct atacgtgaca

8401 tacttgcatg caggaatccc agaatttagt acacgataat tgatgatcct ttgtaggaag

8461 ttcactatat atgttgttgt ttattttctt tgccatcagg tacgagagag aagtggaaaa

8521 ttgtaactgg tatattgtta attgtgacgc tgctttcaac tggtgcattg atggtcattc

8581 ttctcagaag aaaaagattg cagcaaaaga aagtggaaga tcctcaagtc ctatcaaaaa

8641 ttgtgactac taaaaaatct ctctccttgc agaatccttt ccttcctaaa gaaaattttg

8701 aaggtgagac cgaaaaaaaa aaaacacttg cttatgtcca aaactagttt tatttatgta

8761 ttgatctatg cttaatatgc cttttattag ctatgactag aagactttct ggactaagag

8821 agatgattac ggttatacta atccatgaaa ttgtatatat gtggcagatt ttcattcaga

8881 aaaaactgta gtatttagtc ttgaggcgat cgaagaggcc actggacact ttgacgaaag

8941 taagaaagta ggagagggag gatatgggtg tgtgtatttt ggcataatag gagagaaggt

9001 atggaaagtc tcctctactt atttttaggt tactcatgac tgaaaatgct aaacatttca

9061 acttcaggag gttgccataa agaagatgag atcgaataaa tcgaaggagt tctttgcaga

9121 gctaaaggtc ttatgcaaga tccatcatat aaacgtggtg agtaaacaaa taaattctat

9181 acaagctctt ttcactgata tgttgaaagg aaaagctctc attcacatgt aaaaatttgt

9241 ctttaggtgg agcttttggg gtatgctagt ggagatgacc acctctactt ggtttacgag

9301 tatgatccga acagatcact gagcgatcat cttcatgatc cgttactgaa aggtatttcg

9361 agtaactggt gcaaatgctt ctgagtttgt tggatgaatt ttcttggctc aaatacataa

9421 aattcaagga gtacttatct ttttcttcat gtgctagcac tgaaatatgg aggctttgca

9481 tatttaccta gagcaagatt ctagtgctgt ttaccagaat acatcttaat taacaagtct

9541 tagaaggact ttattcattc atacattctt gttcaatgaa tgagattatt cgcacaacat

9601 tgcaagcctt caattcagaa ataatttctc aagatagaac ttcaaaactt cacttgagca

9661 agaatccttg tcttacgctg taaagcaaaa aataagaaat tgactagaaa agaatgctat

9721 tgaatcaggt caccagcctc tgtcctggac tgcaagagca cagattgcac ttggcgctgc

9781 aaagggtatt gagtacattc atgaccatac aaaagcacgg tatgtgcacc gtgatataaa

9841 aacaactaac atcctacttg atgagggcct cagagctaag gtatctaaat catgatcctg

9901 tataatatca agaaatgctt atccgttact cctatgatca agttagctat ttcaaaccag

9961 ttagttccat aatagtcatc ttccgctaca tgacaaattc tgataaaaat ttgagtagac

10021 aaccatgttt aaatataaca tagctctagt tttaaaacta tatactaaaa ttgaccacta

10081 cccggcatcc atttcaacag gtaacagatt ttgggttagc aaagcttgtt gaacgagcca

10141 atgaagagga tttcatagcg acacgactag ttggcacacc aggctacctt cctccagagt

10201 aagttcaata atacagtgga ttcaagttct cttcgatatc atttcgatca ttttctcatg

10261 caaacaaaat atactacttt gtttctcaca ttctataaaa atcttttgaa agatcggtga

10321 aggagcttca ggtcaccccg aaaacagatg tatttgcatt tggagtggta ctagctgagc

10381 tgattacagg gcaacgcgca cttatccgtg acaaccgaga gcctaacaag atgaaatctc

10441 taataacagt tgtaagttga aaactataat tccttatgaa aagagaaaca agatgaaaat

10501 cattaacaaa acacattatc ttcttgaaaa gcttttgtcc cagccaataa catgtggttt

10561 cttctaggcc tgcaatttga agctgtcttg tctatcaaag aatccaaaca acttttccct

10621 atcgaatggg attggcttgg ctcttcattg ttatatagga acattacaaa cataaagaaa

10681 agaaaataca aatcaaagtt ctgtattgta tcttcacttg tgaacatgag gcctgcaatg

10741 cacatacatg acatgtctgt gcaatcagga ctgtccatca taataatgcg ttacggctaa

10801 caaaatcctt ttgaacgtaa ctaagaacta aagaagctga cttctgtttt gcaccacatg

10861 atcaggttaa aagaatattc caagatgaag atccagaatc agctttagaa gccgaaatag

10921 acggaaatct ccggggcagc taccctagtg aggatgtctt caaggttggt cacccaactc

10981 tacgagtctc ctttatcttc ctcatttgag acatagattt aacagagaaa agcccattat

11041 tgttatcaaa gttttattat gtactgatct ttatacttga aaaagttcaa aacacaagtt

11101 ttcaaggacc ccatttatag aactaagaat ttaacagtgc ccaactcaaa aaattaccta

11161 gtgggacttt gttttgtata taaataaaga tagtcttatt cttgatgttg ttggtactat

11221 ttggcagatg gcagaagttg cggagttgtg cttacgtgaa gaagcagtgg acagaccaga

11281 gatgagggat attgtggtga cactgtctca aatagtgatg tcctcaatag agtgggaagc

11341 atcacttgga gggaacggcc aggtcttcag tggggtattc actggcagat gaacatattt

11401 tttctcagat gtatactatt ttttcaataa taccttacta

//

LOCUS Panepr_flanks_(reversed) 11404 bp DNA linear UNA 26-JUL-2021

DEFINITION .

ACCESSION .

VERSION .

KEYWORDS .

SOURCE

ORGANISM Parasponia andersonii

FEATURES Location/Qualifiers

Promoter join(1..>1677,<7363..7516)

/created_by="User"

/modified_by="User"

/label="Putative promoter"

Polymorphism 1678..7362

/created_by="User"

/modified_by="User"

/label="Retrotransposon"

CDS join(7517..8165,8417..8620,8783..8913,8983..9072,

9162..9267,9641..9792,10013..10110,10225..10363,

10778..10876,11140..11304)

/created_by="User"

/label="CDS"

gene 7517..11304

/Source="merged"

/Pseudogene="True"

/Productname="LysM receptor kinase-like pseudogene"

/Created_by="User"

/putative_orthologs="None"

/ID="PanWU01x14_263630"

/modified_by="User"

/label="Panepr"

ORIGIN

1 attattagtt taagttctaa atatagtttt ttctagattt tgttatgacc agtaattgaa

61 gaagaggtca ataagagcaa agacttctgg ggttgcgaac agaaatgaaa tattgcaata

121 ttgtttgatc ttggtccgaa agacaaggtc gagaccggag gttgccttaa tgtcgtgtgt

181 catttcctag tttgtccggg agaaaagagt cccaaaaagc cactaaattg tcgttccatt

241 acttaatttg gtcaatggtt tggttcaaat atcttaagtg gcgaatttgg attgtactta

301 caaatctacg ttctccatta gataagttgc tccatcttca aattaatatg tttaaaaaga

361 tactattaaa taattctgcg gtagaccaaa agtccaaagt gagatgatca ttatccgttt

421 ttggatccat tgccaagata tggagctcac aagtcaaaag tcaatctgaa tcataggtgt

481 gcaactggaa ccgccaatcc aaaagtttcc tacatggaaa gaccaatgga ccaggcctga

541 caaactgatg ggttggactt aattggactc ttttcattcg taggaactgg gcctgctata

601 atgtagtagg cctcctgttg gacttgtctt tctaagtaga tcctcctcca aaatgcataa

661 tccaacttgt cccggtcctg aaatggcaaa atgcatgtgg tttcctttag ccggcaaaga

721 atatctatgc atagttgcat acaataagaa aagactagtc agcacttgtt tctgactccc

781 taattctaat aataatttaa ttaagtttca ttacctcttt gttgattcag gagggaagtt

841 tgtttttcac ttggttgaca atattttgga ccgctgcttg gattcaataa tagactcaag

901 tacagcattt agattgcttt gcaatgaaaa gagaaaataa aacaggagag agggtgaaat

961 cagtgaaacc aagtgagaaa aaaagggctt tcccaccaag gagaaaacca cattcgtctt

1021 attcattgac atagaatgaa ttaaaaagac agaacaaact tagcccttcg tagagttgaa

1081 caggccctat tatttggggt ttgacttaaa gaaaatcatg ttcttgtcca cataacacaa

1141 atttccgaaa atggacttag gaatagccaa ccatgctagc agggcccttg taattaatgt

1201 tactaccaaa attggcttac atgacaatta tcacaatcat ttatttttgt agacccataa

1261 tgctgaaata tcagctatgg atgattgtga gcatagttca ttttacacta agaccgcttt

1321 agatgaatta ttgggttact tatatgtcca caaaattgta agtaaaactt acacttacgt

1381 cttatgagca ttgtattgga gcatgtaaca ctgcccgtca aagtaaagct gtatgtcgtg

1441 tcagcgttga actctgtttc atttagattg agaaatttta tgggcatttg actttagctt

1501 ttgtctgata atacgacccg gccattcctg atatcatgaa caatccgttt gatgttatca

1561 gatgaaaaag ttgtaataat cgatgagatg ttctttatat aaaaatccca tctgaaaaca

1621 gatttatgta taaataacaa taccttgtta ctctctttgt ctgatattat acaactcttt

1681 ctttttcacg caacgaattg ttaaatttcg ctctaacttg tgttttcaca atgagaagaa

1741 aaacgagtaa aattctcaaa gagaatttag aaaagaaaac taagatgtga ttgaagatta

1801 gatgattaca tctcttgtca atgaaagtta tttatacaag tttaagatta atctaaacca

1861 tctagtaaaa taattctaag aaacttacaa ccatatacct agtacctacc aaccaaacac

1921 acttaaataa ataaattgtt cacctagaat actttaaata aacaaattgt tcacataata

1981 cacgtcaaaa aaataataaa caagctaaat tacctaatac attttaaata aataagtcta

2041 taatacctaa cacacaaaag tattctacca accaatgaaa tgttcattag attgggtctt

2101 gaaattttgg accttggctt aggtcttata tgtttgtggg ctactgcatc agttctcccc

2161 ctcttgaaaa aaaaaaactt gtcctcgagt tactcaaggt atgactttcg gtccatgatg

2221 ctcgtataga tcttgaacat tgaaggtctt agaaattgtg taactatctg gtagatcaat

2281 gatgtatgtt ttatcattaa ttttttgaat gatgcgagag aggccaattt tcttttgctt

2341 gagtttatta tattcccttg ttggaagtct ttctttacat aaatgaaaca ttataaaatc

2401 gtcctctttg aaagttttga atcttctgtg cttgtcagca ttagccttgt attttgcagt

2461 agactcttct aacttttttt tttacttttt catggatgtg gatcgtctta ttaatcaaat

2521 gatcgacaat aatactcatc cctgaaagtt ttggtaaaga taccaagtcg agagtatgca

2581 acggaggacg aatataaaca atttcaaata aatatttact gcaggaataa tttggcacat

2641 tgttgtaagc aaacttaact tgtggtagga tattccctat tgtctagggc tttctttgat

2701 caagcaccgt aaaatattcc ccagtgtgtg attagtgact ttagtttgct tatctatttg

2761 gggatgacaa gtgctactaa attggaaggt gatatcaagt ctcgtccata aagtatacca

2821 aaaatgactg aggaatttac tatcgcgatc agagactatt gtccttggta acccatgcag

2881 acgaacaatc tctcgaaaga acaattgagc aatataggta gcatctgaag ttttcttgca

2941 gggaataaaa taagccattt ttgaaaaacg atttaccaca acaaagatag aatctgaacc

3001 ttttgtgttc gcagtagtcc taagatgaaa tccatgaaca aatcttccca aggtgtatca

3061 agaataaaaa gggacatgta taacatagtg ttttgaactt gtccctttgt agtctggcat

3121 atataacaat atttgacaaa tttctcaaca tcatgcctaa gatgtggcca aaagtattta

3181 gcttccacta atgagatagt tttttctcgt ctcatgtgtc ctcctaaccc tcctgcatat

3241 agctcctgaa tgatgtgttc tctgagagat cctcgtagaa tacataaacg gatttgaaaa

3301 tatagaaaac catcatgtat ttgatgtttt ttggcaactc cactaatgca cttctcccat

3361 tcttccttga aatcttcact attagtgtat aagtctttta aataatcaaa attgattatc

3421 tcattttgaa caacagtcaa taatataact cgatgactca atacattagc aacattgttt

3481 aattatccta acttatgcct gagagaaaaa gtgaactttt ggatataggc aatccatcga

3541 gcatgcattc gatttacttt ggaagagttg ttgaggtact taagagcttg atgatctgta

3601 ttgaggatga atttcttctg tactagatat gactcttgtt gttgtaatgt acgaataacc

3661 gtatataatt cttgttcgta cgttgaccac ttctttctgg cttcactaag cttttcacta

3721 aaaaaaacta tcggcttcat atcttgagat agaacagctc taactcttgt gccagaagca

3781 tcacagtcta cttcgaaaat tttattgaag tcataaagtg cacggatggg tgtatttgta

3841 agtttttact tcaagatagc aaaactctgt tccacttctt ctgtccaaaa gaattgcttt

3901 tgcttcaaac attcggtgag tggtgcggct atactactaa agtttctcac aaatcatgta

3961 tcagaacgtt gcaagacata aaaactccga atttctataa tggtcttcag tattagctat

4021 tctcggatag ctcttaccta attttgatca attttgattc catcagaact tacaataaag

4081 cctaaaaaga ctaagctatc agtcatgaag ctacattttt caggttgaca tacaactaat

4141 tcttataaag tgtattaaaa acttcggtca agtgttgtaa atgttctttt tgattctgat

4201 tatagaccaa aatatcatca aaataaacga cgacaaatct tccaatgaag gggttgaaat

4261 acctgattca ttaactttat gaaagtactc gggacattgg atagaccgaa tgacataagt

4321 aaccacttgt atagtctttc atgtattttg aatgtagtct tccattcatc tctaggatga

4381 atacgaatct gatggtatcc actatgcaaa tcaattttgg aaaagagctt taatcctccc

4441 aatctatcaa gtatgtcatc caatcgtgga atagaaaatc gatatcgtat cgtaatcttg

4501 ttgatggcct gactatcaat gcacatcctc taactttcgt ctttcttcag tattaggagt

4561 gcaggtacaa ccatggaact catactttct ctaatcatcc ctttcttgag gaggttttcg

4621 acttatattt gtaggatctc atactcctta agacttatgt ggtagtacag tagacttgct

4681 ctaggaataa gatcaatcta gtattgaata tttcataggg gtggtaagct agcaggtaac

4741 tcctcaggaa cgatgtcttc aaattcagac aataatattt gaacttacac aggaatatta

4801 gtaggagtat atactttttt aggtacaaaa agaaggttgt ttggttattt tgcagcttca

4861 taggagctag aataattttt atattatcct tatagaagct gtttgtgtta gcttttccat

4921 tatgcatggt gtttaaatcg aattgccatg gttaaccaag caacatatga caagtatcta

4981 tatcaaaaca tcacataata cctcatcttt gtatttatcg atggagaaag gaatttgaca

5041 ttgtgatgtc acctttattt cactgacgtc tttgatctag ccaatgcgat acatggcagt

5101 atatggttgg gttggtattt gtaacttttc catcatagtc ttgaatacaa tgttctcact

5161 actcctatta tcaatgagaa gatcacaaac tcattgttca accatataat gagtctcgaa

5221 gatgttgtat cattgcatgt cttcttcctt ctttagtgta tacatcaatc gtcttatcat

5281 caaggaaata tcttcatctc cataaataat ttcttcaggt tcaacttcat ctacgtactc

5341 ctcattcttg aaatacgcct cgtccccttc gacatcatca tcatgtgtgg ttagattaat

5401 aggtttattc tactaacatt gatttaaata gtagcttgtt tgtccacaac gataatactt

5461 attacctctc aggactgcat aagggttgtt agtgttgtcg ttagttattt ccctttgtag

5521 tcgagcagtt ggttgattgt gagaaggagt tggtcttact aattcatcgt gtggtcttgc

5581 tatggccttt cctttattca aatcagattt ttatggtggt gtagaagatt gagagattgt

5641 tgaattacga tttgctcctt tgttgagctg agtttcaatt ttcaaagtga gttgaattac

5701 actagacaaa gtattatgag attatttgaa tacctcgtct tatatgtttg cacgaagtcc

5761 atcaatgagt ctagaaatct tttaactttc agattttcga atatccactc gggtagttag

5821 tctatgaaac tcggttatgt attcttgtac tatctagtat gcttgttggc agtattgata

5881 tcgactaaaa agtgcttgtt tgtaatttgg aggtagaagt gctcgatcat caattgtttc

5941 atccgtcgcc aagtgtaaac tttgtttctc ccttaccgaa tacgtgtcgc ttttaattga

6001 tcccaccaag ttaacgcacc tcctttgagt cgataagcca ctaatttgac tttcaagtat

6061 tctggagtgc cctaataatc aaagaacttt tctacttcag ctagctaatt aaggaattct

6121 tcaatcacga atagatcatt aaattacaga atctcgatct tcatgcgata gttattttgc

6181 cgaagtcctt gattgtttct aagcgtagct ggttcttcat ggtcttcact agaatcttta

6241 tcttgaaaaa attgatcttg atatgcctaa aatttccaaa aggatcaact tcttcatgtc

6301 tctgtcgttc ataattgttg tttcgtttcc caagaaagtg ttgagtcaaa tttgtaagca

6361 ttgtctctaa attctgcaat cttctatcaa gtgcatccac tgactcttct attgcaaact

6421 cttctacaac gggattttgt tggctagact ctccatccga tatttttttt taatattttt

6481 ttaccttttt tttttttttt tgcttttata ggaaagaaga tagtataaac ttttaaaagt

6541 aaagaaaaat ataattactt ttttttactc tttttttttt ttagagaaaa gaaaatagta

6601 caaactctta aaagtaaaga aaaataaaac tctcaaaaat ttaaacagta aaaaaaaaat

6661 taaagattat aaaaatgtat atattacctt gttaaaggaa tgaagcaaat tctctaagga

6721 ggaaatcttg atatacactt cttgtatttt ttttttttca gaaacaaaga tagtgtatga

6781 aaggattata tgtgtatgtg tggtaaataa ttattatagg aaggtgatgg taaagattag

6841 atagcgaagg tgatgatgaa ataaggattt tttttttatg aaaataatgt acagtgacgg

6901 gaataggaaa gctctgtctg atacagaggt agtctctgat accaattgat gcagcggatt

6961 actagatttt gctctaactt gcgttctcac aacgagaaga gaaacaaata aagttctcaa

7021 agagaattta aaagagaaaa ctaagatgtg attaaagatt ggatgattac aactcttgcc

7081 aatgaaggct atttatacaa gtttaggatt aatctaaact atccaataaa ataattctaa

7141 gaaacttaca atcatatacc tgatatctac caactaaaca tacttaaata aataaattgt

7201 tcaccttgaa tactttaaat aaacaaattg ttcacataac acatttcaaa taaataagct

7261 aaagaaccta atacatttta aataaataag tctataatac ctaatacaca aaaatattct

7321 actaaccaat aaaatagatt gagttttaga attttgggca tttgctttgg tttattgaaa

7381 aaacaaacaa accagttatg acaaagagaa caaagtatgg tgtgtaactc caacattgcc

7441 aaactttgtt gtgtaacgcc aacgttgcca ttacttcttt ctgttcttca aagagaaaaa

7501 tccaattcca aaaaccatgg caacccatca tctccttccc tatcttctcc ttatgttccc

7561 tctttgttct cgagttttta cactccatgt gtctatgaaa gaatctctta tgtacccttt

7621 taactgctcg gcacaaatca aggcgtgtaa tgcttcattg taccatatca atgaaggtct

7681 cacaataaaa gaaattgctg cttattacaa ggtcaacgca tcctcccaaa ttgagtccat

7741 aatgcataac aacagggaag attacctcat aacagtacct tgttcctgct catccatgtt

7801 tggcgtaacc ggatattttt acaacacgac ctacaacgtc aaattaaatg acacttttct

7861 agatgtttca gctaagtact atagtggaca agcttggaga tttgaagaag aagtccaata

7921 tttcaagcct gataaaaatt ttaccatgca tcttctttgt ggttgtctag aaagtgactc

7981 cggaattgtg gtaacctaca cagttcagga ccatgataca ctataaaata ttgcaaatct

8041 gttgtcagcc aaattcgaca acttggtgag attgaatgga tatttgactc aaaaccctgc

8101 tttcattgag gtaggctggg tcttgtttgt gcccaaggag aaaaatggaa ttaaaacatc

8161 aacgggtgag ctctgtttga tatatggaac aaatcattgg cttgaaaatt gaaaattgct

8221 tagccaaaac acacatacat gcgcgcgcac gtctatatgt atgtatatct gttttgagta

8281 agaatgcata agaaatgaaa tacatctata cgtgatatac ttgcatgcag gaatcccaga

8341 atttagtaca cgataattga tgatcctttg taggaagttc actatatatg ttgttgttta

8401 ttttctttgc catcaggtac gagagagaag tggaaaattg taactggtat attgttaatt

8461 gtgacgctgc tttcaactgg tgcattgatg gtcattcttc tcagaagaaa aagattgcag

8521 caaaagaaag tggaagatcc tcaagtccta tcaaaaattg tgactactaa aaaatctctc

8581 tccttgcaga atcctttcct tcctaaagaa aattttgaag gtgagaccga aaaaaaaaac

8641 acttgcttat gtccaaaact agttttattt atgtattgat ctatgcttaa tatgcctttt

8701 attagctatg actagaagac tttctggact aagagagatg attacggtta tactaatcca

8761 tgaaattgta tatatgtggc agattttcat tcagaaaaaa ctgtagtatt tagtcttgag

8821 gcgatcgaag aggccactgg acactttgac gaaagtaaga aagtaggaga ggggggatat

8881 gggtgtgtgt attttggcat actaggagag aaggtatgga aagtctcctc tacttatttt

8941 taggttactc atgactgaaa atgctaaaca tttcaacttc aggaggttgc cataaagaag

9001 atgagatcga ataaatcgaa ggagttcttt gcagagctaa aggtcttatg caagatccat

9061 catataaacg tggtgagtaa acaaataaat tctatacaag ctcttttcac tgatatgttg

9121 aaaggaaaag ctctcattca catgtaaaaa tttgtcttta ggtggagctt ttggggtatg

9181 ctagtggaga tgaccacctc tacttggttt acgagtatgt tccgaacgga tcactgagcg

9241 atcatcttca tgatccgtta ttgaaaggta tttcgagtaa ctggtgcaaa tgcttctgag

9301 tttgttggat gaattttctt ggctcaaata cataaaattc aagtacttat ctgtttcttc

9361 atgtgctagc actgaaatat ggaggctttg catatttacc tagagcaaga ttctagtgct

9421 ctttatcaga atacatctta attaacaagt cttagaaggg ctttattcat tcatacattc

9481 ttgttcaatg aatgagatta ttagcacaac attgcaagcc ttcaattcag aaataatttc

9541 tcaagataga acttcaaaac ttcacttgag caagaatcct tgtcttacgc tgtaaagcaa

9601 aaaataagaa attgactaga aaagaatgct attgaatcag gtcaccagcc tctgtcctgg

9661 actgcaagag cacagattgc acttggcgct gcaaagggta ttgagtacat tcatgacctt

9721 acaaaagcac ggtatgtgca ccgtaatata aaaacaacta acatcctact tgatgagggc

9781 ctcagagcta aggtatctaa atcatgatcc tgtataatat caagaagtgc ttatccgtta

9841 ctcctatgat caagttagct atttcaaacc agttagttcc ttaatagtca tcttccgcta

9901 catgacaaat tctgataaaa atttgagtgg acaaccatgt ttaaatataa catagctcta

9961 gttttaaaac tatatactaa aattgaccac tacccggcat ccatttcaac aggtaacaga

10021 ttttgggtta gcagagcttg ttgaacgagc caatgaagag gatttcatag cgacacgact

10081 agttggcaca ccaggctacc ttcctccaga gtaagttcaa taatacagtg gattcaagtt

10141 ctcttcgata tcatttcgat cattttctca tgcaaacaaa atatactact ttgtttctca

10201 cattctataa aaatcttttg aaagatcggt gaaggagctt caggtcaccc cgaaaacaga

10261 tgtatttgca tttggagtgg aactagctga gctgattaca gggcaacgcg cacttatccg

10321 tgacaaccga gagcctaaca agatgaaatc tctaataaca gttgtaagtt gaaaactata

10381 attccttatg aaaagagaaa gaagatgaaa atcattaaca aaacacatta tcttcttgaa

10441 aagcttttgt cccagccaat aacatgtggt ttcttctagg cctgcaattt gaagctgtct

10501 tgtctatcaa agaatccaaa caacttttcc ctatccaatg ggattggctt ggctcttcat

10561 tgttatatag gaacataaca aacgtaaaga aaagaaaata caaatcaaag ttctgtattg

10621 tatcttcact tgtgaacatg aggcctgcaa tgcacataca tgacatgtct gtgcaatcag

10681 gactgtccat cataataatg cgttacggct aacaaaatcc ttttgaacgt aactaagaac

10741 taaagaagct gacttctgtt ttgcaccaca tgatcaggtt aaaagaatat tccaagatga

10801 agatccagaa tcagctttag aagccgaaat agacggaaat ctccggggca gctaccctag

10861 tgaggatgtc ttcaaggttg gtcacccaac tctacgagtc tcctttatct tcctcatttg

10921 agacatagat ttaacagaga aaagcccatt attgttatca aagttttatt atgtactgat

10981 ctttatactt gaaaaagttc aaaacacaag ttttcaagga ccccatttat agaactaaga

11041 atttaacagt gcccaactca aaaaattacc tagtgggact ttgttttgta tataaataaa

11101 gatagtctta ttcttgatgt tgttggtact atttggcaga tgacagaagt tgcggagttg

11161 tgcttacgtg aagaagcagt ggacagacca gagatgaggg atattgtggt gacactgtct

11221 caaatagtga tgtcctcaat agagtgggaa gcatcacttg gagggaacgg ccaggtcttc

11281 agtggggtat tcactggcag atgaacatat tttatctcag atgtatacta ttttttcaat

11341 aataccttac tatatataca tgtacatata tgcatatata cacaaagctt taaacaataa

11401 caac

//

LOCUS LjEPR3_flanks_(reversed) 8300 bp DNA linear UNA 26-JUL-2021

DEFINITION Chr2.

ACCESSION .

VERSION .

KEYWORDS .

SOURCE

ORGANISM .

FEATURES Location/Qualifiers

Promoter 1876..3430

/created_by="User"

/modified_by="User"

/label="Putative promoter"

CDS join(3431..4100,5003..5206,5320..5459,5571..5660,

5751..5856,6803..6954,7251..7348,7500..7638,7837..7935,

8036..8200)

/created_by="User"

/label="CDS"

gene 3431..8200

/Source="protein_coding"

/ID="Lj2g3v1415410.1"

/Type="protein_coding"

/modified_by="User"

/label="LjEPR3"

ORIGIN

1 tgttcctcca tcacatgatt acatatagaa tttgctcata gaatttatgt cattgaatta

61 ttgatacttg agataggaaa agatcatcat tatctctatc agtacatcaa taaaacaaaa

121 agaataacaa atttagtcaa acatgatcat catcaaggcg aggaggggtg gcgaagacac

181 atgttaattg gttgccctgc aaatcttcct cacctctcca tcattccctg tcaacacgtt

241 caaggaagtc aacttagtca tcacctcagc aaatctcttg tggaacaatg cctgatcatc

301 agccatttgc cccacagtag gtgcagtcct tggatcagtc accaaatgcg catcagttag

361 gagcaatgat ttccccttgt caaccaagtc cctgtagaag aggttgtcca tcaccgtcgg

421 cgtgttgtca aagttcaccg gctcgttcct gtaccgcggt gtcccggggt tggaacaaat

481 ctgcctgagt tcattaagga agggtggtcg cagagcaggg tctggtttgt tagtgttctt

541 gaagttatac accctgtcca tgaaaatatc acagtgagct gcaccaatgg aatgtgctcc

601 caacaagatc accatttcct ctgggctgaa ccctttcctc tggaagagct ctaccatctt

661 atctgctgac cagttcggca ttggtaggtt gttgtcatcc actacggtcg cgagggagta

721 gagcgagtcc ctgcgaccac cgagcggttt ctggcggggc attcccgcca gtgccatcgc

781 ctcgaagctc aggaacacca tcgtgtcagc gcaggacacg attccagggc attcttcctc

841 taatttgctc ttgatatcat caaccagatc aggacctttc aggagtatac cgttaaaaaa

901 tgaactcttt tccacattgt caccatttgg tgtggagtct agcaagatcg atgcatcaca

961 cccctgtttt gcatcatggc aaaaaaaaaa tcttagtcat ctttgtttag aattgaaaag

1021 tataatgatt cgcagaattc cacacattac agacaaccaa cagacatcca gcattatgtg

1081 aaattttatc atgggaggat attaatttga tttatgtcat atgtgtatgt gttagctatt

1141 acaaaccctt attatgacat gactaattaa gaaacaataa ggatgttttt attattcaag

1201 ttagacctct gaggttttat gggccggcag acgaccacgg tcccaacctt tttagcaccg

1261 gaggtgtact aatcaatgaa cacacccgtg gaaccttttt ttttcttctt acaaaatcaa

1321 ccaagcctaa ccggtcacaa catagaaatg atggtttctg ctcaatcctt tataaggatt

1381 agtttagcca tatctttagt caatgtaaga tttctaatag tttttttttg aaaatgaagc

1441 ttaccacaac gaagcagtcg tggaattgaa gacgaagaat gttggccatt gcctttggat

1501 tggtcctgac aatctcggcg aatgcgtcag cgatgatttt ctcagcattg gggcagctgt

1561 cgctgtagaa tccttccctg agattgggat tgatagtatt gggctggtct tgaaattcct

1621 gtggcttttg aacttcctct ggcttctcct gctgttcatt ctttggtggg tcctgatctt

1681 gaattggtcg aaatgggaat ggaaatccta gaaaattccc gttttgaagt cctaataagc

1741 ccccctcgcc tccttcccga aatggaaaag ctattgaagg aacgaggagc aggttggaga

1801 ggaaaagggt ggtaaggatg atccttgcca ttttcattgt taacaaaaaa aactcagttg

1861 tggggggtca caggagaacc aaagaggaca aagaggtatg agaacctttt ataacttggt

1921 gttgtgtttg ttattattat tatggacaga cagatggttt gatcaatgta gaagctggtt

1981 cttaatatag ttttgttgtc aattcagttc aggtaggttt gagacaaatt ggatttagaa

2041 catgcacatt aagagtttga tggtgtttct attattagtt tgaacctaaa tttgctagtt

2101 gattgttcat ccgttgaaat ggaagttagg ccaacattaa cgagtcaagc agctttctaa

2161 acctgtgttt ttttctccct gtatttttct tgttgtgagt aagagaacaa attgatatgg

2221 gaaattactt gtttgtttct aaagaaagca aaaaacggtg cgcctgcaaa actgtacaat

2281 accagaaaaa caaaataaaa atggtccgtg ttaaaataaa ggtataaatg tcaaacttat

2341 gaatatatga tttccgcaca aactgtggat tgattttttc tcaacttact caacttaata

2401 ctcaacaatt tctccctcaa atgctagtat atctcaagcg aaagtcttta aatagttgca

2461 ttgttattgg gttaactctc taccaacgga acacaccgcc tagtcaccac attgttatga

2521 agacttttaa agagtcatcc tcatagtcaa accaacaatc ggatatggta ctagagttga

2581 gtcaaaagaa ttgattcaaa ctgcacggat ctccacattg gacaaaagaa caaatcacat

2641 ggattaaaca ccacattttt cacttaaaac attaaatata tgagttatct cacttctaaa

2701 cggttcaaaa tttgttattt tcacctacac caaaaaaaca tgaaaacaac atagataaaa

2761 acaattagaa aactttactt tatattttat aagttttagg agtgttagct tcgaacatga

2821 cattttactt tttattctta tgactatgaa tcacacgaat aaaaacatag acaatcagat

2881 tattgccaca ttctttttaa attaataaaa gctattcatt cttcacacca gacttcaagg

2941 aaaaaaaaac ttctcatcac atcactaatt tttggtgcct atgatagtgc aacgtcatag

3001 aagtgtcatt aaactagtta aaatcataat gaataatgat caactccaaa aaaattgcta

3061 gtgtatgctt gaccaatggc accatacaat tgtagttttt tgaagtggtg gaacagtctg

3121 tgacttttat cttcaactgg aggtataatt tttgtcattg ttatagccgt ctgagatccc

3181 acaaaagata ccctgccctt acagacaagt ataagaggca caaaaaaaac tttattctaa

3241 tcatcatcat tcatggactc atcgtaatat gtaaattcgg aacctataag aaaaaggatc

3301 caggtaatta atatcaatta atcttggtga gttaggatgt tcctgtttct tgaaagcagc

3361 tacaaacaac taaatgatgt taagagaagc tacatcatgg ttttgaaact tcctcattgc

3421 attgaagaaa atgttttatg atttcacaac tatggcttct ctaactcatc ctctatgtgt

3481 tctccttact ctaatggctg cagcttcatt tgcaagtgtg ttttcccttg aagtttcatc

3541 caaaacaact tacatggaac cttttaactg ctctacaaag atcagaacat gcaattcctt

3601 gctctaccac ataagcatcg gtctcaaggt cgaagaaata gcccgctttt actcggttaa

3661 tctctcccga atcaagccaa taactcgcgg taccaagcaa gattaccttg tctcagtgcc

3721 ttgtacttgc agaaacacca atggccttaa tggatatttc tatcatacat cctacaaggt

3781 taaggttaat gacagttttg tggatattca gaacctgttc tatagtggac aagcttggcc

3841 tgtgaatgaa gatttggtgg ttccaaatga gactatgaca atacatattc cttgtgggtg

3901 ttcagaaagt ggctctcaaa ttgtcgtcac atacacagtt caaaggaatg atacaccatt

3961 atcaattgct cttttgctaa atgctacggt tgaaggcatg gtgagtgtga actcagttat

4021 ggctccgaat cccacattca tagatgttgg ttgggtgtta tatgttccca aggagttgaa

4081 tccaatttcc catggaaaag gtgagttcta tttcttaacc tgtccccaga tttgaatatg

4141 catggcattc acatatttac aatgttttgg agtacatgtt cctttcccac attcagaagc

4201 aaaatcatgt taattaagtg agactattgt ttttttttta cttatgcctt ggacatgcct

4261 catatcgtgt ttggttctgc gatagaaagc cctagaatta cttattggaa gaaggcacaa

4321 attgtatctt atggagttac cgtgctttga attatgtgac acaaccaatg aacacactgt

4381 cagagtgtgt gccgccacgg aagcaaacaa gcacttgttt agtgaactgc atgatgatcc

4441 aaattcatat acttacaact gcattgagtt cgcattgatt tcagcatgtt ttggactgag

4501 ccaattgcat tgttatggtg catgtttgga tacacggtaa acgagaattg attatgggag

4561 aagctagtca tagttgcttt tgtggtagcg gaattgattt tgaaagatct gaatgtgaat

4621 taaaacatgc acataattcg atttgaaagc tgattcctat agtagaactt ttatctttag

4681 gtaacaacct cagctgttgt taggaagatt ttataaaatt acaaggggtt atctttgatt

4741 gatcaatact tctattttga tgctaccatc tgcccaaaac ctgcatcaca attatggcca

4801 aatatagatg actagtcatt caatcatcta atttcaacat catcagataa atgtttctca

4861 tgcatcaatc atcgaaattg gtaacctttc accaattgct gttcagtgtt catagaccag

4921 gtgaagttta aagtttagat agtttcattt ttgctagcag gcactcttaa tttatcatgc

4981 tttgttttgt tgttgtttgc agaaaataaa cacaagctgg agaaaattat tggcatctta

5041 gcgggtgtga tattactttc aattattacc ttgatcattc ttatcgtcag gagaaataga

5101 tcctatgaaa cctgcaaaga tgatccacgc gctatctcaa aaagatcaat cggcaaaaga

5161 actagttcct taatgaaccg cgactttcac aaagaataca tggaaggtaa caaaagtaat

5221 atttgaaaaa tactaaaatt tcagcattag gttaatagct ctacatgcaa ggaaactgag

5281 aggaagattg ctgcttattc atggaaaata ttgatgcaga tgcgacatca tttgactcag

5341 aaagaccagt aatttatact cttgaggaga ttgaacaagc tactaatgac ttcgatgaaa

5401 ctcgaaggat tggagtcggt ggatatggaa cagtgtattt tggagtgtta ggggagaagg

5461 tatggattat ggaaatccca cctacattct ttaatggtcc tccccatgct agcatttctt

5521 tttcatgctg caacaaagca ttgaagaata atagctattt tgattttcag gaggttgcta

5581 taaagaagat gaaatctaac aaatccaaag aattctatgc agaactcaag gccttgtgta

5641 agatccatca cattaacatt gtaagtgaca tctctgatga agttattttg gacagaggct

5701 tgatcatctc attttgtttg attatattat agatgtaatc actgtttcag gtggagttat

5761 taggatatgc cagcggagat gaccaccttt acttggtgta tgagtatgtg cccaatggat

5821 ctctcagtga acatcttcat gatcccttac tgaaaggtaa attggctcta agatctattt

5881 gcctgcaaaa tgcaaacctt ctggctttta gaaatgttct aaaatgccat aaaggctaca

5941 agaccagacc tccaaacaaa gcatatcata gatgatagtt tttatggtaa ttgcagcctc

6001 atatattgat tttttaactc atgaaagaat gaaaaataga ttattttctg aaacactagg

6061 aagcatgtta tatagaattg acatcaattg ttgtttatac ttaataagac cattgaacga

6121 aagttcgatt caacatgcca ctaatgtcgt tgtactacat ttctgtaagt aaaagaaaaa

6181 ggcttaattg caactttggt ccctgacgtt taccaattcc acgattttag tcccccacct

6241 attttaatta cacagatggt ccctgacttt gctggcagtc tgcaacgttg gtcctaccgt

6301 ttgtttgtta atggaggagg tttacgtgga tggtcactta gctgatgtgg aagttgaatt

6361 agagagagaa agagaccagc aactgattca gccttcttcc tcgcctctag ggactcgttc

6421 gttctttatc tcttcctttt cgctaaatct caagcttcga aacatcttcc tcacggtgct

6481 ctgtttttta aggaggaaga gggtagaaga tgaccgttgc aggtctcacg tgcaactcac

6541 gtgtttccct ctctcttcag tccttctttc tctctccaat tggacatcca cgtaagcctc

6601 ctccgttaac aaataaacgg caggaccaac attgcagacg ggcagcaaag tcagggacca

6661 tccgtgtaat taaattaggc gggggactaa aattgtggaa ttggtaaacg tcagggacca

6721 aagttgcaat taagccaaag aaaaaaaact aacatttctc tcatgtttat gtcaagaaat

6781 ctaaattgtg gtactggaac aggtcaccag cctctttctt ggtgtgctag gattcaaatt

6841 gcactggatt cagcaaaagg tattgaatac atacatgatt acacaaaagc acagtatgtg

6901 caccgcgata taaagactag taatattctt cttgatgaga agctcagagc aaaggtattt

6961 ttccttgatt tcttagtctg tcctgatttt ttttagtttt atgaactagt tatcattata

7021 catatctaag gcatgctaac ctgcaagaaa caactatgta agtcagttgc ctacttgcct

7081 aatgataaca agaaaagatg gtcttaaaac attatactag taaaatcaag ttaatacact

7141 tgctggacat gaatttattt gatttattag taaaatcaag ttaagcaagt aatgttgaat

7201 cgaggtcttt ctacttcttt tcataccaaa gcatttgatt tcatatgtag gtagcagatt

7261 ttgggcttgc aaagctagta gaacgaacca atgatgaaga attcatagca acaaggcttg

7321 ttggaacacc aggctatctt ccaccagagt aggtttcatc cttttcaatt ataactagat

7381 cttggtgcat tcaacagaaa caaatttcct cactgccatt aatcttctta aactcaagca

7441 aaataaatta cttcacagtt aatattgctt ctgatatcat tacttttttt cccttttaga

7501 tctctaaagg agcttcaagt gacagtaaaa actgatgtat ttgcatttgg ggtggttatg

7561 ttagagttga taacagggaa acgtgcacta tttcgtgaca accaagaagc caacaatatg

7621 agatcacttg ttgcagttgt aagttaactc ttgtttgaga gaaaagaaaa tatagatggg

7681 gcttgaaagt attatataag acatccaaaa agtcattcac caaaagtgaa gtttactttt

7741 attcacatag atctaactct gcagtttact caatactctt acacatcttt tgtaattaaa

7801 ggaaagaaat ctgatctttc ttttcatctc attcaggtta accaaatatt tcaagaagat

7861 aaccctgaga ctgctttaga agttaccgtg gatgggaatc tacaacgtag ctatcctatg

7921 gaagatgtct acaatgtaag attcaatcta catcttctat tttgccgata atgttggttt

7981 tctaattata tatctatgtg ccaatgtatg cttcattttg atatgaatac tgcagatggc

8041 agaactatca cactggtgct tgcgcgaaaa tccggtggac aggcctgaaa tgagtgagat

8101 cgttgtgaaa ttgtcaaaga ttataatgtc ctcgatagag tgggaagcat cacttggcgg

8161 agacagccaa gtcttcagcg gggtatttga tggaagatga atgaagacta ctctttacta

8221 attaagaatt gtctattaag agccttctct tgctgcctta atatcttaat cttttgtatt

8281 taatccaatc cttgttcaaa

//
