# Supplementary material for: Pseudogenization of the rhizobium-responsive EXOPOLYSACCHARIDE RECEPTOR in Parasponia is a rare event in nodulating plants
Source: BMC Plant Biol. 2022 Apr 30;22:225. doi: 10.1186/s12870-022-03606-9 (PMC9055685; doi:10.1186/s12870-022-03606-9)
Supplement: Supplementary file 8 — Additional file 8: Supplemental data file 2. CDS of Trema tomentosa EPRa and EPRb. [file 12870_2022_3606_MOESM8_ESM.docx]

**Supplemental data file 2: CDS of *Trema tomentosa TtoEPRa* and *TtoEPRb*.**

>TtoEPRa jg12135.t1

ATGCCAAAAACCATGGCAACCCATCATCTCCTTCCCTATCTTCTCCTTATGTTCCCTCTTTGTTCTCGAGTTTTTACACACCATGTGTCTATGAAAGAATCCCTTATGTACCCTTTTAACTGCTCAGCACAAATCAAGACGTGTAATGCTTCATTGTACCATATCAATGAAGGTCTCACAATAGAAGAAATTGCTGCTTATTACAAGGTCAACGCATCCTCCCAAATTGAGCCCATAATGCATGACAACAGGGAAGATTACCTCATAACAGTACCTTGTTCCTGCTCATCCATGTTTGGCGTAACCGGATATTTTTACAACACGACCTACAACGTCAAATTAAATGACACTTTTCTAGATGTTTCAGCTAAATACTATAGTGGACAAGCTTGGAGATTTGAAGAAGAAGACCAATATTTCAAGCCTGATAAAAATTTTACCATGCATCTTCTTTGTGGTTGTCTAGAAAGTGACTCCGAAATTGTGGTAACCTACACAGTTCAGGACCATGATACACTATTAAATATTGCAACTCTGTTGTCAGCCAATTTCGACAACTTGGTGAGATTGAATGGACATTTGACTCAAAACCCTGCTTTCATTGTGGTAGGCTGGGTCTTGTTTGTGCCCAAGGAGAAAAATGGAATTAAAACATCAACGGGTATGAGAGAGAAGTGGAAAATTGTAACTGGTATATTGTTAATTGTSACGCTGCTTTCAACTGGTGCATTGATGGTCATTCTTCTCAGAAGAAAACGATTGCAGCAAAAGAAAGTGGAAGATCCTAAAGTCCTATCCAAAATTGTGACTATTAAAAAATCTCTCTCCTTGCAGAATCCTTTCCTTCCTAAAGAAAATTTTGAAGATTTGGATTCTGAAAAAACTGTAGTATTTAATCTTGAGGCGATCGAAGAGGCCACTGGACACTTTGACGAAAGTAAGAAAGTAGGAGAGGGGGGATATGGGTGTGTGTATTTTGGCATACTAGGAGAGAAGGAGGTTGCCATAAAAAAGATGAGATCGAATAAATCGAAGGAGTTCTTTGCAGAGCTAAAGGTCTTATGCAAGATCCATCATATAAACGTGGTGGAGCTTTTGGGGTATGCTAGTGGAGATGACCACCTCTACTTGGTTTACGAGTATGTTCCGAACGGATCACTGAGCGATCATCTTCAAGATCCGTTACTGAAAGGTCACCAGCCTCTGTCCTGGACTGCAAGAGCACAGATTGCACTTGGCGCTGCAAAGGGTATTGAGTACATTCATGACCATACAAAAGCACGGTATGTGCACCGTGACATAAAAACAACTAACATCCTACTTGATGAGGGCCTCAGAGCTAAGGTAGCAGATTTTGGGTTAGCAAAGCTTGTTGAACGAGCCAATGAAGATGATTTCATAGCGACACGACTAGTTGGCACACCAGGCTACCTTCCTCCAGAATCGGTGAAGGAGCTCCAGGTAACCCCGAAAACAGATGTATTTGCATTTGGAGTGGTACTAGCAGAGCTGATTACAGGACAACGCGCACTTATCCGTGACAACCGAGAGCCTAACAAGATGAAATCTCTAGTAACAGTTGTTAAGAGAATATTCCAAGATGAAGATCCAGAATCAGCTTTAGAAGCCGAAATAGACGGAAATCTCCGGGGCAGCTACCCTAGTGAGGATGTCTTCAAGATGGCAGAAGTTGCGGAGTTGTGCTTACGTGAAGAAGCAGTGGACAGACCAGAGATGAGGGATATTGTGGCGACACTGTCTCAAATAGTGATGTCCTCAATAGAGTGGGAAGCATCACTTGGAGGGAACAGCCAGGAGTATATGTTGAGAGCGGAGTTTCGCCTKCCTACTATAACAGCAGAAGATGGTGCTCCTGAGAGAAAGGCTCCTATACGCGTCAAGTTTGAGATTCCATATTTTACTGTCTCTGGAATTCAGGTTCGCTATCTGAAGATTATTGAGAAAAGTGGATACCAAGCTCTTCCATGGGTGAGATATATAACAATGGCTGGCGAGTACGAACTTCGACTTATATGA

>TtoEPRb jg14286.t1 (partial)

ATGCAGGTCCAGAAGTTTGCTTTTCGATTTTCCACGATTTGTGAAACAGAGGCGTTCCTGAATGCTGTTAAGGAGATATTGAACGGTGTTAATAACATTGAACCTCTTAGTAGTGAGTTTGGATCGGCAATTTCATCACAATCTGAATTCGTGTCTTCTGATAGACCCCCATATCGAGCTACAGAGGACTTGAGAATCATGAGTCCTGCTCTGACTTATACTCCTGAATTGCCGGCAAGCTCAGACGTCGAAGCAGCGCAACAGTCTTGTATCCAGGAAACCGCACCTACTCACAACTTTGGTACCAACTTTGCAGCTTTTCCTCCCAGCTTCACCTCATTGCTGACCAGCAATTGTCCCATTGTTGAACAAGCTGCAGCAAATCCAACTGTATCTGAGGAAAATTTATTCAAGTCGCAACTCACGGAGGTGGCCATAAAGAAGATGAGATCGAATAAATCGAAGGAGTTCTTTGTAGAGCTAAAGGTCTTATGCAAGATCCATCATGTAAACGTGGTGGAGCTTTTGGGGTATGCTAGTGGAGATGACCACCTCTACTTGGTTTACGAGTATGTTCCAAACGGATCACTGAGCGATCATCTTCATGATCCGTTACTGAAAGGTCACCAGCCTCTGTCCTGGACTGCAAGAGCACAGATTGCACTTGGCGCTGCAAAGGGTAGTGAGTACATTCATGACCATTCAAAAGCACGGTATGTGCACCGTGATATAAAAACAACTAACATCCTACTTGATGAGGGCCTCAGAGCTAAGGTTAAGAGAATATTCCAAGATGAAGATCCAGAATCAGCTTTAGAAGCCGAAATAGACGGAAATCTCCGGGGCAGCTACCCTATTGAGGATGTCTTCAAGATGGCAGAAGTTGCGGAGTTGTGCTTACGTGAAGAAGCAGTGGATAGACCAGAGATGAGGGATATTGTGGTGACACTGTCTCAAATAGTGATGTCCTCAAGAGAGTGGCAAGCATCACTTGGAGGGAACAGCCAGGTCTTCAGTGGGGTATTCACTGGCAGATGA
