# Supplementary material for: Pseudogenization of the rhizobium-responsive EXOPOLYSACCHARIDE RECEPTOR in Parasponia is a rare event in nodulating plants
Source: BMC Plant Biol. 2022 Apr 30;22:225. doi: 10.1186/s12870-022-03606-9 (PMC9055685; doi:10.1186/s12870-022-03606-9)
Supplement: Supplementary file 9 — Additional file 9: Supplemental data file 3. EPR protein alignment of nodulating plants. [file 12870_2022_3606_MOESM9_ESM.docx]

**Supplemental data file 3: EPR protein alignment of nodulating plants.**

>Casuarina_equisetifolia_EPR3_CDS

---------------------------------------------------------------------------------------------------ATGGCTT---CCCATAGTCTTCTTCCCTATCTCCTCCCTCTATTTG---CATC-------TCTATT-----------------------------------GTCCGTCAAAG---CAAGCGTTATTTACCCTCTTGGTTGCTCTTCACAA---GT-CAAGACATGC--AATGCGTTGATCTACCACATCAA---CAACAGTCTATC------GGTAGAGGAAATTGCCT---------------TCTTTTACTCTGTCCAGATA---TCCCAAGTGAAGCCCATACTGCAT---------GGCTACAAACA---AGATTACCTTATTAATGTACCTTGTTCTTGCACAAACGCAGATGGC------GCTACTGGACTTTTCTACAAGACATCCTACCAT---GTGGAACAAGGTGATACATTTGATAGTGTTTCCTCTAACATATACAGCGAACTGGCTTGGAGTAATGGA---GGAAATGGTTCAAAACAAATTCGAGATCCAGATATACCTATTTATCTTCCCTGTGGATGCGTAGA---------------GGACACCTCTCAAACTGTGG-TGACATACACGGTTC-AGGCCCATGA-TACCTTATCAGGAATTGCCCTATTGCTATCTGCTGAACAAAGTGACATACAGAGATTGAACAGAAACTTTACGAAAGACCCATCGTTTATAGAACCGGGTTGGGTGTTGTTTGTGCCGAT---GGATAACAA------------------------------------------AGGAAAGACAAAGAA---ATGG---AAGATAATCATAGGCACGTTATCGGTGT---GGACATTACTTTCGGTAATCATATTGATAGTTGCTC------TACGCTGGAGAAAACGA--TCCCAGCCGAA-------CGTAGGAGATCCA---AAATCTGTTGCGAAAAGCACAAGCTCCAAAAA---------------------------AAC---CTTTGGGTACCGGAGTCAG-TACC-TGGAGGGGGACAACATGGA---------AGGATTTGAATCAGAGAGACCAGTAATATTTACTTTTAAGGAGATTGAAGAAGCAACGAATTGCTTTGATGAAACTAGGAAAATTGGAGTAGGTGGTTACGGGAGTGTGTACTTTGCAATACTACGAAAGCAGGAGGTTGCAATAAAGCAGATGAAATCTAGTAAATCCAAGGAGTTCTTTGCAGAGCTAAAGATTTTATGCAAGATACATCACATTAATGTGGTAGAGCTCTTGGGATACGCCAGCGGAGATGACAACCTCTGCTTAGTCTATGAGTATGTTCGGAATGGACCACTTAGTTCTCATCTCCACGATCCACTGCTAAAAGGTCATCAACCTCTCTCTTGGACTGCAAGAACACAGATAGCACTGGATACTGCCAAGGGTATTGAATACATTCATGACCACACAAAAGCTCGGTATGTGCACCGAGACATAAAGACAAGCAACATTCTACTCGATGATGGGTTCAGAGCAAAGGTAGCAGATTTTGGATTGGCAAAACTAGTTGGGCAAACCAGTGACGAAGATTT---CTTAGTGACACGGCTAGTTGGAACGCCAGGATACCTTCCTCCCGAATCCTTGAAGGAGCTCCAGGTGACCACTAAAACTGATGTCTTCGCGTTTGGTGTGGTTCTGGCAGAACTGATAACAGGGAAACGTGCTCTTGTTATTGACGACGGGGA-------GCC---CAACAAGATGAAATCATTAATTAATGTAATTAAAAGCATATTCCAAGACG---AATACCCGGAGGTTGCTTTGG------AACTTGTAATAGATGGAAATCTTTATCGCAGCTACCCTTTGGAGGACATCTACAAGATGGCAGAACTTGCTGACTGGTGTTTGAGTGAAAACCCAATTGACAGACCAGAAATGCGGCATATAGTTGTGTCACTCTCTCAAATAGTGATGTCCTCGGTAGAGTGGGAAGCATCACTAGGAGGAAACAGCCAAGTTTTCAGTGGG---CTGTTCAATGGAAGATGA----

>Casgla_scaffold955_CDS

---------------------------------------------------------------------------------------------------ATGGCTT---CCCATAGTCTTCTTCCCTATCTCCTCCCTCTATTAG---CATC-------TCTATT-----------------------------------GTCCGTCAAAG---CAAGCGTTATTTACCCTCTTGGTTGCTCTGCACAA---GT-CAAGACATGC--AATGCGTTGATCTACCACATCAA---CAACAGTCTATC------GGTAGAGGAAATTGCCT---------------TCTTTTACTCTGTCCAGATA---TCCCAAATGAAGCCCATACTGCAT---------GGCTACAAACA---AGATTACCTTATCAATGTACCTTGTTCTTGCACAAACGCAGATGGC------GCTACTGGACTTTTCTACAAGACATCCTACCAT---GTGGAACAAGGTGATACATTTGATAGTGTTTCCTCTAACATATACAGCGAACAGGCTTGGAGTAATGGA---GGAAATGGTTCAAAACAAATTCGAGATCCAGATATACCTATTTATCTTCCCTGTGGATGCGTAGA---------------GGACACCTCTCAAACTGTGG-TGACATACACGGTTC-AGGCCCATGA-TACCTTATCAGGAATTGCCCTATTGCTATCTGCTGAACAAAGTGACATTCAGAGATTGAACAGAAACTTTACGAAAGACCCATCGTTTATAGAACCGGGTTGGGTGTTGTTTGTGCCGAT---GGATAACAA------------------------------------------AGGAAAGACAAAGAA---ATGG---AAGATAATCATAGGCACGTTATCGGCGT---GGACATTACTTTCAGTAATCATATTGATAGTTGCTC------TACGCTGGAGAAAACGA--TCCCAGCCGAA-------CGTAGAAGATCCA---AAATCTGTTGGGAAAAGCACAAGCTCCAAAAG---------------------------AAC---CTTTGGGTACCGAAGGGAGGCACT-CATAGTCATATTCCATTGTG----CAACAGGATTTGAATCAGAGAGACCAGTAATATTTACTTTTGAGGAGATTGAAGAAGCAACGAATTGCTTTGATGAAACTAGGAAAATTGGAGTGGGTGGTTATGGGAGTGTGTACTTTGCAATACTACGAAAGCAGGAGGTTGCAATAAAGCAGATGAAATCTAGTAAATCCAAGGAGTTCTTTGCAGAGCTAAAGGTTTTATGCAAGATACATCACATTAATGTGGTAGAGCTCTTGGGATACGCCAGCGGAGATGACAACCTCTGCTTAGTCTATGAGTATGTTCGGAATGGATCACTTAGTTCTCATCTCCACGATCCACTGCTAAAAGGTCATCAACCTCTCTCTTGGACTGCAAGAACACAGATAGCACTGGATACTGCCAAGGGTATTGAATACATTCATGACCACACAAAAGCTCGGTATGTGCACCGAGACATAAAGACAAGCAACATTCTACTCAATGATGGGTTCAGAGCAAAGGTAGCAGATTTTGGATTGGCAAAACTAGTTGGGCAAACCAGTGACGAAGATTT---CTTAGTGACACGGCTAGTTGGAACGCCAGGATACCTTCCTCCAGAATCCTTGAAGGAGCTCCAGGTGACCCCTAAAACTGATGTCTTCGCATTTGGCGTGGTTCTGGCAGAACTGATAACAGGGAAACGTGCCCTTGTTATTGACAACGGGGA-------GCC---CAACAAGATGAAATCATTAATTAATGTAATTAAAAGCATATTCCAAGACG---AATACCCGGAGGTTGCTTTGG------AACTTGTAATAGATGGAAATCTTTATCGCAGCTACCCTTTGGAGGACATCTACAAGATGGCAGAACTTGCTGACTGGTGTTTGAGTGAAAACCCAATTGACAGACCAGAAATGCGGCATGTAGTTGTGTCACTCTCTCAAATAGTGATGTCCTCGGTAGAGTGGGAAGCATCACTAGGAGGAAACAGCCAAGTTTTCAGTGGG---CTGTTCAATGGAAGATGA----

>Alnglu_scaffold25086_cov106_CDS

ATGACAAAGAGAACAGAAGGTACCAATGCTGCAGGCAACTCTTACATTTCAATATTTCTCCTTCATTCCCTTACAAGGAGAACTCCCATGACCACAACTATGGCTT---CCCATTCTCTCCTTCCCCATCTCCTCCCTCTGTTGG---CAAT-------TCTATTT--------GCTAGGATTCTTGCGTCCA---ACACATCCGTCAAAG---CAAGCCTTATTTACCCTTTTAACTGCGATGCTCAA---AT-CAAGACATGT--AATGCCTCGCTGTACCACATGAA---CAACTGGCTATC------GGAAGAACAAATTGCCT---------------CTTTTTACTCTGTCAATTTA---TCCCAGATGGAACCTATAATGCAT---------GGCAACAAACA---AGATTACCTCATCCGTGTACCTTGTTCTTGCACAGATATAAATGGC------ACTAGGGGATATTTCTACAATACATCCTATCCT---ATAAAACAAAATGATTCATTTGATAATGTCTCTGCTGATATGTATAGTGGCCAAGCTTGGAGTGGTGGATCAGGAAATGATTCAAGATTCGACGCAGGAACAAATTTTTCCATTCATCTTCCATGTGGATGTGTAGATCA------------GAGTGACTCTCTAACTGTTG-TAACATACACAGTTC-AGGATCAAGA-TACAGTAGCAGGAATTGCTGCACTACTATCTGCTCAAGAAAGTGACATTCAGAGATTGAACAGACAACTTACTGGAAACCTGGCGTTTATAGCCCCGGGCTGGGTGTTGTTTGTGCCCAT---GGAGAAGAATGGTATTCCGGC---------------------CCCAAAGAAAGGAAGGATATGGAA---ATGG---CCAGTAATCATTGGCACATTATCGGCTG---TGACATTACTTTCAATGAGCACATTGATTATTGTCC------TTCTCAGGAGACAAAAA--AGCCGACAGCA-------AGAGGAAGATCCA---AGAGCTGTAGCCAAAAGCTCGAGTGCCAATAG---------------------------AAT---CTCTCTATTCCAGAGTCAA-TACC-CGAATAAGGATAATATAGAAGATGCAACAGCTTTTGAATCAGAGAGACCAGTAATATTTAGTCTTGAGGAGATTGAAGAGGCGGCAGGTTACTTTGATGAAACTCAGAAAATCGGAGAGGGTGGATATGGGAGGGTGTACTTTGGAATACTGCGGGGGCAGGAGGTTGCAATAAAGCAGATGAGATCTAATAAATCCAAAGAGTTCTTTGCAGAGCTAAAGATCTTATGCAAGATCCATCACATAAATGTGGTAGAGCTCTTGGGATACTCCAGCGGAGATGACCACCTCTACTTGGTCTATGAGTATGTTCGGAATGGATCACTCAGTTCTCATCTGCACGATCCACTGCTGAAAGGTCATCGGCCTCTCTCTTGGACTGCAAGAACACAGATTGCACTGGATGCTGCGAAGGGTATTGAATACATTCATGACCACACAAAAGCTCGATATGTGCACCGAGACATAAAGACAAGTAACATTCTACTTGATGATGGGCTCAGAGCAAAGGTAGCAGATTTTGGACTGGCAAAACTTGTTGGGCGAACCAATGAGGAAGATTT---CCTGGCGACACGGCTGGTTGGAACGCCAGGCTACCTTCCTCCAGAATCCGTGAAGGAGCTCCAGGTGACCCCCAAAACTGATGTATTCGCATTTGGAGTGGTTCTAGCAGAACTGATCACGGGACATCGTGCACTTATCTGCGACAATCGAGA-------GCC---CAGCAGGATGAAATCACTAATTACAGTT----------------------------------------------------------------------------------------------------------------------------------------------------------------------------------------------------------------------------------------------------------------------------------------

>Dryas_EPR3_CDS

---------------------------------------------------------------------------------------------------ATGGCAT---CCCCTGATCTCCTTCCTTGTCTGCTCCTCTCTCTCTTGGTGAC-------TCTATTT--------TCTGCAGTTTTCGCCATTC---ATATATCTACCAAAA---TTTCTGTTCTATACCCTATGAATTGCTCTGCTAAA---AT-CATGACATGT--AATGCCTCAATGTACCACATCAA---CAACAGTCTGGA------GAAAGAAGAAATTGCCA---------------CTTATTACTCTGTCAGTTCA---TCCCAAATCAAAGATATAAAGCGA---------AAAAGAAAAAA---CGATTACCTCATAACTGTACCTTGTTCTTGCAAAAACATAGCTGGG------ACTATTGGATATTTCTATGATACATTCTACAGA---GTGAACGCATCAGACACATTCTTTGATGTTTCTACTCGAATTTATAGCGGGCAACCTTTGTATGTCAAG---GAAGAACTACGGCGATTTGTTCCAACAGCTGATTTTCCCATTCATCTTCCTTGTGGGTGTGTAGA---------------CAGTGACTCTCAGATTGTGG-TAACATATACAGTTC-AGAAGCAAGA-TACACTTGACCAAATTGCCATTCTGCTGTCTTCCAAGATGGAAAACCTACAAAACATGAACCACAAAATGATTGAGAATCCTTCATTCATAGTTCATGGTTGGGTGTTATTTGTGCCCAT---GGAAAAGAATGAAATTAAAT------------------------CAAAGACAGGAATCAGACACAG---GTGG---ACAATATTGATTGGCATATTATTAGCTG---TGGCATTACTTACAATGAGCACATTGATCCTTGTCC------TGTTTAGGCGAAAAGCA--CCCCAACAAAA-------TGTGGAAGTTCCA---AAAGCTGGATCGAAAAGCATGGCTGCCAACAG---------------------------ATC---TTTTTCATTGCATAATCAG-TTAC-TTCATAAAGAAAACATGGA---------AGTTTTTGAATCGGACGGACCAGTAGTATTTAGTCTTGAGGAGATTGAAGAGGCTACCAATTACTTTGATGAAACTAGGAAAATTGGAGAGGGTGGATATGGCAGTGTGTACTTGGGAGTACTAGGGGAGAAGGAAGTTGCCATAAAGAAGATGAGATCTAATAAGTCCAAAGAATTCTTTGCAGAGCTAAAGGTTTTATGCAAGGTCCATCACATTAATGTGGTGGAGCTTTTGGGGTATGCCAGTGGAGATGACCACCTCTACCTGGTCTATGAGTATGTTAAGAATGGATCACTTAATGATCATCTTCATGATCCATTAATTAAAGGTCACCAGCCACTCTCTTGGACTGCAAGAACACAAATTGCACTGGATACTGCAAAAGGTATTGAATACATCCATGACCACACAATAGACCGATATGTGCACCGTGATATAAAGACAAGTAACATTCTACTTGACGAAGGGCTCAGAGCAAAGGTAGCAGATTTTGGTTTGGCAAAGCTTGTTGGAAGAACCAATGAAGAAGATAT---TATAGCAACACGACTGGTTGGAACACCAGGCTATCTTCCTCCAGAATCCGTGAAGGAGCTCCAGGTAACTCACAAAACCGATGTATTTGCATTTGGAGTGGTACTAGGAGAGCTGATAACAGGGCAACGTGCACTTTTCCGTGACAACCGAGA-------GCC---TGAGAAGATGAAATCTTTGATTACAGTAGTAAAAAAGGTATTCCAAGATG---ATGATCCAGAATCAGCTTTAG------AGGCTGTCACAGATGGAAATCTTCGGGGCAACTATCCTATGGAAGAAATATTCAAGATGGCTGAAATTGCCGAGTGGTGTTTGAGTGAAGAAGCAGTGGAAAGGCCAGAGACGAGGGAGATTGTTGTGACTCTCTCACAAATTGTGACTTCCTCCATTGAGTGGGAAGCTTCACTAGGAGGGAACAGCCAGGTTTTCAGTGGT---GTATTTAATGGAAGATGA----

>QANT01001053.1_EPR3paralogcontig_CDS

---------------------------------------------------------------------------------------------------ATGGCAT---CCCCTCATCTCCTTCCTTGTCTGCTCCTCTCTTTCTTGGTGAC-------TTTATTT--------CCGGCAGTTTTCGCCATTC---ATATATCTACCAAAA---TATCTGTTCTATACCCTATGAATTGCTCTGCTAAA---AT-CATGACATGT--AATGCCTCAGTGTACCACATCAA---CCACAGTCTGGA------GAAAGAAGAAATTGCCA---------------CTTATTACTCTGTCAGTTCA---TCACAAATCAAACCTATAAAGCGA---------AAAAGCAAAGA---TGATTACCTCGTAACTGTACCTTGTTCTTGCAAAAACATATCTGGG------ACTATTGGATATTTCCATGATACATCATACAAA---GTGAAAGCATCAGACACATTCTTTGATGTTTCTACGAGAATTTATAGCGGGCAACCTTTGTATGTCAAA---GAAGAACAACGGCAATTTGTTCCAGCAGCTGATTTTCCCATTCATCTTCCTTGTGGGTGTGTAGA---------------TAGTGACTCTCAGATTGTGG-TGACATATACAGTTC-AGGAGCAAGA-TACACTTGGAGAAATTGCCATTCTGCTGTCTGCCAAGATGGAAAACATAGAAAACATGAACCCAAATATGACTCAGAACCCATCATTCATAGTTAATGGTTGGGTGTTGTTTGTGCCCAT---GGAAAAGAATGGAATTAAAT------------------------CAAAGACAGGAATCAGACACAG---GTGG---ACAATATTGATTGGCATATTATTAGCTG---TGACGTTACTTACAATCAGCACATTGATACTTGTCC------TGTTTAGGAGAAAAGCA--ACCCAACAAAA-------TGTGGAAGTTCCA---AAAGCTGGATCGAAAAGAATGGCTGCCAACAG---------------------------ATC---TTTTTCATTGCAAAATCAA-TTAC-TTCATAAAGAAAACATGGA---------AGTTTTTGAATCAGACGGACCAGTAGTGTTTAGTCTTGAGGAGATTGAAGAGGCTACCAATTACTTTGATGAAACTAAGAAAATTGGAGAGGGTGGATATGGAAGTGTGTACTTCGGAGTACTAGGGAAGAAGGAAGTTGCCATAAAGAAGATGAGATCTAATAAGTCCAAAGAATTCTTTGCAGAGCTAAAGGTTTTATGCAGAGTCCATCACATTAATGTGGTGGAGCTTTTGGGGTATGCCAGTGGAGATGACCACCTCTACCTGGTCTATGAGTATGTTCAGAATGGATCACTTAATGATCATCTTCATGATCCATTACTTAAAGGTCACCAGCCACTCTCTTGGACTGCAAGAACACAAATTGCACTGGATACTGCAAAAGGTATTGAATACATCCATGACCACACAAAAGACCGATATGTGCACCGTGATATAAAGACAAGTAACATTCTACTTGACGAAGGGCTCAGAGCAAAGGTAGCAGATTTTGGTTTGGCAAAGCTTGTTGGAAGAACCAATGAAGAAGAAAT---TATAGCAACACGACTGGTTGGAACACCAGGCTATCTTCCCCCAGAATCCGTGAAGGAGCTCCAGGTGACTCACAAAACCGATGTATTTGCATTTGGAGTGGTACTAGGAGAGCTGATAACAGGACAACGTGCACTTTTCCGTGACAACCGAGA-------GCC---TGAGAAGATGAAATCTTTGATTACAGTCGTAAAAAAGGTATTCCAAGATG---ATGATCCAGAATCCGCTTTAG------AGGCTGTCACAGATGGAAATCTTTTGGGCAACTATCCTATGGAAGAAATATTCAAGATGGCTGAAATTTCTGAGTGGTGTTTGAGTGAAGAAGCAGTGGAAAGGCCAGAGATGCGGGAGATTGTAGTGACACTCTCACAAATTGTGATATCCTCCATTGAGTGGGAAGCTTCACTAGGAGGGAACAGCCAGGTTTTCAGTGGT---GTATTTAATGGAAGATGA----

>TorEPR_CDS

---------------------------------------------------------------------------------------------------ATGGCAA---CCCATCATCTCCTTCCCTATCTTCTCCTTATGTTCC------C-------TCTTTGT--------TCTCGAGTTTTTACACTCC---ATGTGTCTATGAAAG---AATCCCTTATGTACCCTTTTAGCTGCTCAGCACAA---AT-CAAGACGTGT--AATGCTTCATTGTACCATATTAA---TGAAGGTCTCAC------AATAGAAGAAATTGCTG---------------CTTATTACAAGGTCAACGCATCCTCCCAAATTGAGCCCATAATGCAT---------GACAGCAGGAA---AGATTACCTCATAACAGTACCTTGTTCCTGCTCATCCATGTTTGGC------GTAACCGGATATTTTTACAACACAACCTACAAC---GTCAAATTAAATGACACTTTTCTAGATGTTTCAGCTAAGTACTATAGTGGACAAGCTTGGAGATTTGAA---GAAGAAGACCAATATTTCAAGCCTGATAAAAATTTTACCATGCATCTTCTTTGTGGTTGTCTAGA---------------AAGTGACTCCGAAATTGTGG-TAACCTACACAGTTC-AGGACCATGA-TACACTACTAAATATTGCAGCTCGGTTGTCAGCCAATTTCGACAACTTGGTGAGATTGAATGGACATTTGACTCAAAACCCTGCTTTCATTTTGGTAGGCTGGGTCTTGTTTGTGCCCAA---GGAGAAAAATGGAATTGAAA------------------------CATCAACGGGTATGAGAGAGAA---GTGG---AAAATTGTAACTGGTATATTGTTAATTG---TGACGCTGCTTTCAACTGGTGCATTGATGGTCATTC------TTCTCAGAAGAAAAAGA--TTGCAGCAAAAG----AAAGTGGAAGATCCT---AAAGTCATATCCAAAATTGTGACTTTTAAAAA---------------------------ATC---TCTCTCCTTGCAGAATCCT-TTCC-TCCCTAAAGAAAATTTTGA---------AGATTTTGATTCAGAAAAAACTGTAGTATTTAGTCTTGAGGCGATCGAAGAGGCCACTGGACACTTTGACGAAAGTAAGAAAGTAGGAGAGGGGGGATATGGGTGTGTGTATTTTGGCATACTAGGAGAGAAGGAGGTGGCCATAAAGAAGATGATATCGAATAAATCGAAGGAGTTCTTTGCAGAGCTAAAGGTGTTATGCAAGATCCATCATATAAACGTGGTGGAGCTTTTGGGGTATGCTAGTGGAGATGACCACCTCTACTTGGTTTACGAGTATGTTCCGAACGGATCACTGAGCGATCATCTTCATGATCCGTTACTGAAAGGTCACCAGCCTCTGTCCTGGACTGCAAGAGCACAGATTGCACTTGGCGCTGCAAACGGTATTGAGTACATTCATGACCATACAAAAGCACGGTATGTGCACCGTGATATAAAAACAACTAACATCCTACTTGATGAGGGCCTCAGAGCTAAGGTAGCAGATTTTGGGTTAGCAAAGCTTGTTGAACGAGCCAATGAAGATGATTT---CATAGCGACACGACTAGTTGGCACACCAGGCTACCTTCCTCCAGAATCGGTGAAGGAGCTACAGGTAACCCCGAAGACAGATGTATTTGCATTTGGAGTGGTACTAGCAGAGCTGATTACAGGGCAACGCGCACTTATCCGTGACAACCGAGA-------GCC---TAACAAGATGAAATCTCTAAAAACAGTTGTTAAGAGAATATTCCAAGATG---AAGATCCAGAATCAGCTTTAG------ATGCCGAAATAGACGGAAATCTCAGGGGCAGCTACCCTAGTGAGGATGTCTTCAAGATGGCAGAAGTTGCGGAGTTGTGCTTACGTGAAGAAGCAGTGGACAGACCAGAGATGAGGGATATTGTGGTGACACTGTCTCAAATAGTGATGTCCTCGATAGAGTGGGAAGCATCACTTGGAGGGAACAGCCAGGTCTTCAGTGGG---GTATTCACTGGCAGATGA----

>Trema_tomentosa_EPRa_CDS

---------------------------------------------------------------------------------------ATGCCAAAAACCATGGCAA---CCCATCATCTCCTTCCCTATCTTCTCCTTATGTTCC------C-------TCTTTGT--------TCTCGAGTTTTTACACACC---ATGTGTCTATGAAAG---AATCCCTTATGTACCCTTTTAACTGCTCAGCACAA---AT-CAAGACGTGT--AATGCTTCATTGTACCATATCAA---TGAAGGTCTCAC------AATAGAAGAAATTGCTG---------------CTTATTACAAGGTCAACGCATCCTCCCAAATTGAGCCCATAATGCAT---------GACAACAGGGA---AGATTACCTCATAACAGTACCTTGTTCCTGCTCATCCATGTTTGGC------GTAACCGGATATTTTTACAACACGACCTACAAC---GTCAAATTAAATGACACTTTTCTAGATGTTTCAGCTAAATACTATAGTGGACAAGCTTGGAGATTTGAA---GAAGAAGACCAATATTTCAAGCCTGATAAAAATTTTACCATGCATCTTCTTTGTGGTTGTCTAGA---------------AAGTGACTCCGAAATTGTGG-TAACCTACACAGTTC-AGGACCATGA-TACACTATTAAATATTGCAACTCTGTTGTCAGCCAATTTCGACAACTTGGTGAGATTGAATGGACATTTGACTCAAAACCCTGCTTTCATTGTGGTAGGCTGGGTCTTGTTTGTGCCCAA---GGAGAAAAATGGAATTAAAA------------------------CATCAACGGGTATGAGAGAGAA---GTGG---AAAATTGTAACTGGTATATTGTTAATTG---TSACGCTGCTTTCAACTGGTGCATTGATGGTCATTC------TTCTCAGAAGAAAACGA--TTGCAGCAAAAG----AAAGTGGAAGATCCT---AAAGTCCTATCCAAAATTGTGACTATTAAAAA---------------------------ATC---TCTCTCCTTGCAGAATCCT-TTCC-TTCCTAAAGAAAATTTTGA---------AGATTTGGATTCTGAAAAAACTGTAGTATTTAATCTTGAGGCGATCGAAGAGGCCACTGGACACTTTGACGAAAGTAAGAAAGTAGGAGAGGGGGGATATGGGTGTGTGTATTTTGGCATACTAGGAGAGAAGGAGGTTGCCATAAAAAAGATGAGATCGAATAAATCGAAGGAGTTCTTTGCAGAGCTAAAGGTCTTATGCAAGATCCATCATATAAACGTGGTGGAGCTTTTGGGGTATGCTAGTGGAGATGACCACCTCTACTTGGTTTACGAGTATGTTCCGAACGGATCACTGAGCGATCATCTTCAAGATCCGTTACTGAAAGGTCACCAGCCTCTGTCCTGGACTGCAAGAGCACAGATTGCACTTGGCGCTGCAAAGGGTATTGAGTACATTCATGACCATACAAAAGCACGGTATGTGCACCGTGACATAAAAACAACTAACATCCTACTTGATGAGGGCCTCAGAGCTAAGGTAGCAGATTTTGGGTTAGCAAAGCTTGTTGAACGAGCCAATGAAGATGATTT---CATAGCGACACGACTAGTTGGCACACCAGGCTACCTTCCTCCAGAATCGGTGAAGGAGCTCCAGGTAACCCCGAAAACAGATGTATTTGCATTTGGAGTGGTACTAGCAGAGCTGATTACAGGACAACGCGCACTTATCCGTGACAACCGAGA-------GCC---TAACAAGATGAAATCTCTAGTAACAGTTGTTAAGAGAATATTCCAAGATG---AAGATCCAGAATCAGCTTTAG------AAGCCGAAATAGACGGAAATCTCCGGGGCAGCTACCCTAGTGAGGATGTCTTCAAGATGGCAGAAGTTGCGGAGTTGTGCTTACGTGAAGAAGCAGTGGACAGACCAGAGATGAGGGATATTGTGGCGACACTGTCTCAAATAGTGATGTCCTCAATAGAGTGGGAAGCATCACTTGGAGGGAACAGCCAGGTCTTCAGTGGG---GTATTCACTGGCAGATGA----

>PanWU01x14_asm01_scf00342

---------------------------------------------------------------------------------------------------ATGGCAA---CCCATCATCTCCTTCCCTATCTTCTCCTTATGTTCC------C-------TCTTTGT--------TCTCGAGTTTTTACACTCC---ATGTGTCTATGAAAG---AATCTCTTATGTACCCTTTTAACTGCTCGGCACAA---AT-CAAGGCGTGT--AATGCTTCATTGTACCATATCAA---TGAAGGTCTCAC------AATAAAAGAAATTGCTG---------------CTTATTACAAGGTCAACGCATCCTCCCAAATTGAGTCCATAATGCAT---------AACAACAGGGA---AGATTACCTCATAACAGTACCTTGTTCCTGCTCATCCATGTTTGGC------GTAACCGGATATTTTTACAACACGACCTACAAC---GTCAAATTAAATGACACTTTTCTAGATGTTTCAGCTAAGTACTATAGTGGACAAGCTTGGAGATTTGAA---GAAGAAGTCCAATATTTCAAGCCTGATAAAAATTTTACCATGCATCTTCTTTGTGGTTGTCTAGA---------------AAGTGACTCCGGAATTGTGG-TAACCTACACAGTTC-AGGACCATGA-TACACTATAAAATATTGCAAATCTGTTGTCAGCCAAATTCGACAACTTGGTGAGATTGAATGGATATTTGACTCAAAACCCTGCTTTCATTGAGGTAGGCTGGGTCTTGTTTGTGCCCAA---GGAGAAAAATGGAATTAAAA------------------------CATCAACGGGTACGAGAGAGAA---GTGG---AAAATTGTAACTGGTATATTGTTAATTG---TGACGCTGCTTTCAACTGGTGCATTGATGGTCATTC------TTCTCAGAAGAAAAAGA--TTGCAGCAAAAG----AAAGTGGAAGATCCT---CAAGTCCTATCAAAAATTGTGACTACTAAAAA---------------------------ATC---TCTCTCCTTGCAGAATCCT-TTCC-TTCCTAAAGAAAATTTTGA---------AGATTTTCATTCAGAAAAAACTGTAGTATTTAGTCTTGAGGCGATCGAAGAGGCCACTGGACACTTTGACGAAAGTAAGAAAGTAGGAGAGGGGGGATATGGGTGTGTGTATTTTGGCATACTAGGAGAGAAGGAGGTTGCCATAAAGAAGATGAGATCGAATAAATCGAAGGAGTTCTTTGCAGAGCTAAAGGTCTTATGCAAGATCCATCATATAAACGTGGTGGAGCTTTTGGGGTATGCTAGTGGAGATGACCACCTCTACTTGGTTTACGAGTATGTTCCGAACGGATCACTGAGCGATCATCTTCATGATCCGTTATTGAAAGGTCACCAGCCTCTGTCCTGGACTGCAAGAGCACAGATTGCACTTGGCGCTGCAAAGGGTATTGAGTACATTCATGACCTTACAAAAGCACGGTATGTGCACCGTAATATAAAAACAACTAACATCCTACTTGATGAGGGCCTCAGAGCTAAGGTAACAGATTTTGGGTTAGCAGAGCTTGTTGAACGAGCCAATGAAGAGGATTT---CATAGCGACACGACTAGTTGGCACACCAGGCTACCTTCCTCCAGAATCGGTGAAGGAGCTTCAGGTCACCCCGAAAACAGATGTATTTGCATTTGGAGTGGAACTAGCTGAGCTGATTACAGGGCAACGCGCACTTATCCGTGACAACCGAGA-------GCC---TAACAAGATGAAATCTCTAATAACAGTTGTTAAAAGAATATTCCAAGATG---AAGATCCAGAATCAGCTTTAG------AAGCCGAAATAGACGGAAATCTCCGGGGCAGCTACCCTAGTGAGGATGTCTTCAAGATGACAGAAGTTGCGGAGTTGTGCTTACGTGAAGAAGCAGTGGACAGACCAGAGATGAGGGATATTGTGGTGACACTGTCTCAAATAGTGATGTCCTCAATAGAGTGGGAAGCATCACTTGGAGGGAACGGCCAGGTCTTCAGTGGG---GTATTCACTGGCAGATGA----

>Ochtri_scaffold1293_CDS_v2

---------------------------------------------------------------------------------------------------ATGGAAA---CTCATGATCTCCTTCCCTGTCTTCTCCCCCTCCTTTTGACAAC-------CCTGTTT--------TCTGTGGTTTCCACAAATATAGATGTGTCTATCAAAA---GCACTCTTATGAGCCCTTTTAGCTGTTCTTTACAA---AT-CAACACGTGT--AATGCTTCTCTATACCACATCAACTATGATACTCTCCG------GGAAGATGAAATTGCTG---------------CCTTTTACTCTGTGAACGT---TTCCCAAATGAAGCCTATAACGAAC---------GGCAATAAGCA---ACATTACCTTATAAAAGTACCTTGTTCTTGCAATACTATATTGGGG------AATACTGGATATTTCTATGATACAATCTACAAA---GTGAAACCAGGTGACAGTTTTGAGGATGTTTCAGCTCAGATTTATAGTGGTCAAGCTT------TTAGA---GATGGGACACAAAACTTTATGCCTGAAACAGATTTTAGTATGCATCTTCTATGTGGGTGTGTAGA---------------AAGTGACAGACAGACCGTGG-CAACATACACTGTTC-AGTTAGGTGA-CACAATTTCAAGTATTGCAGCTCTTTTGTCTGCCAAAGTGGAGAACGTAGTGAGCATGAATAGAAATCTGACTGAGAATCCAGCTTTTATAGATATAGGTTGGGTGTTGTTTGTGCCTAT---GGAGAAGAATGGAATTGAAAA---------------------ACCACCAGCAGGATGGAAACATAA---AAAG---ATGATAATAATCATCATCGTATTAGCTGCTGTAGCAATGCTTTCATTGGCTGCATTGTTAGCTGTCC------TTCTCAAGAGAGAAAGA--TCCAAGCAGAAA----AGCGAAGAAGATCCA---AAAGCTATATCCAAAACTCTGAGTGCTAAA------------------------------TC---CTTTTCCTTGCAGAAACAT-TTCA-TAAATAACGAAAACATGCA---------AGATTTTGAACCAGAAAGACCAGTAATATTTACTCTAGAGGAGATTGAAGAAGCTACAAGTCACTTTGATGAATCTAAGAAAATTGGAGAAGGTGGATATGGGAGTGTGTACTTTGGAGTATTAGGAGAGCAGGAGGTTGCCATAAAGAAGATGAAATCGAATTTGTCCAAAGAGTTCTTCGCGGAGCTAAAGGTTTTATGCAAGATCCATCACATCAATGTTGTGGAGCTTTTGGGGTATGCCTGTGGAGATGACTACCTTTACTTGGTTTATGAATTCATTCAGAATGGATCTCTGAGTGATCATCTTCATGATCCATTACTAAAAGGTCACCAGCCTCTATCCTGGGCTGCAAGAACACAGATTGCCCTTGATGCTGCAATGGGTATTGAATACATCCACGATCATACAAAAGCACGGTACGTGCACCGTGATATAAAGACAAGTAACATTCTACTTGATGAAGGGCTCAGAGCTAAGGTAGCAGATTTTGGGTTAGCAAAGCTTGTTGGAAGAGCCAATGAGGAAGATAC---AATGGCAACAAGATTGGTTGGAACACCAGGCTATCTCCCTCCGGAATCTTTGAAGGAGCTCCAAGTGACCCCAAAAGCAGATGTGTTTGCATTTGGAGTTGTTTTGGCAGAGCTGATTACAGGACAACGTGCACTTGTACGTGACAACCGAGT-------TCC---AAACAAGATGAAATCTCTAATAACAGTTGTTAAAAGTATATTCGGAGACG---AAAATCCAGAGTCCGCTTTAG------AGGAAGAAATAGATGGAAATCTCCGGAGCAGCCACCCTATGGAAGATGTTTTCAGGATGGCAGAGATTTCGCAGTTGTGTTTGAGTGAAGAAGCAGTTGAGAGACCAGAGATGAGGGACATTGTTGTTGCTTTATCTCATATTGTATCGTCTGCAAGAGAATGGGAAGCTTCACTAGGAGGTAACAGCCAGGTTTTCAGTGGT---CTGTTTAATGGAAGATAA----

>arahy.Tifrunner.gnm2.ann1.REE5L6.1

---------------------------------------------------------------------------------------------ATGGCTTCTTCTC---TCAATAATCTTCTTTGTCTTCTCTTCTCTCTTATGGCAACTTC-------ATCTTCT--------ATCAGAGTCTCTGCATTTC---GTGTTTCAATGAAAA---CAACATACATGGACCCGCTAAGTTGCTCTGTAGAGGCTGC-CAATACATGC--ACTGCCTCACTCTACCACATAACTCATGATGATTACAG------CCTTGAAGAAATTGCAT---------------TTTATTACTCTGTTAACTCT---TCACAAATCAAGCCTATTAAGTAT---------GGAACAAGGCA---AGATTACCTCATAACAGTTCCTTGTTCTTGCAAGAACACACAAAAC------CTCAGCGGGTATTTCTATCATACAACCTACAAAAATGTGAAGCATGGTGACATCTTTGTGGACATATCAGCTTCTGTTTACAGTGGACAAGCCATGCTGATCACCC------AAAGTTTAAT------TCCAGGCGAAGATCTAGAAATAGACATTCCATGTGGGTGTTCCGA---------------GGATGAGGGTCAAAGGGTTG-TCACATATACAGTGC-AGCAGAATGA-TACACCAGAAACAATTTCCCTTTTGCTTAATGCTACCCTGCCTGCCTTCCTGAGAATGAACAAGATTCTAGCTCAGAAACCTGGTTTCATAGATATTGGTTGGGTGCTCTTTGTTCCTTT---GGAATTGAATGGGGTTCATGG-------GGTTCCTCCACAACCCACA--GAAAAAACGAAACGGAA---CTGG---CCAATGATAGTTGGTATCGTCGTGGGAG---TGCTGTTACTTTCAGTTATCATTATCACCATTCTCATA---ATTCTTTGGAGAAGGAGA---GTCCATCAAAT---CACCAGAAATGATTC---CACAGTTGTCTCTAAAAGATCATTTGTCAACAG---------------------------AAA---TATTACCTTGCATAACTCC-ATCC-TTTTCAAAGAATATATGGGAGATGTAATGCAAATTGAATCAGAAAGGCCACTGATATATGGTCTTGAGGAGATAGAAGAGGCTACAAATAACTTTGATGAAGCTCGAAAAATCGGAGTTGGCGGATATGGGAGTGTTTATTTTGGAATCTTAGAGCAGAAGGAGGTTGCTGTGAAAAAGATGAGGTCTAATAAGACTAAGGAATTCTATACGGAACTCAAGGCCTTGTGTAAAATCCATCATATCAACATTGTGGAGCTATTAGGATATGCCAGAGGAGAAGATCACCTCTATTTGGTGTATGAGTATGTTTCAAATGGATCTCTCAGTGAACATATTCATGATCCATTAGAGCAAGGGCACCAACCTCTTTCTTGGAATGCAAGGGTTCATATTGCACTCGATGCAGCAAGAGGTATTGAATACATACATGATCATACAAAAGCTCGATATGTGCATCGCGACATAAAGACTAGCAATATTCTTGTTGATCAGAAGCTCAGAGCTAAGGTAGCAGATTTTGGACTTGCAATGCTGGTGGACAGAAGCAATGATGAAAATTT---CATAGCAACAAGGCTTGTTGGAACACCAGGATACCTTCCACCCGAATCTGTGAAGGAGCTTCAGGTGACCCCAAAAACCGATGTGTTTGCATTTGGAGTGGTTCTAGCAGAACTGATAACAGGGAAACGTGCACTGTTTCGGGACAACAACGA-------AGAAGGCACCAAAATGAAATCACTTATTTCACTTGTACATCAAATATTCCAAGATG---CAGAGGCAGAGAATGGTCTAG------AAGATGTGATAGATAAGAACCTTGAAGGAAACTATCCCATTGAATACGTGTTGAAGGTAGCAGAGATAGCAGGTCGGTGCTTGCAAGAAGATCCAGTAGAAAGGCCTGAAATGAGGGACTTGGTTGGAGCACTGTCACAGATAGTGATGAACTCCATAGAATGGGAAGCATCACTTGGTGGAAACAGCCAAGTCTTCAGTGGT---GTCTTTACTGGAAGATGA----

>SBbav42xvGysNbOiTKsv+ES1sMc.1632394383523.585238

------------------------------------------------------------------------------------------------------------------------------------------------------------------------------------------------------------ATGAAAA---CAACATACATGGACCCGCTAAGTTGCTCTGTAGAGGCTGC-CAATACATGC--ACTGCCTCACTCTACCACATAACTCATGATGATTACAG------CCTTGAAGAAATTGCAT---------------TTTATTACTCTGTTAACTCT---TCACAAATCAAGCCTATTAAGTAT---------GGAACAAGGCA---AGATTACCTCATAACAGTTCCTTGTTCTTGCAAGAACACACAAAAC------CTCAGCGGGTATTTCTATCATACAACCTACAAAAATGTGAAGCATGGKGACATCTTTGTGGACATATCAGCTTCTGTTTACAGTGGACAAGCCATGCTGATCACCC------AAAGTTTAAT------TCCAGGCRAAGATCTAGAAATAGACATTCCATGTGGGTGTTCCGA---------------GGATGAGGGTCAAAGGGTTG-TCACATATACAGTGC-AGCAGAATGA-TACACCAGAAACAATTTCCCTTTTGCTTAATGCTACCCTGCCTGCCTTSCTGAGAATGAACAAGATTCTAGCTCAGAAACCTGGTTTCATAGATATTGGTTGGGTGCTCTTTGTTCCTTT---GGAATTGAATGGGGTTCATGG-------GGTTCCTCCACAACCCACA--GAAAAAACGAAACGGAA---CTGG---CCAATGATAGTTGGTATCGTCGTGGGAG---TGCTGTTACTTTCAGTTATCATTATCACCATTCTCATA---ATTCTTTGGAGAAGGAGA---GTCCATCAAAT---CACCAGAAATGATTC---CACAGTTGTCTCTAAAAGATCATTTGTCAACAG---------------------------AAA---TATTACCTTGCATAACTCC-ATCC-TTTTCAAAGAATATATGGGAGATGTAATGCAAATTGAATCAGAAAGGCCACTGATATATGGTCTTGAGGAGATAGAAGAGGCTACAAATAACTTTGATGAAGCTCGAAAAATCGGAGTTGGCGGATATGGGAGTGTTTATTTTGGAATCTTAGAGCAGAAGGAGGTTGCTGTGAAAAAGATGAGGTCTAATAAGACTAAGGAATTCTATACGGAACTCAAGGCCTTGTGTAAAATCCATCATATCAACATTGTGGAGCTATTAGGATATGCCAGAGGAGAAGATCACCTCTATTTGGTGTATGAGTATGTTTCAAATGGATCTCTCAGTGAACATATTCATGATCCATTAGAGCAAGGGCACCAACCTCTTTCTTGGAATGCAAGGGTTCATATTGCACTCGATGCAGCAAGAGGTATTGAATACATACATGATCATACAAAAGCTCGATATGTGCATCGCGACATAAAGACTAGCAATATTCTTGTTGATCAGAAGCTCAGAGCTAAGGTAGCAGATTTTGGACTTGCAATGCTGGTGGACAGAAGCAATGATGAAAATTT---CATAGCAACAAGGCTTGTTGGAACACCAGGATACCTTCCACCCGAATCTGTGAAGGAGCTTCAGGTGACCCCAAAAACCGATGTGTTTGCATTTGGAGTGGTTCTAGCAGAACTGATAACAGGGAAACGTGCACTGTTTCGGGACAACAACGA-------AGAAGGCACCAAAATGAAATCACTTATTTCACTTGTACATCAAATATTCCAAGATG---CAGAGGCAGAGAATGGTCTAG------AAGATGTGATAGATAAGAACCTTGAAGGAAACTATCCCATTGAATACGTGTTGAAGGTAGCAGAGATAGCAGGTCGGTGCTTGCAAGAAGATCCAGTAGAAAGGCCTGAAATGAGGGACTTGGTTGGAGCACTGTCACAGATAGTGATGAACTCCATAGAATGGGAAGCATCACTTGGTGGAAACAGCCAAGTCTTCAGTGGT---GTCTTTACTGGAAGATGA----

>aradu.Aradu.J0SGA:cds:1

------------------------------------------------------------------------------------------------------------------------------------------------------------------------------------------------------------ATGAAAA---CAACATACATGGACCCGCTAAGTTGCTCTGTAGAGGCTGC-CAATACATGC--ACTGCCTCACTCTACCACATAACTCATGATGATTACAG------CCTTGAAGAAATTGCAT---------------TTTATTACTCTGTTAACTCT---TCACAAATCAAGCCTATTAAGTAT---------GGAACAAGGCA---AGATTACCTCATAACAGTTCCTTGTTCTTGCAAGAACACACAAAAC------CTCAGCGGGTATTTCTATCATACAACCTACAAAAATGTGAAGCATGGTGACATCTTTGTGGACATATCAGCTTCTGTTTACAGTGGACAAGCCATGCTGATCACCC------AAAGTTTAAT------TCCAGGCGAAGATCTAGAAATAGACATTCCATGTGGGTGTTCCGA---------------GGATGAGGGTCAAAGGGTTG-TCACATATACAGTGC-AGCAGAATGA-TACACCAGAAACAATTTCCCTTTTGCTTAATGCTACCCTGCCTGCCTTCCTGAGAATGAACAAGATTCTAGCTCAGAAACCTGGTTTCATAGATATTGGTTGGGTGCTCTTTGTTCCTTT---GGAATTGAATGGGGTTCATGG-------GGTTCCTCCACAACCCACA--GAAAAAACGAAACGGAA---CTGG---CCAATGATAGTTGGTATCGTCGTGGGAG---TGCTGTTACTTTCAGTTATCATTATCACCATTCTCATA---ATTCTTTGGAGAAGGAGA---GTCCATCAAAT---CACCAGAAATGATTC---CACAGTTGTCTCTAAAAGATCATTTGTCAACAG---------------------------AAA---TATTACCTTGCATAACTCC-ATCC-TTTACAAAGAATATATGGGAGATGTAATGCAAATTGAATCAGAAAGGCCAGTGATATATGGTCTTGAGGAGATAGAAGAGGCTACAAATAACTTTGATGAAGCTCGAAAAATCGGAGTTGGCGGATATGGGAGTGTTTATTTTGGAATCTTAGAGCAGAAGGAGGTTGCTGTGAAAAAGATGAGGTCTAATAAGACTAAGGAATTCTATGCGGAACTCAAGGCCTTGTGTAAGATCCATCATATCAACATTGTGGAGCTATTAGGATATGCCAGAGGAGAAGATCACCTCTATTTGGTGTATGAGTATGTTTCAAATGGATCTCTCAGTGAACATATTCATGATCCATTAGAGCAAGGGCACCAACCTCTTTCTTGGAATGCAAGGGTTCAGATTGCACTCGATGCAGCAAGAGGTATTGAATACATACATGATCATACAAAAGCTCGATATGTGCATCGCGACATAAAGACTAGCAATATTCTTGTAGATGAGAAGCTCAGAGCTAAGGTAGCAGATTTTGGACTTGCAATGCTGGTGGACAGAAGCAATGATGAAAATTT---CATAGCAACAAGGCTTGTTGGAACACCAGGATACCTTCCACCCGAATCTGTGAAGGAGCTTCAGGTGACCCCAAAAACCGATGTGTTTGCATTTGGAGTGGTTCTAGCAGAACTGATAACAGGGAAACGTGCACTGTTTCGGGACAACAACGA-------AGAAGGCACCAAAATGAAATCACTTATTTCACTTGTACATCAAATATTCCAAGATG---CAGAGGCAGAGAATGGTCTAG------AAGATGTGATAGATAAGAACCTTGAAGGAAACTATCCCATTGAATACGTGTTGAAGGTAGCAGAGATAGCAGGTCGGTGCTTGCAAGAAGATCCAGTAGAAAGGCCTGAAATGAGGGACTTGGTTGGAGCACTGTCACAGATAGTGATGAACTCCATAGAATGGGAAGCATCACTTGGTGGAAACAGCCAAGTCTTCAGTGGT---GTCTTTACTGGAAGATGA----

>Araip.19T2H

------------------------------------------------------------------------------------------------------------------------------------------------------------------------------------------------------------ATGAAAA---CAACATACATGGACCCGCTAAGTTGCTCTGTAGAGGCTGC-CAATACATGC--ACTGCCTCACTCTACCACATAACTCATGATGATTACAG------CCTTGAAGAAATTGCAT---------------TTTATTACTCTGTTAACTCT---TCACAAATCAAGCCTATTAAGTAT---------GGAACAAGGCA---AGATTACCTCATAACAGTTCCTTGTTCTTGCAAGAACACACAAAAC------CTCAGCGGGTATTTCTATCATACAACCTACAAAAATGTGAAGCATGGGGACATCTTTGTGGACATATCAGCTTCTGTTTACAGTGGACAAGCCATGCTGATCACCC------AAAGTTTAAT------TCCAGGCAAAGATCTAGAAATAGACATTCCATGTGGGTGTTCCGA---------------GGATGAGGGTCAAAGGGTTG-TCACATATACAGTGC-AGCAGAATGA-TACACCAGAAACAATTTCCCTTTTGCTTAATGCTACCCTGCCTGCCTTGCTGAGAATGAACAAGATTCTAGCTCAGAAACCTGGTTTCATAGATATTGGTTGGGTGCTCTTTGTTCCTTT---GGAATTGAATGGGGTTCATGG-------GGTTCCTCCACAACCCCCA--GAAAAAACGAAACGGAA---CTGG---CCAATGATAGTTGGTATCGTCGTGGGAG---TGCTGTTACTTTCAGTTATCATTATCACCATTCTCATA---ATTCTTTGGAGAAGGAGA---GTCCATCAAAT---CACCAGAAATGATTC---CACAGTTGTCTCTAAAAGATCATTTGTCAACAG---------------------------AAA---TATTACCTTGCATAACTCC-ACCC-TTTACAAAGAATATATGGGAGATGTAATGCAAATTGAATCAGAGAGGCCAGTGATATATGGTCTTGAGGAGATAGAAGAGGCTACAAATAACTTTGATGAAGCTCGAAAAATCGGAGTTGGCGGGTATGGGAGTGTTTATTTTGGAATCTTAGAGCAGAAGGAGGTTGCTGTGAAAAAGATGAGGTCTAATAAGACTAAGGAATTCTATGCGGAACTCAAGGCCTTGTGTAAGATCCATCATATCAACATTGTGGAGCTATTAGGATATGCCAGAGGAGAAGATCACCTCTATTTGGTGTATGAGTATGTTTCAAATGGATCTCTCAGTGAACATATTCATGATCCATTAGAGCAAGGGCACCAACCTCTTTCTTGGAATGCAAGGGTTCAGATTGCACTCGATGCAGCAAGAGGTATTGAATACATACATGATCATACAAAAGCTCGATATGTGCATCGCGACATAAAGACTAGCAATATTCTCATTGATGAGAAGCTCAGAGCTAAGGTAGCAGATTTTGGACTTGCAATGCTGGTGGACAGAAGCAATGATGAAAATTT---CATAGCAACAAGGCTTGTTGGAACACCAGGATACCTTCCACCCGAATCTGTGAAGGAGCTTCAGGTGACCCCCAAAACCGATGTGTTTGCATTTGGAGTGGTTCTAGCAGAACTGATAACAGGGAAACGTGCACTGTTTCGGGACAAGAACGA-------AGAAGGCACCAAAATGAAATCACTTATTTCACTTGTACATCAAATATTCCAAGATG---CAGAGCCAGAGAATGGTCTAG------AAGATGTGATAGATAAGAACCTTGAAGCAAACTATCCCATTGAATACGTGTTGAAGGTAGCAGAGATAGCAGGTCGGTGCTTGCAAGAAGATCCAGTAGAAAGGCCTGAAATGAGGGACTTGGTTGGAGCACTGTCACAGATAGTGATGAACTCCATAGAATGGGAAGCATCCCTTGGTGGAAACAGCCAAGTCTTCAGTGGT---GTCTTTACTGGAAGATGAACAA

>SBbav42xvGysNbOiTKsv+ES1sMc.1632394372416.444640

------------------------------------------------------------------------------------------------------------------------------------------------------------------------------------------------------------ATGAAAA---CAACATACATGGACCCGCTAAGTTGCTCTGTAGAGGCTGC-CAATACATGC--ACTGCCTCACTCTACCACATAACTCATGATGATTACAG------CCTTGAAGAAATTGCAT---------------TTTATTACTCTGTTAACTCT---TCACAAATCAAGCCTATTAAGTAT---------GGAACAAGGCA---AGATTACCTCATAACAGTTCCTTGTTCTTGCAAGAACACACAAAAC------CTCAGCGGGTATTTCTATCATACAACCTACAAAAATGTGAAGCATGGGGACATCTTTGTGGACATATCAGCTTCTGTTTACAGTGGACAAGCCATGCTGATCACCC------AAAGTTTAAT------TCCAGGCAAAGATCTAGAAATAGACATTCCATGTGGGTGTTCCGA---------------GGATGAGGGTCAAAGGGTTG-TCACATATACAGTGC-AGCAGAATGA-TACACCAGAAACAATTTCCCTTTTGCTTAATGCTACCCTGCCTGCCTTGCTGAGAATGAACAAGATTCTAGCTCAGAAACCTGGTTTCATAGATATTGGTTGGGTGCTCTTTGTTCCTTT---GGAATTGAATGGGGTTCATGG-------GGTTCCTCCACAACCCACA--GAAAAAACGAAACGGAA---CTGG---CCAATGATAGTTGGTATCGTCGTGGGAG---TGCTGTTACTTTCAGTTATCATTATCACCATTCTCATA---ATTCTTTGGAGAAGGAGA---GTCCATCAAAT---CACCAGAAATGATTC---CACAGTTGTCTCTAAAAGATCATTTGTCAACAG---------------------------AAA---TATTACCTTGCATAACTCC-ACCC-TTTACAAAGAATATATGGGAGATGTAATGCAAATTGAATCAGAGAGGCCAGTGATATATGGTCTTGAGGAGATAGAAGAGGCTACAAATAACTTTGATGAAGCTCGAAAAATCGGAGTTGGCGGGTATGGGAGTGTTTATTTTGGAATCTTAGAGCAGAAGGAGGTTGCTGTGAAAAAGATGAGGTCTAATAAGACTAAGGAATTCTATGCGGAACTCAAGGCCTTGTGTAAGATCCATCATATCAACATTGTGGAGCTATTAGGATATGCCAGAGGAGAAGATCACCTCTATTTGGTGTATGAGTATGTTTCAAATGGATCTCTCAGTGAACATATTCATGATCCATTAGAGCAAGGGCACCAACCTCTTTCTTGGAATGCAAGGGTTCAGATTGCACTCGATGCAGCAAGAGGTATTGAATACATACATGATCATACAAAAGCTCGATATGTGCATCGCGACATAAAGACTAGCAATATTCTCATTGATGAGAAGCTCAGAGCTAAGGTAGCAGATTTTGGACTTGCAATGCTGGTGGACAGAAGCAATGATGAAAATTT---CATAGCAACAAGGCTTGTTGGAACACCAGGATACCTTCCACCCGAATCTGTGAAGGAGCTTCAGGTGACCCCAAAAACCGATGTGTTTGCATTTGGAGTGGTTCTAGCAGAACTGATAACAGGGAAACGTGCACTGTTTCGGGACAAGAACGA-------AGAAGGCACCAAAATGAAATCACTTATTTCACTTGTACATCAAATATTCCAAGATG---CAGAGCCAGAGAATGGTCTAG------AAGATGTGATAGATAAGAACCTTGAAGCAAACTATCCCATTGAATACGTGTTGAAGGTAGCAGAGATAGCAGGTCGGTGCTTGCAAGAAGATCCAGTAGAAAGGCCTGAAATGAGGGACTTGGTTGGAGCACTGTCACAGATAGTGATGAACTCCATAGAATGGGAAGCATCCCTTGGTGGAAACAGCCAAGTCTTCAGTGGT---GTCTTTACTGGAAGATGA----

>SBbav42xvGysNbOiTKsv+ES1sMc.1632394372404.438590

------------------------------------------------------------------------------------------------------------------------------------------------------------------------------------------------------------ATGAAAA---CAACATACATGGACCCGCTAAGTTGCTCTGTAGAGGCTGC-CAATACATGC--ACTGCCTCACTCTACCACATAACTCATGATGATTACAG------CCTTGAAGAAATTGCAT---------------TTTATTACTCTGTTAACTCT---TCACAAATCAAGCCTATTAAGTAT---------GGAACAAGGCA---AGATTACCTCATAACAGTTCCTTGTTCTTGCAAGAACACACAAAAC------CTCAGCGGGTATTTCTATCATACAACCTACAAAAATGTGAAGCATGGGGACATCTTTGTGGACATATCAGCTTCTGTTTACAGTGGACAAGCCATGCTGATCACCC------AAAGTTTAAT------TCCAGGCAAAGATCTAGAAATAGACATTCCATGTGGGTGTTCCGA---------------GGATGAGGGTCAAAGGGTTG-TCACATATACAGTGC-AGCAGAATGA-TACACCAGAAACAATTTCCCTTTTGCTTAATGCTACCCTGCCTGCCTTGCTGAGAATGAACAAGATTCTAGCTCAGAAACCTGGTTTCATAGATATTGGTTGGGTGCTCTTTGTTCCTTT---GGAATTGAATGGGGTTCATGG-------GGTTCCTCCACAACCCACA--GAAAAAACGAAACGGAA---CTGG---CCAATGATAGTTGGTATCGTCGTGGGAG---TGCTGTTACTTTCAGTTATCATTATCACCATTCTCATA---ATTCTTTGGAGAAGGAGA---GTCCATCAAAT---CACCAGAAATGATTC---CACAGTTGTCTCTAAAAGATCATTTGTCAACAG---------------------------AAA---TATTACCTTGCATAACTCC-ACCC-TTTACAAAGAATATATGGGAGATGTAATGCAAATTGAATCAGAGAGGCCAGTGATATATGGTCTTGAGGAGATAGAAGAGGCTACAAATAACTTTGATGAAGCTCGAAAAATCGGAGTTGGCGGGTATGGGAGTGTTTATTTTGGAATCTTAGAGCAGAAGGAGGTTGCTGTGAAAAAGATGAGGTCTAATAAGACTAAGGAATTCTATGCGGAACTCAAGGCCTTGTGTAAGATCCATCATATCAACATTGTGGAGCTATTAGGATATGCCAGAGGAGAAGATCACCTCTATTTGGTGTATGAGTATGTTTCAAATGGATCTCTCAGTGAACATATTCATGATCCATTAGAGCAAGGGCACCAACCTCTTTCTTGGAATGCAAGGGTTCAGATTGCACTCGATGCAGCAAGAGGTATTGAATACATACATGATCATACAAAAGCTCGATATGTGCATCGCGACATAAAGACTAGCAATATTCTCATTGATGAGAAGCTCAGAGCTAAGGTAGCAGATTTTGGACTTGCAATGCTGGTGGACAGAAGCAATGATGAAAATTT---CATAGCAACAAGGCTTGTTGGAACACCAGGATACCTTCCACCCGAATCTGTGAAGGAGCTTCAGGTGACCCCAAAAACCGATGTGTTTGCATTTGGAGTGGTTCTAGCAGAACTGATAACAGGGAAACGTGCACTGTTTCGGGACAAGAACGA-------AGAAGGCACCAAAATGAAATCACTTATTTCACTTGTACATCAAATATTCCAAGATG---CAGAGCCAGAGAATGGTCTAG------AAGATGTGATAGATAAGAACCTTGAAGCAAACTATCCCATTGAATACGTGTTGAAGGTAGCAGAGATAGCAGGTCGGTGCTTGCAAGAAGATCCAGTAGAAAGGCCTGAAATGAGGGACTTGGTTGGAGCACTGTCACAGATAGTGATGAACTCCATAGAATGGGAAGCATCCCTTGGTGGAAACAGCCAAGTCTTCAGTGGT---GTCTTTACTGGAAGATGA----

>arahy.Tifrunner.gnm2.ann1.P7CPN3.1

---------------------------------------------------------------------------------------------ATGTCTTCTTCTC---TCAATAATCTTCTTTGTCTTCTCTTCTCTCTTTTGGCAACTTC-------ATCTTCT--------ATCAGAGTCTCTGCATTTC---GTGTTTCAATGAAAA---CAACATACATGGACCCGCTAAGTTGCTCTGTAGAGGCTGC-CAATACATGC--ACTGCCTCACTCTACCACATAACTCATGATGATTACAG------CCTTGAAGAAATTGCAT---------------TTTATTACTCTGTTAACTCT---TCACAAATCAAGCCTATTAAGTAT---------GGAACAAGGCA---AGATTACCTCATAACAGTTCCTTGTTCTTGCAAGAACACACAAAAC------CTCAGCGGGTATTTCTATCATACAACCTACAAAAATGTGAAGCATGGGGACATCTTTGTGGACATATCAGCTTCTGTTTACAGTGGACAAGCCATGCTGATCACCC------AAAGTTTAAT------TCCAGGCAAAGATCTAGAAATAGACATTCCATGTGGGTGTTCCGA---------------GGATGAGGGTCAAAGGGTTG-TCACATATACAGTGC-AGCAGAATGA-TACACCAGAAACAATTTCCCTTTTGCTTAATGCTACCCTGCCTGCCTTGCTGAGAATGAACAAGATTCTAGCTCAGAAACCTGGTTTCATAGATATTGGTTGGGTGCTCTTTGTTCCTTT---GGAATTGAATGGGGTTCATGG-------GGTTCCTCCACAACCCCCA--GAAAAAACGAAACGGAA---CTGG---CCAATGATAGTTGGTATCGTCGTGGGAG---TGCTGTTACTTTCAGTTATCATTATCACCATTCTCATA---ATTCTTTGGAGAAGGAGA---GTCCATCAAAT---CACCAGAAATGATTC---CACAGTTGTCTCTAAAAGATCATTTGTCAACAG---------------------------AAA---TATTACCTTGCATAACTCC-ACCC-TTTACAAAGAATATATGGGAGATGTAATGCAAATTGAATCAGAGAGGCCAGTGATATATGGTCTTGAGGAGATAGAAGAGGCTACAAATAACTTTGATGAAGCTCGAAAAATCGGAGTTGGCGGGTATGGGAGTGTTTATTTTGGAATCTTAGAGCAGAAGGAGGTTGCTGTGAAAAAGATGAGGTCTAATAAGACTAAGGAATTCTATGCGGAACTCAAGGCCTTGTGTAAGATCCATCATATCAACATTGTGGAGCTATTAGGATATGCCAGAGGAGAAGATCACCTCTATTTGGTGTATGAGTATGTTTCAAATGGATCTCTCAGTGAACATATTCATGATCCATTAGAGCAAGGGCACCAACCTCTTTCTTGGAATGCAAGGGTTCAGATTGCACTCGATGCAGCAAGAGGTATTGAATACATACATGATCATACAAAAGCTCGATATGTGCATCGCGACATAAAGACTAGCAATATTCTCATTGATGAGAAGCTCAGAGCTAAGGTAGCAGATTTTGGACTTGCAATGCTGGTGGACAGAAGCAATGATGAAAATTT---CATAGCAACAAGGCTTGTTGGAACACCAGGATACCTTCCACCCGAATCTGTGAAGGAGCTTCAGGTGACCCCCAAAACCGATGTGTTTGCATTTGGAGTGGTTCTAGCAGAACTGATAACAGGGAAACGTGCACTGTTTCGGGACAAGAACGA-------AGAAGGCACCAAAATGAAATCACTTATTTCACTTGTACATCAAATATTCCAAGATG---CAGAGCCAGAGAATGGTCTAG------AAGATGTGATAGATAAGAACCTTGAAGCAAACTATCCCATTGAATACGTGTTGAAGGTAGCAGAGATAGCAGGTCGGTGCTTGCAAGAAGATCCAGTAGAAAGGCCTGAAATGAGGGACTTGGTTGGAGCACTGTCACAGATAGTGATGAACTCCATAGAATGGGAAGCATCCCTTGGTGGAAACAGCCAAGTCTTCAGTGGT---GTCTTTACTGGAAGATGA----

>Ae03G06000

---------------------------------------------------------------------------------------------------ATGGCTT---CTCATACTCAGTTTCTTATTCTTCTTTTCCCTCTGCTGACAAC-------TTCATTT--------ATTGGAGTTTTTGCCCTTC---AAGCTTCAATCAACA---CAACAAATATGGCTCCATTTAGTTGCTTTGAAAA---TAT-CACAACTTGT--ACTGCCTCACTCTACCACATAAGCCATA---ATTTCAA------CCTTGAAGAGATTGCCT---------------CCTTTTACTTGGTTAATTCT---TCCCAAATCAAGCCTATAATGCAT---------GGCAAAAAGCA---AGATTACCTCATAACAGTGCCTTGTTCTTGCAAGAACACAAGTGGT------ACTAGTGGATATTTCTATGACACAACCTACAAAGAAGTGAAGCAACATGACACTTTTGTGGGTATATCAAACTTGGTTTACAGTGGACAAGCCTGGCCAGGTAATG------ATAAATTGAA------TCCAGGTGAGGATTTACCAATACACATTCCATGTGGTTGCTCAGA---------------GGATGAGTCTCAAAGAGTTG-TTACCTACACAGTCC-AACCGAGTGA-TACACAAGAAACGATTTCCCTTCTGCTAAATACTACAATTCTTGCAATGCAGAGCATGAACAAAGTTCTGGTTCAGAACCCTGATTTGATACTTTCTGGTTGGGTGCTATATGTTCCTTT---GGAATTAAATGGGGTTC-------------CTCCATCTCCCACTGGA--AAAAAAAAGAAACATAT---GTTG---CCAATAATCATCGGTATCTTAGCAGGCT---TGATGTTGCTTTCAATCATCATAATCACCCTCCTCA------TTGTCAGGAGAAAAAGA---GCCCATCAAGT---CAGCAAAGAAGATTCAGCCATTATTGTCTCTAAAAGATCAATTACCAATAG---------------------------ACA---CATTTCCTTGCAGACTTCT-AACA-TTTATAAAGAATATATGGGAGATGTAATGCAAATTGAATCAGAAAGACCAGTGATATATAGTCTTGAGGAGATTGAAGAGGCTACAAATAACTTTGATGAAGCTAGAAGAATCGGAGTTGGCGGATATGGGAGTGTTTATTTTGGAATACTGGAGCAAAAGGAGATTGCTGTAAAGAAGATGAGGTCTAATAAAACTAAAGAGTTCTTTGCAGAACTCAAGGCCTTGTGTAAGATCCATCATATCAACATCGTTGAGCTGTTGGGATATGCCAGCTGCGAAGATTACCTCTATTTGGTGTATGAGTATGTTCCAAATGGATCTCTCAGTGATCATCTTCATGATCCATTACAGAAAGGCCACCAGCCTCTTTCTTGGAATGCTAGGGTTCAGATTGCACTGGATTCTGCTAGAGGCCTTGAATACATACATGACCATACAAAAGCACGATATGTACACCGTGACATAAAGACTAGCAATATTCTGATTGATGAGAAGCTCAGAGCAAAGGTAGCAGATTTTGGACTTGCAAAACTGGTAGACCGAGCCAATGATGAAAGTTT---TATAGCAACAAGGCTTGTTGGAACACCAGGCTACCTTCCACCTGAAGCTGTGAAGGAGCTTCAGGTGACCCCCAAAACCGATGTGTTTGCATTTGGAGTGGTTTTGTTGGAGCTGATTACAGGGAAACGTGCACTATTTCGTGATAGCCAGGA-------AGCTGGCACCAAAATGAAATCACTTATTTCAGTTGTGCATAAAGTATTCCAAGATG---CTGAGCCAGAGACAGCTCTAG------AAGATATCATAGATCGGAATCTCGAAGCTAACTATCCGATGGAAGATGTCTACAAGATGGCGGAAATAGCTAGGTGGTGCTTGGTAGAAGAGGCAGTGGAAAGGCCTGAAATGAGAGATATCGTTGGGGCATTGTCACGGATTGTTATGTCCTCCATAGAGTGGGAAGCATCATTAGGTGGGAACAGCCAAGTCTTCAGCGGT---TTGTTTGATGGAAGATGA----

>Glyma08G283300

---------------------------------------------------ATGTATTCCACTTCCCTCCTTCACCAGGGGAGTTTCTACTTCACAACTATGATTT---CATTCACTAATCTTCTTTCTCTTCTTTTCCCTTTGTTCACAAC-------TTCTTTT--------GTTAGAGTTTTTGCCTCTG---AGGTCTCAATCAAAA---CAACAAATTTGTCACCTTTAAATTGCTCTTCCAAA---AT-CAGAACATGT--AATGCCTCACTCTACCACATATCCCAAA---ATCTCAC------AATTGAACAAATAGCCT---------------CCTTTTACTCAGTTATTTCT---TCCCAAATCACACCTATAATGCAT---------GGCATCAAACA---AGACTACCTCATCAGGGTACCTTGTTCATGCAAAAACACAAGTGGC------CTTAGCGGATACTTTTATGACACAACCTACAAA---GTGAGGCCAAATGACACTTTTGCAAATATTTCAAACCTGATTTTCAGTGGCCAAGCTTGGCCGGTTAATC------ATACATTGCA------ACCAAATGAGACTTTAGCAATACACATTCCATGTGGATGTTCAGA---------------AAGTAAGTCTCAAGTAGTTG-TTACCTACACGGTCC-AGCCGAACGA-TACACCGATGATGATTGCTAATCTGCTAAATTCTACACTGGCTGACATGCAGAACATGAACAAGGTTCTGGCTCCGAACATCGAATTCATCGATGTTGGTTGGGTGCTATTTGTCCCTAA---GGAATCCAAAGGGCTACTACTACTACCTAGTGCTACTAGTACTAATATTGAAGAAAAGAAACATAATAAGTGGACAACAATAATCATTGGCATCTTAGGGGGCA---TGACATTGCTTTCAATTGTTACAACGATCATTCTCA------TTCTCAGGAGAAATAAA---GTCGACAAAAT---CAGCATAGAAGATTCA--CGC-CTTATTTCTGGAAGATCAATTGCCAATAA---------------------------AAC---TATTTCCTCAAAGTA---T-AGTC-TTCATAAGGAATTTGTGGAAGATTTAATTTCATTTGAATCAGAAAGACCATTGATATATAACCTTGAGGACATTGAAGAGGCTACAAATAACTTTGATGAAAGTCGTAAAATAGGAAGTGGTGGATATGGAAGTGTTTATTTTGGAATCTTAGGGAACAAGGAGGTTGCTGTGAAGAAGATGAGGTCTAATAAATCTAAAGAGTTCTATGCTGAACTCAAGGTCTTGTGTAAAATCCACCATATCAACATTGTGGAGCTGTTGGGATATGCCAACGGAGAAGATTATCTCTATTTAGTGTATGAGTATGTTCCAAATGGATCTCTCAGTGATCATCTTCATAATCCATTACTGAAAGGGAACCAGCCTCTTTCTTGGAGTGCTAGGGTTCAAATTGCACTGGATGCTGCAAAAGGTCTTGAATATATACATGATTATACAAAAGCACGATATGTGCACCGTGATATAAAGACTAGCAATATTCTACTTGATAACAAGTTCAGAGCAAAGGTGGGAGATTTTGGACTTGCAAAGCTGGTAGATAGAACAGATGATGAAAATTT---TATAGCAACTAGGCTTGTAGGAACACCAGGCTACCTTCCACCAGAATCTCTGAAGGAGCTTCAAGTGACCCCGAAAACCGATGTGTTTGCATTTGGAGTGGTGCTTTCAGAGCTGTTAACAGGAAAACGTGCACTTTTTCGAGAAAGCCACGA-------AGA---CATCAAAATGAAATCACTTATTACTGTTGTTAATGAAATATTCCAAGATG---ATGACCCAGAGACTGCTTTAG------AAGATGCCATAGATAAGAATCTTGAAGCTAGCTATCCGATGGAAGATGTCTACAAGATGACAGAAATAGCTGAGTGGTGCTTGCAAGAAGATCCAATGGAAAGGCCTGAAATGAGGGATATCATTGGGGCATTGTCACAGATTGTGATGTCCTCGACAGAGTGGGAAGCATCCCTATGTGGAAACAGCCAGGTCTTTAGTGGG---TTATTTAGTGGAAGATGA----

>Glysoja.08G021937

---------------------------------------------------ATGTATTCCACTTCCCTCCTTCACCAGGGGAGTTTCTACTTCACAACTATGATTT---CATTCACTAATCTTCTTTCTCTTCTTTTCCCTTTGTTCACAAC-------TTCTTTT--------GTTAGAGTTTTTGCCTCTG---AGGTCTCAATCAAAA---CAACAAATTTGTCACCTTTAAATTGCTCTTCCAAG---AT-CAGAACATGT--AATGCCTCACTCTACCACATATCCCAAA---ATCTCAC------AATTGAACAAATAGCCT---------------CCTTTTACTCAGTTATTTCT---TCCCAAATCACACCTATAATGCAT---------GGCATCAAACA---AGACTACCTCATCAGGGTACCTTGTTCATGCAAAAACACAAGTGGC------CTTAGCGGATACTTTTATGACACAACCTACAAA---GTGAGGCCAAATGACACTTTTGCAAATATTTCAAACCTGATTTTCAGTGGCCAAGTTTGGCCGGTTAATC------ATACATTGCA------ACCAAATGAGACTTTAGCAATACACATTCCATGTGGATGTTCAGA---------------AAGTAAGTCTCAAGTAGTTG-TTACCTACACGGTCC-AGCCGAACGA-TACACCGATGATGATTGCTAATCTGCTAAATTCTACACTGGCTGACATGCAGAACATGAACAAGGTTCTGGCTCCGAACATCGAATTCATCGATGTTGGTTGGGTGCTATTTGTCCCTAA---GGAATCCAAAGGGCTACTACTACTACCTAGTGCTACTAGTACTAATATTGAAGAAAAGAAACATAATAAGTGGACAACAATAATCATTGGCATCTTAGGGGGCA---TGACATTGCTTTCAATTGTTACAACGATCATTCTCA------TTCTCAGGAGAAATAAA---GTCGACAAAAT---CAGCATAGAAGATTCA--CGC-CTTATTTCTGGAAGATCAATTGCCAATAA---------------------------AAC---TATTTCCTCAAAGTA---T-AGTC-TTCATAAGGAATTTGTGGAAGATTTAATTTCATTTGAATCAGAAAGACCATTGATATATAACCTTGAGGACATTGAAGAGGCTACAAATAACTTTGATGAAAGTCGTAAAATAGGAAGTGGTGGATATGGAAGTGTTTATTTTGGAATCTTAGGGAACAAGGAGGTTGCTGTGAAGAAGATGAGGTCTAATAAATCTAAAGAGTTCTATGCTGAACTCAAGGTCTTGTGTAAAATCCACCATATCAACATTGTGGAGCTGTTGGGATATGCCAACGGAGAAGATTATCTCTATTTAGTGTATGAGTATGTTCCAAATGGATCTCTCAGTGATCATCTTCATAATCCATTACTGAAAGGGAACCAGCCTCTTTCTTGGAGTGCTAGGGTTCAAATTGCACTGGATGCTGCAAAAGGTCTTGAATATATACATGATTATACAAAAGCACGATATGTGCATCGTGATATAAAGACTAGCAATATTCTACTTGATAACAAGTTCAGAGCAAAGGTGGGAGATTTTGGACTTGCAAAGCTGGTAGATAGAACAGATGATGAAAATTT---TATAGCAACTAGGCTTGTAGGAACACCAGGCTACCTTCCACCAGAATCTCTGAAGGAGCTTCAAGTGACCCCGAAAACCGATGTGTTTGCATTTGGAGTGGTGCTTTCAGAGCTGTTAACAGGAAAACGTGCACTTTTTCGAGAAAGCCACGA-------AGA---CATCAAAATGAAATCACTTATTACTGTTGTTAATGAAATATTCCAAGATG---ATGACCCAGAGACTGCTATAG------AAGATGCCATAGATAAGAATCTTGAAGCTAGCTATCCGATGGAAGATGTCTACAAGATGACAGAAATAGCTGAGTGGTGCTTGCAAGAAGATCCAATGGAAAGGCCTGAAATGAGGGATATCATTGGGGCATTGTCACAGATTGTGATGTCCTCGACAGAGTGGGAAGCATCCCTATGTGGAAACAGCCAGGTCTTTAGTGGG---TTATTTAGTGGAAGATGA----

>LOC106780768_CDS

------------------------------------------------ATGTTTTCCTTATTCTCTCTCCTTCACCAGGGGAGTTTCTACATCACAACTATGGCTT---CTCTCACTCCTCTTCTTCCATTTCTTTTCCCTTTTCTGGCAAC-------TTCTTTT--------GTCACAGTTTTTTCCTTTC---AGGTCTCAATCAAAC---CTACAAATTTGTCTCCTTTAAGTTGCTCTGACAAG---AT-CCTAACATGT--AATGCCTCCCTCTACCACATATCCCATA---ATCTCAC------CATTCAGCAAATTGCCT---------------CTTTTTACTCGGTTACTTCT---TCCCATGTCACACCTATAAAACAT---------GGCACCAAAGA---AGACTACCTCATCACAGTACCTTGTTCATGCAAACATACGAGTGAC------CTTAGCGGATATTTCTATGACACCACCTACAAA---GTAAAGCTACATGACACTTTTGTGAATATTTCAAACCTGGTTTTCAGTGGCCAAGCTTGGCCAGTTAATG------GTACATTGCA------TCCAGATGAGAATTTAGCAATACACCTTCCATGTGGGTGCTCAGA---------------AAGTGACTCTCAAATTGTTG-TCACCTATACAGTTC-AGCCAGATGA-TACACCAACAGTGATTGCTAATCTGCTAAATGCTTCACTGGTTGACATGATGAGCATGAACAAGGTTTTGGCTCCGAACTTTAACTTCATAGATGTTGGTTGGGTTCTGTTTGTTCCCAA---GGGATCCAAAGGGCTATTAC------CGGGTACTGCTG---CTGTCAATGAA---AAGAAACTTAA---GTGGACAACAACAATAATTGGTATCTTAGCGGGAG---TGACATTCCTTTCGGTTACTACAACCATCATTCTCA------TTGTCAGGGTAAATAAA---GTTAATCAAAT---GATCGGTGAAGATTCA--CAC-CTTATCTCCAGAAGATCAATGGCCAATAG---------------------------AAC---TATTTCCTCAAAGTT---T-AACT-TTCAAAAGGAATCTATAGAAGATGTAATTTCATTAGAATCAGAAAGACCAATAGTATACAACCTTGAGGAGATTGAAGAGGCTACGAATAACTTTGATGAAAGTAGAAAAATAGGAAGTGGTGGATACGGAAGTGTTTATTTTGGAGTGTTAGGGAACAAGGAAGTTGCTGTGAAGAAAATGAGGTCCAATAAATCTAAAGAGTTCTATGCAGAACTCAAGGTCTTATGTAAGATCCACCATATCAACATTGTGGAGCTATTGGGATATGCAAACGGAGAAGATGACCTCTATTTGGTGTATGAGTACGTTCCAAATGGATCTCTCAGTAATCATCTTCATAATCCATTACTGAAAGGGAACCAGCCTCTTTCTTGGAGTGTTAGGGTTCAAATTGCACTGGATGCTGCAAAAGGTCTTGAATATATACATGATTATACAAAAGCACGATATGTGCACCGTGACATAAAGAGTAGCAATATTCTACTTGATGACAAATTCAGAGCAAAGGTGGGAGATTTTGGACTTGCAAAGCTTGTAGACAGAACCGATGATGAAAATTT---TATAACAACCAGACTTGTAGGAACACCAGGCTACCTTCCACCAGAGTCTCTGAAGGAGCTTCAAGTGACCCCAAAAACCGATGTTTTTGCGTTTGGAGTGGTGCTTTCAGAGCTGTTAACAGGGAAACGAGCATTATTCCGAGAAAGCCAAGA-------AGA---GATTAAAATGAAATCACTCATTAGTGTTGTTAATACAATATTCCAAGATG---ATGAACCAGAGATTGCCTTAG------AAGATGCCATAGATAAGAATCTGGAAGCAAGCTATCGGATGGAAGATGTCTACAAGATGGCAGAAATAGCTGAGTGGTGCTTGCAAGAAGATCCAATAGAGAGGCCTGAAATGAGGGATATAATTGGGGCACTGTCTCAGATTGTGATGTCCTCAATAGAGTGGGAAGCATCCCTGTGTGGAAATAGCCAGGTCTTCAGCGGG---CTGTACAGTGGAAGATGA----

>Vigun03g232900

---------------------------------------------------------------------------------------------------ATGGCTT---CTCTCACTCCTCTTCTTCCATTTCTTTTCCCTTTTCTGGCAAC-------TTCTTTT--------GTCACAGTTTTTGCCTTTC---AGGTCTCAATCAAAC---CTTATAATTTGTCTCCTTTAAATTGCTCTGCTAAG---AT-C---ACATGT--AATGCCTCCCTCTACCACATATCTCATA---ATCTCAC------CATTCAGCAAATTGCCT---------------CTTTTTACTCCGTTACTTCT---TCCCATATCACACCTATAAAACAT---------GGCACCAAACA---AGACTACCTCGTCACAGTACCTTGTTCATGCAAACATACGAGTGAC------CTTAGCGGATATTTCTATGACACCATCTACAAA---GTAAGGCCACGTGACACTTTTGTGAATATTTCAAACCTGGTTTTCAGTGGCCAAGCTTGGCCAGTTAATG------GTACATTTCA------CCCAGATGAGAATTTAGCAATACACCTTCCGTGTGGGTGCTCAGA---------------AAGTGGCTCTCAAATAATTG-TCACCTATACAGTTC-AGCCAGATGA-TACACCAACACTGATTGCTGATCTGCTAAATGCTTCACTGGTTGACATGATGAGCATGAACAACGTTCTAACTCCGAACTTTAAATTCATAGATGCTGGTTGGGTTCTATTTGTTCCCAA---GGGATCCAAAGGGGTATTAC------CCAGTACTGCTG---CTGAAAATGAA---AAGAAACTTAA---GTGGACAACAACAATAATTGGCATCTTAGCGGGAG---TGACATTGCTTTCAGTTACTACAACCATCATTCTCT------TTGTCAGGGTAAATAAA---GTGACTCAAAT---GAGCAGTGAAGATTCA--CAC-CTTTTCTCTAGAAGATCAATGGCCAATAG---------------------------AAC---TATTTCCTCAAAGTA---T-AACT-TTCAAAAGGAATATATAGAAGATGTAATTTCATTAGAATCAGAAAGACCAATAGTATACAACCTTGAGGATATTGAAGAGGCTACGAATAACTTTGATGAAAGTCGAAGAATAGGAAGTGGTGGATATGGAAGTGTTTATTTTGGAGTGTTAGGGAACAAGGAAGTTGCTGTGAAGAAAATGAGGTCCAATAAATCTAAAGAGTTCTATGCAGAACTCAAGGTCTTGTGTAAGATCCACCATATCAACATTGTGGAGCTGTTGGGATATGCAAACGGAGAAGATGACCTCTATTTGGTGTATGAGTACGTTCCAAATGGATCTCTCAGTGATCATCTTCATGACCCATTACTGAAAGGGAACCAGCCTCTTTCTTGGAGTGTTAGGGTTCAAATTGCACTGGATGCTGCAAAAGGTCTTGAATATATACATGACTATACAAAAGCACGATATGTGCACCGTGACATAAAGAGTAGCAATATTCTACTTAATGACAAGTTCAGAGCAAAGGTGGGAGATTTTGGACTTGCAAAGCTTGTAGACAGAACCGATGATGAAAATTT---TATAGCAACCAGACTTGTAGGAACACCAGGCTACCTTCCACCAGAGTCTCTGAAGGAGCTTCAAGTGACCCCAAAAACCGATGTGTTTGCGTTTGGAGTGGTGCTTTCAGAGCTGTTAACAGGGAAACGAGCATTATTTCGGGAAAGCAAAGA-------AGA---GATTAAAATGAAATCACTCATTAGTGTTGTTAATAAAATATTCCAAGATG---ATGAACCTGAGATTGCCTTAG------AAGATGCCATAGATAAGAATCTTGAAGCAAGCTATCGGATGGAAGATGTCTACAAGATGGCAGAAATAGCTGAGTGGTGCTTGGAAGAAGATCCAATAGAGAGGCCTGAAATGAGGGATATAATTGGGGCACTGTCTCAGATTGTGATGTCCACAATAGAGTGGGAAGCATCCCTATGTGGAAATAGCCAGGTCTTCAGCGGG---TTGTACAGTGGAAGATGA----

>PhvuI.003G063700_CDS

---------------------------------------------------------------------------------------------------ATGGCTT---CTTTCACTCCTCTGCTTGCATTTGTTTTCCCTTTGCTGACAAC-------TTTTCCT-----------ACAGTTTTTGCCTTTG---AGGTCTCAATCAAAG---CTACTAATTTGTCTCCTTTATATTGCTCTGCTAAG---AT-CACAACATGT--AATGCCTCACTCTACCACACATCCCATA---ATCTCAC------CATTCACCAAATTTCCT---------------CCTTTTACTCGGTTACTTCT---TCCCATATCACACCCATAAAGCAT---------GGCACCAAACA---AGACTACCTCCTCAGAGTACCCTGTTCTTGCAAACATACAAGTGAC------CTTAGCGGATATTTCTATGACACCACCTACAAA---GTAAGGCCACATGACACTTTTTCCAATATTTCAAACCTGGTTTTCAGTGGCCAAGCTTGGCCAGTTAATG------GTACATTGCA------CCCAGATGAGAATTTAGCAATACACATTCCATGTGGGTGCTCAGA---------------AAGTGACTCTCAAATAGTTG-TCACCTATACAGTTC-AGCCAAATGA-TACACCAACACTGATCGCGAATCTGCTAAATGCTTCACTGGCTGACATGCTGAGCATGAACAAGATTCTGGATCCGAACTTTAAATTCATAGATGTTGGTTGGGTGCTATTTGTTCCCAA---AGGATCCAAAGGGCTATTAC------CTAGTACTGCTG---CTGGCAATGAAGAAAAGAAACTTAA---GTGGGCAACAACAATAATTGGTATCTTAGCGGGAG---TGACATTCCTTTCAGTTATTACAACTATCATTCTCA------TTGTCAGAGTAAATAAA---GAGAATCAAAA---GAACAGTGAAGATTCA--CGC-CTTATCTCTAGAAGATCAATTGCCAATAG---------------------------AAC---TATTTCCTCAAAGTA---T-AACT-TTCAAAAAGAATATATAGAAGATGTTGTTTCATTAGAATCAGAAAGACCAATAGCATATAATCTTGAGGTGATTGAAGAGGCTACGAATAACTTTGATGAAAGTCGAAGAATAGGAAGTGGTGGATATGGAACTGTTTATTATGGAGTGTTAGGGAACAAGGAGGTGGCTGTGAAGAAAATGAGGTCCAATAAATCTAAAGAGTTCTATGCAGAACTCAAGGTCTTATGCAAGATCCACCATATCAACATTGTGGAGCTGTTGGGATATGCCAACGGAGAAGATGACCTCTATTTGGTGTATGAGTACGTTCCAAATGGATCTCTCAGTGATCATCTTCATGACCCATTACTGAAAGGGAACCAGCCTCTTTCTTGGAGTGTTAGGGTTCAAATTGCACTGGATGCTGCAAAAGGTCTTGAATATATACATGACTATACAAAAGCACGCTATGTGCACCGTGACATAAAGAGTAGCAACATTCTACTTAATGACAAGTTCAGAGCAAAGGTGGGAGATTTTGGACTTGCAAAGCTAGTAGATCGAACCGATGATGAAAATTT---TATAGCAACCAGGCTTGTAGGAACACCAGGCTACCTTCCACCAGAATCTCTGAAGGAACTTCAAGTGACCCCAAAAACCGATGTGTTTGCGTTTGGAGTGGTGCTTTCAGAGCTGTTAACAGGGAAACGAGCATTATTTCGAGAAAGCCAAGA-------AGA---GATTAAAATGAAATCACTCATCAGTGTTGTTAATAAAATATTCCAAGATG---ATGACCCAGAGATTGCCTTAG------AAGATGCCATAGATAAGAATCTTGAAGCAAGCTATCGGATGGAAGATGTCTACAAGATGGCAGAAATAGCTGAGTGGTGCTTGCAAGAAGATCCAATAGAAAGGCCTGAAATGAGGGATATAATTGGGGCACTGTCACAGATTGTGATGTCCTCAACAGAGTGGGAAGCATCCCTATGTGGAAATAGCCAGGTCTTCAGCGGG---CTGTACAGTGGAAGATGA----

>Macrotyloma_uniflorum_EPR3_CDS

---------------------------------------------------------------------------------------------------ATGGCTT---CTTTCACTCAGCTTCTTCCATTCCTTTTCCCTTTGCTAGCAAC-------TTCTTTT--------GTTACAGTTTTTGCCTTTG---AGGTCTCAATCAAAG---ATACTAATTTGTCTCCTTTAAATTGCTCTGCTAAG---AT-CAGAATATGT--AATGCCGCACTCTACCACATATCCCAAA---ATCTCAC------CATTGAGCAAATTGCCA---------------CCTTTTACTCGGTTATTTCT---TCTCAAATCACACCTATAATGCAT---------GGCAGCAAACA---AGACTACCTCATCACAGTACCTTGTTCATGCAAACATACAATTGAC------CTTAGCGGATATTTCTATGACACAACCTACAAA---GTAAGGCCACATGACACTTTTCTGAATATTTCAAACCTGGTTTTCAGTGGCCAAGCTTGGCCAGTAAATG------GTACATTGCA------CCCAGATGAGAATTTAGCAATAAATATTCCATGTGGGTGCTCAGA---------------AAGTGATTCTCAAATAGTTG-TCACCTACACAGTTC-AGCCACATGA-TACTCCAACAGTGATTGCTAATCTGCTAAATGCTTCACTGGCTGAGATGCTGAGCATGAACAAGGTTCTGGCTCAGAACCTTGAATTCATAGATGTTGGTTGGGTGCTATTTGTTCCCAG---TGGATCCAAAGGGCTACTAG------CTAGTTCTGC------TGACAATGAAGAAAAGAAACATAA---GTGGACAACAACAATAATTGGTATTTTAGCGGGAG---TGACATTGCTTTCAGTTATTACAACCACCATTCTCA------TTCTCACCAGGAATAAA---GTGAATCAAAC---GAGCAGTGAAGCTTCA--CGA-CTTATGTCTAGAAGATCAATTGCCAATAG---------------------------AAC---TATTTCCTCAAAGTA---T-AACT-TTCAAAAGGAATATATAGAAGATGTAATTTCAATAGAATCAGAAAGACCAATAGTATACAACTTTGAGGAGATTGAAGAGGCTACAAATAACTTTGATGAAAGTAGAAGAATAGGAAGTGGTGGATATGGAAGTGTTTATTTTGGAGTGTTAGGGAGCAAGGAGGTTGCTGTGAAAAAGATGAGGTCAAATAAATCTAAAGAGTTCTATGCAGAACTCAAGGTCTTATGTAAGATCCACCATATCAACATTGTGGAGCTATTGGGATATGCCAACGGAGAAGATGACCTCTATTTGGTGTATGAGTACGTTCCAAACGGATCTCTCAGTGATCATCTTCATGATCCATTACTGAAAGGGAACCTGCCACTTTCTTGGAGTGTTAGGGTTCAAATTGCACTGGATGCTGCAAAAGGTCTTGAATATATACATGACTATACAAAAGCACGATATGTGCACCGTGACATAAAGAGTAGCAATATTCTACTTAATGACAAGTTCAGAGCAAAGGTGGGAGATTTTGGACTTGCAAAGCTGGTAGATCGAACAGATGATGAACATTT---TATAGCAACCAGGCTTGTAGGGACACCAGGCTACCTTCCACCAGAATCTCTGAAGGAGCTTCAAGTCACCCCAAAAACTGATGTGTTTGCGTTTGGAGTGGTACTTTCAGAGCTGTTAACAGGGAAACGTGCATTATTTCGAGAAAGCCAAGA-------AGA---TATCAAAATGAAATCACTCATTAGTGTTGTTAATAAAATATTTCAAGATG---ATGATCCAGAGATTGCCTTAG------AAGATGCCATAGATAGGAATCTTGAAGCTAGCTATCCAATGGAAGATGTCTGCAAGATGGCAGAAATAGCTGTGTGGTGCTTGCAGGAAGATCCAGTAGAAAGGCCTGAAATGAGGGATATAATTGGGGCACTGTCACATATTGTGATGTCCTCAACAGAGTGGGAAGCATCACTATGTGGAAATAGCCAGGTCTTCAGTGGG---GTGTATAGTGGAAGATGA----

>CM014733.1_CDS

---------------------------------------------------------------------------------------------------ATGGCTT---CTTTCACTCAGCTTCTTTCTCTTCTTTTCCCTTTGCTGGCAAC-------TTCTCTT--------GTTGGAGATTTTGCCTTTG---AAGTCTCAATCAAAA---CAACTTATTTGGCTCCTTTAAATTGCTCTGCTAAG---AT-CAGGACATGC--AATGCCTCACTCTACCACATAACCCAAA---ATCTCAC------GACTGAACAAATTGCCT---------------CTTTTTACTCGGTTATTTCT---TCCCAAATCACACCTATAATGCAT---------GGCACCAAACA---AGACTACCTCATCTCGGTACCTTGTTCATGCAGAAACACCAGTGACTA---CCTTGGCGGATATTTCTATGACACAACCTACAAA---GTGAGGCCAAATGACGCTTTTGTGAATATTTCAAACCTGATTTTCAGTGGGCAAGCTTGGCCAGTTAATGTTGGTGATACATTGCA------TCCAGATGATAATTTAACAATACATATTCCATGTGGGTGCTCAGA---------------AAGTGACTCTCAAATAGTTA-TTACCTATACAGTCC-AGCAGCATGA-TACAACAACAATGATTGCAAAGCTGCTAAATGCTACACTGGCTGGCATGCTGAGCATGAACAAGGTTCTGGCTCCGAACCCTGAATTTATAGATGTTGGTTGGGTGCTATTTGTTCCCAA---GGAAATGAAGGAATCCAAAGGG----CTTCTAC--CTAGTGCTGACAAAGAAGAAAAGAAACATAA---GTGGACAACAACAATCATTGGCATCTTAGCGGGCG---TGACATTACTTTCAATTACTACAACGATCATTCTCA------TTCTCAGGAGAAACAAA---GTCAATCAAAT---CACCAGAGAAGATTCA--CGC-CTTATCTCTAGAAGATCAATCGCCAATAG---------------------------AAC---TATTTCCTCAAAGTA---T-AGCC-TTCGTAATGATTATATAGAAGACGTAATTTCATTTGAATCAGAAAGACCAGTAATATATAACCTTGAGGAGATTGAAGAGGCTACAAATAACTTTGATGAAACTCGAAGAATAGGAAGTGGTGGATATGGAAGTGTTTATTTTGGAATTTTAGGGAACAAGGAGGTTGCTGTGAAGAAGATGGGGTCTAATAAATCTGAAGAGTTCTGTGCAGAACTCAAAGTCTTGTGTAGGATCCACCATATCAACATTGTAGAGCTATTGGGATATGCCAACGGAGAAGATGACCTCTATTTGGTGTATGAGTACGTTCCAAATAGATCTCTTAGTGATCATCTTCATGATCCATTACTGAAAGGCAACCAACCTCTCTCTTGGAGTGCTAGGGTTCAAATTGCCCTGGATGTTGCAAAAGGTCTTGAATATATACATGATTATACAAAGGCACAATATGTGCACCGTGATATAAAGACTAGCAATATTCTACTTGACAACAAGCTCAGAGCGAAGGTGGGAGATTTTGGACTTGCAAAGCTGGTAGATCGAACCAATGATAAAAAATT---TATAGCAACTAGGCTTGTAGGGACACCAGGCTACCTTCCACCAGAATCTTTGAAGGAGTTTCAAGTGACTCCAAAAATTGATGTGTTTGCGTTTGGAGTGGTACTTTCAGAGCTGTTAACAGGGAAACGTGCACTATTTCACGAAAGCCAAGA-------AGA---CATCAAAATGAAATCACTTGTTACTGTTGTTAATAAAATATTCCAAGATG---ATGACCCAGATACTGCTTTAG------AAGATGCCATAGATAAGAATCTTGAAGCTAGCTATCTGATGGAGGATGTCTACAAGATGGCAGCAATAGCTGAGTGGTGCTTGCAAGAAGATCCAATGGAAAGGCCAGAAATGAGGGATATAATTGGAGCATTGTCACATATTATGATGTCCTGGACAGAGTGGGAAGCATCCCTATGTGAAAACAGCGAGGTCTTCAGTGGG---CTGTATAGTGGAAGATGA----

>Lj0g3v0307069

---------------------------------------------------------------------------------------------------ATGGCTT---CTTTCACTCAGCTTTTTTCTTTTCTTTTCCCTCTGTTGGCGAC-------TTCATTA--------CATAGAATTTCAGCCTTTG---AAGTTTCCATAAAAA---CGTCTGTTATAGCTCCTCTTAATTGCTCTGCCAAT---AT-CAAAACATGT--AATGCCTTCCTCTACCACATAAGCCACA---ATCACAC------AGTTGAAGAAATTGCCA---------------CCTTTTACTCCGTTAGTTCT---TCCCAAATCAATCCTATAATGTAT---------AGAACCAGACA---AGATTACCTCATAACAGTACCTTGTTCTTGCAAAATAACCAATGAA------CTTAGCGGATATTTCTATGATACAACCTACACA---GTCAGGCCAAATGACATTTTCTCGAATATTTCAGACCTGATTTACAGTGGCCAAGCCTGGCCAGTTACCG------GTACGTTGTT------TCCGGAAGAGCATTTATCGATACATATTCCATGTGGGTGTTCAGA---------------AAATGAGTCTCAAATAGTTG-TCACCTACACAGTTC-AGCAGAATGA-TACACAAACAACTATTGCTAATCTGTTAAATTCTACACGGGCTGGCATACTGAGCATGAACATAATTCTAGATAAGAACACTGGTTTCATAGACCTTGGTTGGGTGCTGTTTGTTCCCAA---GGAATTGAAAGGGCTTCAA------------------------------------AAGAAACATAAGAACTGG---ATAATCATCATTGGCATCTTAGCGGGCG---TGGTATTACTTTCAATTATTGCAATCATCATTGTCA------TTCTCAGGAGAAAAGGA---GCCAATCAAAT---CAGCAAAGAAGATTCA--CAA-GTTGTCTCTAAAAGATCAATTCGCAGTAG---------------------------AAA---ATTCTCCTCAATGAACAGT-AACC-TTCATACGCGATACATTTCTGATGCAATGTCAATAGAATCAGAAAGACCAGTAGTGTACAACCTTGATGACATTGAAGAGGCTACAAATTACTTTGATGAAACTCAAAAAATTGGAAGGGGTGGATTTGGGAGTGTGTATTTCGGAATGTTAGGGAAGACGGAGGTTGCTGTGAAGAAGATGAGGTCTAATAAATCTAGAGAGTTCTATGCAGAACTCAAGGTGTTGTGTAGGATCAATCATATCAACATTGTGGAGCTGTTGGGATATGCCAGTGGAGAAGATCACCTCTATTTGGTGTATGAGTATGTATCAAATGGATCTCTTAGTGACCATCTTCATGATCCATTACTAAAAGGTTACCAGCCTCTTTCATGGAGTGCTAGGGTTCAGATTGCACTTGATGCTGCAAAAGGTCTTGAATATATACATGATCATACGAAAGCACGATATGTTCACCGAGATATAAAGACTAGCAATATCCTTCTTGACGAAAAGCTCAGAGCTAAGGTAGGAGATTTTGGACTTGCAAAGCTAGTAGACAGAACCAATGATGAAAGTTT---TATAGCAACAAGGCTTGTTGGAACACCAGGCTACCTTCCACCAGAATCTGTGAAGGAGCTTCAGGTGACCCCAAAAACTGATGTCTTTGCATTTGGAGTGGTTTTGTCAGAGCTATTAACAGGAAAACGTGCACTATTCCGAGAGAGCAAAGA-------AGC---CACCAAAATGAACTCACTTATTACTGTTGTTAATAAAGTATTTCAAGATG---ATGACCCGATGATTGCTTTAG------AAGATGTCATAGATAATAATCTCGAAGCCAGCTATCCAATGGAAGATGTCTACAAGATAGCAGAAATAGCAGAGTGGTGCTTACAAGAAGATCCAATGGAAAGGCCTGAAATGAGAGATGTCGTTAGGGCATTGACCCAAATTGTGATGTCCTCGATAGAGTGGGAAGCATCACTAGGCGGAAACAGTGAAGTCTTTAGCGGT---TTGTATAGTGGAAGATGA----

>Glyma01G027100_

---------------------------------------------------------------------------------------------------ATGGCTTCTTCTCTAGTGGCACTTCTTTCTCTTCTCCTCACTCTTCTTGCAAC-------TTCATGTCTAAG--TGCAACTGTGTTTTCCCTCC---AAGTTTCAATCAAAA---CAACTTACTTGGAACCTTTCAAATGCTCCCCTAAG---AT-CACCACATGC--AATGCCTCACTCTACCACATAAGCTACG---GCAACAA------CATAGACGACATAGCAA---------------CCTTTTACTCCGTTAGTACC---TCCCAAATCAAACCGATAATGCGC---------ACTACCGAGCA---AGATTACCTCATAACAGTGCCTTGCTCTTGCAATGACACCAATGG------ACTTGGCGGATATTTCTATGATACAACCTACAAG---GTGAAGTCCAATGACACGTTTGTGAATATTAACAACTTTGTTTACAGTGGTCAAGCCTGGCCTATTAACG------GAGAATTGGA------CCAAAATGAGGAGTTAACAATACATCTTCCATGTGGGTGTTCAGAGAA------------AAGTGATTCTCAAATTGTTG-TCACGTACACGGTTC-AGCGGAATGA-TACACCTGTATCAATTGCTGCTCTGCTAAATGCTACGTTAGATGACATGGTGAGTATGAACGAAGTTCTGGCTCAGAACCCCTCATTCATAGATGTTACTTGGGTGTTGTATGTTCCTAG---GGAATTGAATGGTTTACCACT------------------TTCCAAAGGAAAAGACAAGAAACAAAA---GTTG---GAGATAATCATTGGCATCTTAGCGGGTG---TGACATTACTTTCAATTATTACCTTGATTATTCTCAGTGTCGTTCTTAGGAGATCCAGA---GCCAATAAAAC---TGCCAAAAATGATCCA---AGTGTTGTCTCTAAAAGATCAATCACCAATAG---------------------------AAC---TATTTCCATAAAGAACCGA-GACT-TTCATACAGAATATATTGAAGATGCAACAACATTTGAATCAGAAAGACCAGTAATTTATGCTCTAGAGGAGATTGAAGATGCTACAAATAACTTTGATGAAACCCGAAGGATTGGAGTAGGTGGATACGGTACGGTGTATTTCGGAATGTTAGAGGAGAAGGAGGTTGCTGTGAAGAAGATGAGGTCTAATAAATCCAAAGAATTCTATGCAGAACTCAAGGCCTTGTGCAGGATACATCATATTAACATTGTGGAGTTGTTGGGATATGCTAGTGGAGATGACCACCTTTATTTGGTGTATGAGTTTGTTCCAAATGGATCTCTCTGTGAACATCTTCATGATCCATTACTGAAAGGTCACCAGCCACTGTCTTGGTGTGCTAGGATTCAAATTGCACTGGATGCAGCAAAAGGTCTTGAATATATACATGATTACACAAAAGCACGATATGTGCACCGTGATATAAAGACCAGTAATATTCTTCTTGATGAGAAGCTCAGAGCCAAGGTAGCAGATTTCGGACTTGCAAAGCTAGTAGAACGAACCAACGATGAAGAATT---GATAGCGACTAGGCTTGTTGGAACACCAGGCTACCTTCCCCCAGAATCTGTGAAGGAGCTTCAAGTGACTATAAAAACCGATGTATTTGCATTTGGTGTGGTTCTGGCAGAGTTGATAACAGGGAAACGTGCACTATTTCGTGACAACCAAGA-------AGC---CAGCAATATGAAATCACTTACTTCAGTTGTTGGCCAAATATTCAAAGATG---ATGACCCAGAGACTGTTTTAG------CAGATGCCATAGATGGGAATCTTCAACGTAGCTATCCTATGGAAGATGTCTACAAGATGGCAGAACTAGCTCATTGGTGCTTGTGCGAAGATCCAAATGTCAGGCCTGAGATGAGGGAGATTGTTGTGGCATTGTCACAGATTGTGATGTCCTCCACAGAATGGGAAGCATCACTAGGTGGAGACAGAGAGGTCTTCAGCGGG---GTACTTGATGGAAGATGA----

>Glysoja.01G000269

---------------------------------------------------------------------------------------------------ATGGCTTCTTCTCTAGTGGCACTTCTTTCTCTTCTCCTCACTCTTCTTGCAAC-------TTCATGTCTAAG--TGCAACTGTGTTTTCCCTCC---AAGTTTCAATCAAAA---CAACTTACTTGGAACCTTTCAAATGCTCCCCTAAG---AT-CACCACATGC--AATGCCTCACTCTACCACATAAGCTACG---GCAACAA------CATAGACGACATAGCAA---------------CCTTTTACTCCGTTAGTACC---TCCCAAATCAAACCGATAATGCGC---------ACTACCGAGCA---AGATTACCTCATAACAGTGCCTTGCTCTTGCAATGACACCAATGG------ACTTGGCGGATATTTCTATGATACAACCTACAAG---GTGAAGTCCAATGACACGTTTGTGAATATTAACAACTTTGTTTACAGTGGTCAAGCCTGGCCTATTAACG------GAGAATTGGA------CCAAAATGAGGAGTTAACAATACATCTTCCATGTGGGTGTTCAGAGAA------------AAGTGATTCTCAAATTGTTG-TCACGTACACGGTTC-AGCGGAATGA-TACACCTGTATCAATTGCTGCTCTGCTAAATGCTACGTTAGATGACATGGTGAGTATGAACGAAGTTCTGGCTCAGAACCCCTCATTCATAGATGTTACTTGGGTGTTGTATGTTCCTAG---GGAATTGAATGGTTTGCCACT------------------TTCCAAAGGAAAAGACAAGAAACAAAA---GTTG---GAGATAATCATTGGCATCTTAGCGGGTG---TGACATTACTTTCAATTATTACCTTGATTATTCTCAGTGTCGTTCTTAGGAGATCCAGA---GCCAATAAAAC---TGCCAAAAATGATCCA---AGTGTTGTCTCTAAAAGATCAATCACCAATAG---------------------------AAC---TATTTCCATAAAGAACCGA-GACT-TTCATACAGAATATATTGAAGATGCAACAACATTTGAATCAGAAAGACCAGTAATTTATGCTCTAGAGGAGATTGAAGATGCTACAAATAACTTTGATGAAACCCGAAGGATTGGAGTAGGTGGATACGGTACGGTGTATTTCGGAATGTTAGAGGAGAAGGAGGTTGCTGTGAAGAAGATGAGGTCTAATAAATCCAAAGAATTCTATGCAGAACTCAAGGCCTTGTGCAGGATACATCATATTAACATTGTGGAGTTGTTGGGATATGCTAGTGGAGATGACCACCTTTATTTGGTGTATGAGTTTGTTCCAAATGGATCTCTCTGTGAACATCTTCATGATCCATTACTGAAAGGTCACCAGCCACTGTCTTGGTGTGCTAGGATTCAAATTGCACTGGATGCAGCAAAAGGTCTTGAATATATACATGATTACACAAAAGCACGATATGTGCACCGTGATATAAAGACCAGTAATATTCTTCTTGATGAGAAGCTCAGAGCCAAGGTAGCAGATTTCGGACTTGCAAAGCTAGTAGAACGAACCAACGATGAAGAATT---GATAGCGACTAGGCTTGTTGGAACACCAGGCTACCTTCCCCCAGAATCTGTGAAGGAGCTTCAAGTGACTATAAAAACCGATGTATTTGCATTTGGTGTGGTTCTGGCAGAGTTGATAACAGGGAAACGTGCACTATTTCGTGACAACCAAGA-------AGC---CAGCAATATGAAATCACTTACTTCAGTTGTTGGCCAAATATTCAAAGATG---ATGACCCAGAGACTGTTTTAG------CAGATGCCATAGATGGGAATCTTCAACGTAGCTATCCTATGGAAGATGTCTACAAGATGGCAGAACTAGCTCATTGGTGCTTGTGCGAAGATCCAAATGTCAGGCCTGAGATGAGGGAGATTGTTGTGGCATTGTCACAGATTGTGATGTCCTCCACAGAATGGGAAGCATCACTAGGTGGAGACAGAGAGGTCTTCAGCGGG---GTACTTGATGGAAGATGA----

>Mucpru_QJKJ01008103.1_CDS

---------------------------------------------------------------------------------------------------ATGGCTT---ATCTAACTTTACTTCTATCTCTTCTCCT---TTTTCTGGTAAC-------TTCCTG--------TATAACTGTGTTTTCCTTTC---ATGTTTCAATCAAGT---CAACATACTTGGAACCTTTCAAATGCTCTGCTAAG---AT-CACCACATGC--AATGCCTCACTCTACCACATAAGCTACA---GTCACAA------CATACATGACATAGCCA---------------ACTTTTACTCTGTCGATCCC---TCCCAAATCAAACCTATAACGCGT---------GGCACCAATCA---AGATTACCTTGTAACAGTACCTTGTTCTTGCAGAAGCATCAATGA------CCTTGTCGGGTATTTCTATGATACATCCTACAAG---GTGAACTCCACTGACACGTTTGTGGATATTAACAACTTTATTTACAGTGGCCAAGCCTGGCCTGTTAACA------GAAATTTGGT------CCCAGATGAGGACTTAACAATACATCTTCCTTGTGGGTGTTCACAAAA------------AAGTGACTCCCAAATTGTTG-TCACGTACACAGTTC-AGCGGAATGA-TACACCCACCTCAATTGCTTCTATGCTAAATGCTACGTTAGATGGCATGGTGGGTATGAACCAAGTTCTGGCTCAGAACCCCTCTTTCATAGATGTTACTTGGGTGTTGTATGTTCCCAT---GGAATTGAATGGGTTATCACT------------------TTCCAACGGAAAAGAAAAGAAACAAAA---GTTG---GAGATAATCGTTGGAATTTTAGTGGGTG---TGACATTACTTTCAATTATTACCTTGATCATTCTCATTGTCGTTCTTAGGAGATCAAGA---GCAAATAAAAC---CGCCAAAAATGATCCA---AGTGCTGTCTCTAAAAGATCAATTGCCAATAG---------------------------AAC---TATTTCCTTGAAGAACCGA-GACT-TTCATACAGAATATACTGAAGATGCAACACCATTTGAGTCAGAAAGACCAGTAGTTTATACTCTAGAGAGGATTGAAGATGCTACCAATAACTTTGATGAAACCCGAAAGATTGGGGTAGGTGGATACGGTAGTGTGTATTTCGGAATGTTAGAAGAAAAGGAGGTTGCTGTGAAGAAGATGAGGTCTAATAAATCCAAAGAATTCTATGCAGAACTCAAGGCCTTGTGTAAGATCCATCATATTAACATTGTGGAGTTGTTGGGATATGCTAGTGGAGATGACCACCTTTATTTGGTGTATGAGTATGTTTCAAATGGATCTCTCAGTGAACATCTTCATGATCCGTTACTGAAAGGACACCAGCCTCTTTCTTGGTGTGCTAGGATTCAAATTGCACTGGATTCAGCAAAAGGTCTTGAATACATACATGATTACACAAAAGCACGATATGTGCATCGTGATATAAAGACTAGTAATATTCTTCTTGATGAGAAGCTCAGGGCAAAGGTGGCAGATTTCGGACTTGCAAAGCTAGTAGAACGAACCAATGATGAAGAATT---CATAGCAACAAGGCTTGTAGGAACACCAGGCTACCTTCCGCCAGAATCTCTGAAGGAGCTTCAAGTGACTATAAAAACAGATGTATTTGCATTTGGCGTGGTTCTGTCAGAGTTGATAACAGGGAAACGTGCACTATTTCGTGACAACCAAGA-------AGC---CAAAAATATGAAATCACTCGTTACAGTTGTTGACCAAATATTCAAAGATG---AATACCCAGAGACTGTTTTAG------CAGATGTCATAGATGGGAATCTTCAACATAGCTATCCTATGGAAGATGTCTACAAGATGGCAGAACTAGCTCATTGGTGCTTGCGTGAAGATCCAAACGAGAGGCCTGAGATGAGGGAGATCGCTGTGGCACTGTCACAGATTGTGATGTCCTCCATAGAGTGGGAAGCATCACTAGGTGGAGACAGCCAGGTCTTCAGCGGG---GTACTAGATGGAAGATGA----

>CM014730.1_CDS

---------------------------------------------------------------------------------------------------ATGGCTT---CTCTAACTCCACTTCTTTCTCTTGTCCTCACTCTTCTGACAAG-------TTCATG--------TGTAACTGTGTTTTCCTTTG---AAGTTTCAATCAAAA---CAACTTACTTGAAACCTTTCAAATGCTCCCCTAAG---AT-CAGCACATGC--AATGCCTCACTCTACCATATAAGCTACG---GTCTCAA------CATAGATGACATAGCCA---------------ACTTTTACTCTGTCAATACC---TCCCAAGTCAAACCTATAATGCGT---------GGCGCCAATCA---AGATTACCTTGTAACAGTACCTTGTTCTTGCAATAATTCCATTGA------CCT------ATATTTCTATGATACAACTTACAAG---GTGAAGTTTAATGACACGTTTTTGGATATTAGCAACTTTATTTACAGTGGCCAAGCCTGGCCTATTAACG------GAGAATTGGA------CCCAGCTGAGAAGTTAACAATACATCTTCCTTGTGGGTGTTCAGAAAA------------AAGTGACTCCCAAATTGTTG-TCACGTATACAGTTC-AGCGGAATGA-TACACCCACATCAATTGCTACTATGCTAAATGCTACGTTTGATGGCATGGTGAGTATGAACGAAGTTCTGGCTCAGAACCCCTCATTCATAGATGTTAGTTGGGTGTTATATGTTCCCAG---GGAATTGAATGGATTACCACT------------------TTCCAAAGGAAAAGAAAAGAAACACAA---GTGG---GAGATAATCATTGGCATCTTAGCGGGTG---TGACATTACTTTCGATTATTACCTTGATCATTCTCATTGTCATTCTCAGGAGATCCAGA---GCCTATGAAAC---CGCCAAAAATGATCCA---CGTGCTCTCTCTAAGAGATCAATTGCCAATAG---------------------------AAC---TATTTCTGTAATGAACCGT-GACT-TTCATACAGAATATATTGAAGATGCAACACCATTTGAGTCAGAACGACCAGTAATTTATACTCTAGAGGATATTGAAGATGCTACGAATAACTTCGATGAAACCCGAAGGATTGGAGTAGGTGGATACGGTAGTGTGTATGTCGGAATGTTAGAAGAGAAGGAGGTTGCTGTGAAGAAGATGAGGTCTAATAAATCCAAAGAATTCTATGCAGAACTCAAGGCCTTGTGTAAGATCCATCATATTAACATTGTGGAGTTGTTGGGATATGCAAGTGGAGATGACCACCTTTATTTGGTGTATGAGTATGTTTCAAATGGATCTCTCAGTGAACATCTTCATGATCCATTACTGAAAGGTCACCAGCCTCTTTCATGGTGTGCTAGGTCTCAAATTGCACTGGATGCGGCAAAAGGTCTTGAATACATACATGATTATACAAAAGCACGATATGTGCACCGTGATATAAAAACTAGTAATATTCTTCTTGATGAGAAGCTCAGAGCAAAGGTAGCAGATTTCGGACTTGCAAAGTTAATAGAACGAACCAATGATGAAGAATT---CATAGCAACCAGGCTTGTTGGAACACCAGGCTACCTTCCACCAGAATCTGTGAAGGAGCTTCAAGTGACTATAAAAACCGATGTATTTGCATTTGGTGTGGTTCTGTCAGAGTTGATAACAGGGAAACGAGCACTATTTCGTGACAACCAAGA-------AGC---CAACAATATGAAATCGCTTATTACAGTTGTTGACCAAATATTCAAAGATG---ATGACCCAGAGACTTTTTTAG------CTGATGCCATAGATGGGAATCTTCAACGTAGCTATCCTATGGAAGATGTCTACAAGATGGCAGAACTAGCTCATTGGTGCTTACGCGAAGATCCAAACGACAGGCCTGAGATGAGGGAGATCGTAGTGCCATTGTCACAGATTGTGATGTCCTCCATAGAATGGGAAGCATCACTAGGTGGAGACAGCCTGGTCTTCAGCGGG---GTATTTGATGGAAGATGA----

>LOC108328851_CDS

---------------------------------------------------------------------------------------------------ATGGCTT---CTCTAACTTCTCTTCTTTCTCTTCTCATTCCTCTTCTTGCAAC-------TTCTTGTGTAAG--TGTAGCTGTGTTTTCCTTCC---AAGCTTCTCTCAAAA---CAACTTACTTTGAACCGTTCAGATGCTCCTCCATG---AT-CAGCACATGC--AATGCCTCACTCTACCACATTAGTTACA---ATCACAA------CGCAGATGACTTAGCCA---------------ACTTTTACTCCGTTCACCCC---TCCCAAATCAAACCTATAATGCGT---------GGCACAAAGCA---AGATTACCTTATAACAGTGCCTTGTTCCTGCAATAGCTCCAATGA------CCTTGGCGGATATTTCTATGATACAACCTACAAG---GTGAAGCCTAATGACTCTTCTGTGGAGATTAACAACATTGCGTACAGTGGCCAAGCCTGGTCTATTAACA------GAGAAGTGAA------CCCAAACGAGGAATTAGCAATAAATCTTCCTTGTGGGTGTTCAGAAAA------------AAATGACTCTCAAATTGTTG-TCACGTATACAGTTC-AGCGGAGTGA-TACACCCATATCAATCGCTACTCTGCTAAATGCTACCTTAGAAAACATGGTGAGTATGAACGAAGTTCTGGTTCAGAACCCCTCATTCATAGATATTAGTTGGGTGCTGTATGTTCCAAG---GGAACTCAATGGTTTGCC---------------------TTCCAATGGAAAAGAAAAGAAACACTA---T---------ATAATCATTGGCATCTTGGCGGGTG---TGACATTCTTTTCAATTATTACTTTGATAATTCTCGTTGTCGTTCTTAGGAGATCCAGG---GCCAGTGTAGC---CGCAAAAAATGATCCA---AATATTGTGTCTAAAAGATCAATTGGAAATAG---------------------------AAC---TATTTCCATAAAGGAC-------T-TTCATGCAGAACATATAGAAGATGCAACTCCATTTGAATCAGAAAGACCAGTAATTTATGCTCTAGAGGAGATTGAAGATGCTACAAATAACTTTGATGAAACGCGAAAGATTGGAGTAGGTGGATACGGGAGTGTGTATTTCGGAATGTTAGAGGAGAAGGAGGTTGCAGTAAAGAAGATGAGGTCTAATAAATCCAAAGAATTCTATGCAGAACTCAAGGCCTTGTGTAAGATCCATCACATTAACATTGTGGAGTTGTTGGGATATGCCAGCGGAGATGACCACCTTTATTTGGTGTATGAGTATGTTCCAAATGGATCTCTCAGTGAGCATCTTCATGATCCATTACTGAAAGGTCACCAGCCTCTTTCTTGGTGTGCTAGGGTTCAAATTGCATTGGATGCAGCAAAAGGTCTGGAATACATACATGATTACACGAAAGCTCGATATGTGCACCGTGATATAAAGACTTGCAATATTCTCCTTGATGAGAAGCTAAGAGCAAAGGTAGCAGATTTTGGACTTGCAAAGCTAGTAGAACGAACCAATGATGAAGAATT---CATAGCAACAAGGCTTGTTGGAACACCGGGCTACCTTCCACCAGAATCTGTTAAGGAGCTTCAAGTGACTATAAAAACAGATGTTTTTGCATTTGGGGTGGTTCTGTCAGAGTTGATAACAGGGAAACGTGCACTATTTCGTGACAACCAACA-------AGC---CAACAATATGAAATCACTTGTTACAGTTGTTAGCCAAATTTTCAGAAATA---AATACCCAGAGAATGCTTTAG------CAGATGCCGTAGATGGGAATCTTCAGCATAGCTATCCCATGGAAGATGTCTACAAGATGGCAGAAGTAGCTCATTGGTGTTTGTGGGAAGATCCAAACGACAGGCCTGTCATGAGGGAGATAGTTGTGGCATTGTCAGAGATTGTTATGTCATCCACAGAATGGGAAGCATCACTAGGTGGAGACAGCCAAGTCTTCAGCGGG---GTTCTTGATGGAAGATGA----

>LOC106777404_CDS

---------------------------------------------------------------------------------------------------------------ATGGCTTCTCTAACTTCTCTTCTCATTCCTCTTCTTGCAAC-------TTCTTGTGTAAC--TGTAGCTGTGTTTTCCTTCC---AAGCTTCTCTCAAAA---CAACTCACTTTGAACCGTTCAGATGCTCCTCCATG---AT-CAGCACATGC--AATGCCTCACTCTACCACATAAGTTACA---ATCACAA------CGCAGATGACTTAGCCA---------------ACTTTTACTCCGTTCACCCC---TCCCAAATCAAACCTATAATGCGT---------GGCACAAAGCA---AGATTACCTTATAACAGTCCCTTGTTCCTGCAATAGCTCCAATGA------CCTTGGCGGATATTTCTATGATACAACCTACAAG---GTGAAGCCTAATGACTCTTCTGTGGAGATTAACAACATTGTTTACAGTGGTCAAGCCTGGTCTATTGACA------GAGAAGTGAA------CCCAAACGAGGACTTAGCAATACATCTTCCTTGTGGGTGTTCAGAAAA------------AAAAGACTCCCAAATTGTTG-TCACGTATACAGTTC-AGCGGAATGA-TACACCCATATCAATCGCTACTCTGCTAAATGCTACCTTAGATGACATGGTGAGTATGAACGAAGTTCTGGTTCAGAACCCCTCATTCATAGATATTAGTTGGGTGCTGTATGTTCCAAG---GGAACTCAATGGTTTGCC---------------------TTCCAAAGGAAAAGAAAAGAAACACTA---T---------ATAATCATTGGCATCTTAGCGGGTG---TGGCATTCTTTTCAACTATCACTTTGATCATTCTCGTTGTCGTTCTTAGGAGATCCAGG---GCCAGTGTAGT---CGCAAAAAATGATCCA---AATATTGTGTCTAAAAGATCCATTGGAAATAG---------------------------AAC---TATTTCCATAAAGGAC-------T-TTCATGCAGAACATATAGAAGATGCAACTCCATTTGAATCAGAAAGACCAGTAATTTATGCTCTAGAGGAGATTGAAGATGCTACAAATAACTTTGATGAAACGCGAAAGATTGGAGTAGGTGGATACGGGAGTGTGTATTTCGGAATGTTGGAGGAGAAGGAGGTTGCAGTAAAGAAGATGAGGTCTAATAAATCCAAAGAATTCTATGCAGAACTCAAGGCCCTGTGTAAGATCCATCACATTAACATTGTGGAGTTGTTGGGATATGCCAGCGGAGATGACCACCTTTATTTGGTGTATGAGTATGTTCCAAATGGATCTCTCAGTGAGCACCTTCATGATCCATTACTGAAAGGTCACCAGCCTCTTTCTTGGTGTGCTAGGGTTCAAATTGCATTGGATGCAGCAAAAGGTCTTGAATACATACATGATTACACGAAAGCTCGATATGTGCACCGTGATATAAAGACTTCCAATATTCTCCTTGATGAGAAGCTAAGAGCAAAGGTAGCAGATTTTGGACTTGCAAAGCTAGTAGAACGAACCAATGATGAAGAATT---CATAGCAACAAGGCTGGTTGGAACACCGGGCTACCTTCCACCAGAATCTGTTAAGGAGCTTCAAGTGACTATAAAAACAGATGTTTTTGCATTTGGGGTGGTTCTGTCAGAGTTGATAACAGGGAAACGTGCACTATTTCGTGACAACCAACA-------AGC---CAACAATATGAAATCACTTGTTACAGTTGTTAGCCAAATCTTCGGAAATA---AATACCCAGAGAATGTTTTAG------CAGATGCCATAGATGGGAATCTTCAGCATAGCTATCCCATGGAAGATGTCTACAAGATGGCAGAAGTAGCTCATTGGTGTTTGTGCGAAGATCCAAACGACAGGCCTGTGATGAGGGAGATAGTTGTGGCATTGTCACAGATTGTTATGTCATCCACAGAATGGGAAGCATCATTAGGTGGAGACAGCCAAGTCTTCAGCGGG---GTTCTTGATGGAAGATGA----

>Vigun02g080500_CDS

---------------------------------------------------------------------------------------------ATGATCCCACAAA---CCATGTCTTCTCTTCTTTCTCTTCTCATTCCTCTTCTTGCTAC-------TTCTTGTGTAAG--TGTAGCTGTGTTTTCCTTCC---AAGCTTCTCTCAAAA---CAACTTACTTGGAACCCTTCAGATGCTCCTCTATG---AT-CAGCACATGC--AATGCCTCACTCTACCACATAAGTTACA---ATCACAA------CGCAGATGACTTAGCCA---------------ACTTTTACTCCGTTCATCCC---TCCCAAATCAAACCTATAATGCGT---------GGCACAAAGAA---AGATTACCTTGTAACAGTGCCTTGTTCCTGCAATAACACTAATGATGATGACCTTGGCGGATATTTCTATGATACAACCTACAAG---GTGAAGCCTAATGACTCTTCTGTGGAGATTAACAACGTTGTTTACAGTGGCCAAGCCTGGTCTATTAACA------CAGAAGTGGA------CCCAAATGAGGACTTGGCAATACATCTTCCTTGTGGGTGTTCAAAAAA------------AAGTGACTCCCAAATTGTTG-TCACGTATACAGTTC-AGCGGAATGA-TACACCCATATCAATCGCTACACTGCTAAATGCTACCTTAGATGACATGGTGAGTATGAACGAAGTTCTGGTTCAGAACCCTTCATTCATAGATATTAATTGGGTGCTGTATGTTCCAAG---GGAACTCAATGGTTTGCC---------------------TTCCAAAGGAAAAGAGAAGAAACACTA---T---------ATAATCATTGGCATCTTAGCGGGTG---TGACATTCTTTTCAACTATTACTTTGATCATTCTCGTTGTCGTTCTTAGGAGATCCAGG---GCCAGTGTAGC---CGCAAAAAATGATCCA---AATATAGTGTCTAAAAGATCAATTGGAAATAG---------------------------AAC---TATTTCCATAAAGAACCGA-GATT-TTCATGCAGAACATATAGAAGATGCAACTCCATTTGAATCAGAAAGACCAGTAATTTATGCTCTAGAGGAGATTGAAGATGCTACGAATAACTTCGACGAAACACGAAAGATTGGAGTAGGTGGATACGGGAGTGTGTATTTCGGAATGTTAGAGGAGAAGGAGGTTGCTGTAAAGAAGATGAGGTCTAATAAATCCAAAGAATTCTATGCAGAACTCAAGGCCTTGTGTAAGATCCATCACATTAACATTGTGGAGTTGTTGGGATATGCCAGCGGAGATGACCACCTTTATTTGGTGTACGAGTATGTTCCAAATGGATCTCTCAGTAAGCACCTTCATGATCCATTACTGAAAGGTCACCAGCCTCTTTCTTGGTGTGCTAGGGTTCAAATTGCATTGGATGCAGCAAAAGGTCTTGAATATATACATGATTACACGAAAGCTCGATATGTGCATCGTGATATAAAGACTTGCAATATTCTCCTTGATGAGAAGCTTAGAGCAAAGGTAGCAGATTTTGGACTTGCAAAGCTGGTAGAACGAACCAATGATGAAGAATT---CATAGCCACAAGGCTTGTTGGAACACCGGGCTACCTTCCACCAGAATCTGTTAAGGAGCTTCAAGTGACTATAAAAACAGATGTATTTGCATTTGGGGTGGTTCTGTCAGAGTTGATAACAGGAAAACGTGCACTATTTCGTGACAACCAACA-------ACC---CAACAATATGAAATCACTTGTTACAGTTGTTAGCCAAATCTTCAAAAACA---AATACCCAGAGAATGTTTTAG------CAGATGCCATAGATGGGAATCTTCAGCATAGCTATCCAATGGAAGATGTCTACAGGATGGCAGAAGTAGCTCATTGGTGTTTGTGCGAAGATCCAAACGACAGGCCTGTGATGAGGGAGATAGTTGTGGCATTGTCTCAGATTGTTATGTCATCCACAGAATGGGAAGCATCATTAGGTGGAGACAGCCAAGTCTTCAGTGGG---GTTCTTGATGGAAGATGA----

>PhvuI.002G059500_CDS

------------------------------------------------------------------------------------ATGATCACACAAACCATGGCTT---CTCTAACTTCACTTCTTTCTCTTCTCATTCCTCTTCTTGCAAC-------TTCTTATGTGGG--TGTAGCTGTGTTTTCCTTAC---AAGCTTCTCTTCAAA---CAACTTATTTGGAACCTCTCAGATGCTCCTCTAAG---AT-CAGCACATGC--AATGCCTCACTCTACCACATAAGTTACA---GTCACAA------TGCAGATGAGATAGCCA---------------ACTTTTATTCCGTTCATCCC---TCCCAAATCAAACCTATAACGCGT---------GGCACAAAGCA---AGATTACCTTATAACAGTGCCTTGTTCCTGCCATAACACCAATGG------CCTTGCCGGATATTTCTATGATACAACCTACAAG---GTGAAGCCTAATGACTCGTCTGTGGAGATTAACAACATTGTTTACAGTGGCCAAGCCTGGTCTATTAATA------GAGAAGTGGA------CCCAAATGAGGACTTGGCAATACATCTTCCTTGTGGGTGTTCAGAAAA------------AAATGACTCCCAAATTGTTG-TCACCTATACAGTTC-AGCGGAATGA-TACACCCACATCAATCGCTACTATGCTAAATGCTACGCTAGATGGCATGGTTAGTATGAACGAAGTTCTGGCTCAAAACCCCTCATACATAGATATTACTTGGGTGCTATATGTTCCAAG---CCAACTCAATGGGTTGCC---------------------TTCCAAAGGAAAAGACAAGAAACACCA---T---------ATAATCATTGGCATCTTAGCGGCTG---TGGCATTCTTTTCAACTATTACTTTGATCATTCTCGTTGTCGTTCTTAGGAGATCCAGG---GCCAATGTAAC---CGCCAAAAATGATCCA---AATATTGTGTCTAAAAGATCAGTTGGATTTAG---------------------------AAC---TATTTCCATAAAGAACCGA-GACT-TTCATGCAGAACATGGAGAAGATGCAACTCCATTTGAATCAGAAAGACCAGTAATTTATGCTCTAGAGGAGATTGAAGAGGCTACGAATAACTTTGATGAAACGCGAAGGATTGGAGTAGGTGGATACGGGAGTGTGTATTTCGGAATGTTAGAG---AAGGAGGTTGCTGTAAAGAAGATGAGATCTAATAAATCCAAAGAATTCTATGCAGAACTCAAAGCCTTGTGTAAGATCCATCACATTAACATTGTGGAGTTGTTGGGATATGCTAGCGGAGATGACCACCTTTATCTGGTGTATGAGTATGTTCCAAATGGATCTCTCAGTGAACACCTTCATGATCCATTGCTGAAAGGTCATCAGCCTCTCTCTTGGTGTGCTAGGATTCAAATTGCATTGGATGCAGCAAAAGGTCTTGAATACATACATGATTACACGAAAGCACGATATGTGCATCGTGATATAAAGACAAGTAATATTCTCCTTGACGAGAAGCTCAGAGCAAAGGTAGCAGATTTTGGACTTGCAAAGCTAGTAGAACGAACCAATGATGAAGAATT---CATAGCAACAAGGCTTGTTGGAACACCGGGCTACCTTCCACCAGAATCTGTTAAGGAGCTTCAAGTGACTATAAAAACAGATGTATTTGCATTTGGGGTGGTTCTGTCAGAGTTGATAACAGGGAAACGTGCACTATATCGTGACAGCCAACA-------AGC---CAACAATATGAAATCACTTGTTACGGTTGTTAGCCAAATCTTCGGAAATA---AGTACCCACAGACTGTTTTAC------CAGATGTCATAGATGGGAATCTTCAGCATAGCTATCCCATGGAAGATGTCTACAGGATGGCAGAACTAGCTCATTGGTGTTTGTGCGAAGATCCAAATGACAGGCCTGAGATGAGGGAGATGGTTGTGGCATTGTCACAGATTGTTATGTCATCCACCGAATGGGAAGCATCACTAGGTGGAGACAGCCAAGTCTTCAGCGGG---GTACTTGATGGAAGATGA----

>LOC113869898

---------------------------------------------------------------------------------------------------ATGGCTT---CTCTTACTCAACTTCTTTCTCTTTTCCTCCCTCTTCTAGTAAC-------TTCATGTGTAC-----CTGCTGAATCTTCCCTTG---AAGTTTCTATCAAAT---CAACCTACTTGGAACCTTTTAATTGCTCTACAAAG---AT-CAGGACATGC--AATGCCTCACTCTACCACATAAGCTATG---ATCTCAA------CATAAATCAAATAGCCA---------------ACTTTTACTCTGTTAATCCA---TCCCATATCAAACCCATAATGCGT---------GGGACCAAACA---GGATTACCTTATAACTATACCTTGTTCTTGCAAAAACACTATTGA------CCTTAGCGGATATTTCTATGATACAACCTACAGA---GTGTTGCCTGATGACACTTTTGTGGACATCAACAACCTTGTTTTCAGTGGCCAAGCTTGGCCTATTGATG------GAGAATTGGT------CCCAAATGAGAATTTAACAATACATCTTCCATGTGGGTGCTCAGAGAA------------AAGTGACTCTCAAATTGTTG-TCACCTATACAGTGC-AGCGGAATGA-TACTCCCACATCGATTGCTATTCTGCTAAATGCTACGGTTGATGGCATGGTGAGTAGGAACGAAGCTTTGGTTCAGAACCCCACATTCATTGATGTTACTTGGGTGCTATATGTTCCTAT---GGAATTGAATGGGTTACCACT------------------TTCCAAAGGAAAAGGAATGAAACTCAA---GTGG---GTGATAATCATTGGCATCTTAGGGGGTG---TGGCAGTATTTTCAATTATTACCTTAATCATTCTCATCGTCGTTGTCAGGATAACCAGA---GCCCATGAAAC---CAGAAATAATGATCCG---AATTCTCTCTCTAAAAGGACAATTTCCAATAG---------------------------AAC---TGTTTCCTTAAAGAACCGA-AACT-TTCATACAGAATTTATGGAAGATGCAACACAATTTGAGTCAGAAAGACCAGTAATTTATACTCTTGAGGAGATAGAAGATGCCACTAACAACTTCAATGAAAACCGAAGGATTGGAGTTGGTGGATATGGGAGTGTGTATTTTGGAATGTTAGGGGAGAAGGAGGTTGCTGTGAAGAAGATGAGGTCTAATAAATCCAAGGAATTCTATGCAGAACTCAAGGCCTTATGTAAGATCCATCATATTAACATTGTGGAGTTGTTAGGATATGCTAGCGGAGATGACCACCTTTATTTGGTGTATGAGTATGTTCCAAATGGATCTCTCAATGAGCATCTTCATGATCCATTACTGAAAGGTCACCAGCCTCTTTCTTGGTGTGCTAGGATTCAAATTGCACTGGATGCAGCAAAAGGCCTTGAATACATACATGATTACACAAAAGCACGATACGTGCACCGTGATATAAAGACTAGTAATATTCTTCTTGATGAGAAGCTCAGAGCAAAGGTAGCAGATTTTGGACTTGCAAAGCTTGGAGAACGAACCAATGATGAAGAATT---TATAGCGACAAGGCTTGTTGGAACACCAGGCTACCTTCCACCAGAATCTGTGAAGGAGCTTCAAGTGACCGTGAAAACGGATGTATTTGCATTTGGTGTAGTTCTATCAGAGTTGATAACAGGCAAACGTGCGCTATTTCGTGA----------------AGC---CAACAACATGAAATCTCTTATTACCGTCATTGGCCAAATATTCCAAGCGG---ATGAGCCAGAGACTGCTTTAG------CAGATTCCATTGATAGGAATCTTCAACATAGCTATCCTATGGAAGATGTCTACAAGATGGCAGAACTAGCTCATTGGTGCTTGTGCGACGATCCAACGGACAGGCCTGAAATGAGGGAGATGGTTGTGGCATTGTCACAGATTGTAATGTCCTCCGTAGAGTGGGAAGCATCACTGGGTGGAGACAGC---GTCTTCAGCGGG---GTATTTGATGGAAGATGA----

>Pissat_chr2LG1_CDS

---------------------------------------------------------------------------------------ATGATCAAAACTATGGCTTATTCTCTAATTCAACTTCTTTCACTTCTTCTCCCTCTTCTATCATC-------TTCATTTATAA-----CAACTGTGTTCACCTTTG---AAGTTTCAATAAAAA---ACACTTACATAGAACCTTTTAAATGCTCTACAAAG---AT-CAAAACATGC--AATGCATCAATCTACCACATAAACTACA---ATCACAG------CATAGAACAACTAGCCG---------------ATTCTTACTCCGTCGATCCT---TCCGAAATCAAACCGATATTTCGT---------AGCACAAAGCA---AGATTACCTTATACAAGTACCTTGTTCTTGCCAAGACACCATTGA------CCTTAACGGTTATTTCTATGACACAAACTACAAA---GTGAGTCATAATGAAAGTTTTTTGAATATTAACAATATTGTTTATAGTGGTCAAACTTGGCATGTCAATG------AAGATTTGGT------TGCAAATGAGAATGTGACGATACATATTCCTTGTGGATGTTCAGA---------------ACTTGAGTCACAAATTGTTG-TTACATATACTGTTC-AGCAAGGTGA-TACACCAACATCAATTTCTCTTTTGTTAAATGCTAATCTTGATGGCATGGTGAAAATGAATGAAATTTTGGGTCCTAACCCAACATTCATTGATATTGGTTGGGTTTTATATGTTCCCAA---GGAATTAAAAGGGTCACCACT------------------TTCCATTGAAAAAGAAAAGAAACACAA---GTGG---GTGATAATTATTGGTATCTTAGTGAGTG---TGACATTACTTTCGGTTATTAGCTTGATGATTTTCA------TTCTCAGAAGAAATAAA---GGCTATGGAAG---TAGCAAAAATGATCCA---AAAACTTTCTCTAAAAGATCGCTTGCCAATAG---------------------------AAC---CATTTCTCTAAGGAATCAA-GAAT-TTCATAAAGAATATATGGAAGATGCAACTCAATTCGATTCCGAAAGACCAGTGATCTATGATTTTGAGGAGATTGAAGAAGCTACAAATAACTTTGATGAAACTAGAAGGATTGGAGTTGGTGGATATGGCACAGTCTATTTTGGAATGCTAGAAGATAAAGAAGTTGCTGTGAAGAAAATGAAGTCTAACAAATCCAAAGAGTTCTATGCAGAACTCAAAGCCTTATGCAAGATCCATCATATTAACATTGTTGAATTGTTGGGATATGCAAGTGGAGATGATCATCTTTATTTGGTGTATGAGTATGTTCCTAATGGATCTCTAAGTGAACACCTTCATGATCCATTATTAAAAGGTCACCAGCCTCTTTCTTGGTGTGCTAGAGCTCAAATTGCACTTGATTCAGCAAAAGGTATTGAATACATACACGATTACACAAAAGCGCGATATGTGCACCGTGATATAAAAACTTGTAATATCCTTCTTGATGCAAAGCTCAGAGCAAAGGTAGCGGATTTCGGGCTTGCAAAGCTCGTGGAACGAACAAACGATGAAGAATT---TTTAGCAACAAGACTTGTTGGAACACCAGGCTATCTTCCACCTGAATCGGTGAAGGAGCTTCAAGTGACGATAAAAACTGATGTATTTGCATTTGGCGTGGTTCTGTCTGAGTTGATAACTGGGAAACGCGCATTGTTTCGTGACAACAAAGA-------AGC---TAATAATATGAAATCACTTATTGCAGTTGTAAACAAAATATTTCAAGATG---ATGAGCCTGTGATTGCTTTAG------AAGAAGCTGTAGATGGAAATCTTCTACGTAGTTATCCTATGGAAGATGTCTACAAGATGGGTGAATTATCACATTGGTGTTTGAGTGAAAATCCAGTGGACAGACCTGAAATGAAAGAGATAGTTGTTGTATTGTCAAAGATTGTAATGTCTTCAATAGAATGGGAAGCGTCACTAGGTGGAGACAGTCAAGTTTTCAGTGGT---GTATTTGATGGAAGATGA----

>VsEPR3_CDS

---------------------------------------------------------------------------------------------------ATGGCTTGTTCTCCAATTCAACTTCTTTCACTTCTTCTCCCTCTTCTATTATC-------TTCATACATAA-----CAGGTGTTTTCACCTTTG---AAGTTTCAATAAAAA---ACACGTACATAGAACCTTTTAAATGTTCTACAAAG---AT-CAGAACATGC--AATGCATCACTCTACCACATAAACTACA---ATCACAA------CATAGAACAACTAGCCG---------------ATTTTTACTCCGTTGATCCT---TCCCAAATCAAACCGATAATCCGT---------AGCACAAAACA---AGATTACCTTATAAATGTGCCTTGTTCTTGCCAAGACACCATTGA------CCTTTACGGTTATTTCTATGACACAAACTACAAA---GTGAGTCCGAATGAGAGTTCTATGAATATTAACAACCTTGTTTATAGTGGTCAAACTTGGCATGTCAATG------AAGATTTGGT------TGCAAATGAGAATGTAACAATACATATTCCTTGTGGATGTTCAGA---------------ACTTGAGTCACAAATTGTTG-TTACATATACCGTTC-AGCAAGGTGA-TACACCAACATCAATTTCTCTTTTGTTAAATGCTAATATTGATGGCATGGTGAAAATGAATGAAATTTTGGGTCCTAACCCAACATTCATTGATATTGGTTGGGTTTTATATGTTCCTAA---GGAATTAAAAGGGTCACCAAT------------------TTCCAATGGAAAAGAAAAGAAACACAA---ATGG---GTGATAATTATTGGTATCTTAGTGAGTG---TGACATTACTTTCAATTATTACCTTGATGGTTTTCA------TTCTCAGAAGAAATAAA---GGCTATGGAAG---TAGCAAAAGTGATCCA---AAAACTTTTTCTAAAAGATCACTTGCCAATAG---------------------------AAC---TATTTCTCTAAGGAATCAA-GAAT-TTCATAAAGAGTATATGGAAGATGCAACACAATTTGATTCAGAAAGGCCAGTGATTTATGATTTTGAGGAGATTGAAGATGCTACAAATAATTTTGATGAAACTAGAAGGATTGGAGTTGGTGGATATGGCACTGTCTATTTTGGAATGCTAGAAGATAAAGAAGTTGCTGTGAAGAAAATGAAGTCTACCAAATCCAAAGAGTTCTATGCAGAACTCAAAGCCTTATGCAAGATTCATCATATTAACATTGTGGAGTTGTTGGGATATGCAAGTGGAGATGATCACCTTTATTTGGTGTATGAGTATGTTCCTAATGGATCTCTAAGTGAACACCTTCATGATCCATTATTAAAAGGTCACCAGCCTCTTTCTTGGTGTGCAAGAGTTCAAATTGCACTTGATTCAGCAAAAGGTATTGAATACATACACGATTACACCAAGGCGCGATATGTGCACCGTGATATAAAGACTTGTAATATTCTTCTCGATGCCAAGCTCAGGGCAAAGGTAGCCGATTTCGGACTTGCAAAGCTTGTGGAACGAACCAACGATGAAGAATT---TTTAGCAACAAGACTTGTTGGAACACCAGGCTACCTTCCACCTGAATCGGTGAAGGAGCTTCAAGTGACGATAAAAACCGATGTATTTGCATTTGGCGTGGTTCTGAGTGAGTTGATAACCGGAAAACGCGCATTGTTTCGTGACAACCAAGA-------AGC---TAATAATATGAAATCACTTATTGCAGTTGTAAAGAAAATATTTCAAGATG---ATGAGCCTGTGCGTGCTTTAG------AAGAAGCTGTAGATGGAAATCTTCTACGTAGTTATCCTATTGAAGATGTGTACAAGATGGGAGAATTATCACATTGGTGTTTGAGTGAGAATCCAGTGGACAGACCAGAAATGAAAGAGATTGTTGTGGTGTTGGCAAAGATTGTAATGTCTTCAATAGAATGGGAAGCGTCACTAGGTGGGGATAGTCAAGTTTTCAGTGGT---GTATTTGATGGAAGATGA----

>MtLYK10_CDS

---------------------------------------------------------------------------------------------------ATGGCT---TCTCTAATTCAACTTCTTTCAATTTTTCTACCTCTTCTAGCATC-------TTCAT---TAC-----CAACTATATTTTCTATTG---AAGTTTCAATGAAAA---AAGCTTACATGGAGCCTTATAAATGTTCTACAAAG---AT-GAGAACATGC--AATGCATCACTCTACCATATAAACTACA---ATCACAA------CATAGAACAAATAGCTA---------------ATTTTTATTCTATTGATCCT---TCACAAATCAAACCAATAATTCGT---------AGCACAAAACA---AGATTACCTAGTAAAAGTGCCTTGTTCTTGCAAAAACATCAAAGA------TCTTAGTGGATATTTTTATGAAACAACCTACAAA---GTGAGTCCTAATGAAACTTCTGTGGATATTATGAATCTTATTTATAGTGGTCAAGCTTGGCAAGTTAACG------AAGATTTGGT------TGCAAATGAGAATGTAACAATACATATTCCTTGTGGATGTTCAGA---------------ATTTGAGTCACAAATTGTTG-TTACATATACTGTTC-AGCAAAGTGA-TACACCAACATCAATTTCTCTTTTGCTAAATGCTACTATTGATGGCATGGTGAGAATTAACCAAATTTTGGGTCCTAACCCAACATTCATAGATATTGGTTGGGTGTTATATGTTCCTAA---GGAATTAAAAGGGTCACCACT------------------TTACCATGGAAAAGAAAAGAAACACAA---GTGG---GTGATAATTATTGGTATCTTAGTGAGTG---TGACATTGCTTTCCGTAATTACCTTGATCATTTTCA------TTCTAAGGAGAAATAAA---GCCTATGAAAC---CAGCAAATATGATCCA---AAAACTGTCTCTAAAAGATCATTTGGCAATAG---------------------------AAC---TATTTCCTTAAGGAATCAT-GAGT-TTCATAAAGAATATATGGAAGATGCAACACAATTTGATTCAGAAAGACCAGTAATTTATGATTTTGAGGAGATTGAACATGCTACAAATAACTTTGATGAAACTAGAAGGATTGGAGTTGGTGGATATGGTACTGTCTATTTTGGAATGCTGGAGGAGAAGGAGGTTGCTGTGAAGAAAATGAAGTCTAACAAATCCAAAGAGTTCTATGCAGAACTCAAAGCCTTATGCAAAATCCATCATATCAATATTGTGGAGTTGTTAGGATATGCTAGTGGAGATGATCACCTTTATTTGGTGTATGAGTATGTTCCTAATGGATCTCTAAGTGAGCACCTTCATGATCCTCTATTGAAAGGTCACCAGCCTCTTTCTTGGTGTGCAAGAACTCAAATTGCACTTGATTCAGCAAAAGGTATTGAATACATACATGATTACACGAAAGCGCGATATGTGCACCGTGATATAAAGACTAGTAATATTCTTCTTGATGAGAAGCTACGAGCCAAGGTAGCCGATTTCGGACTTGCAAAGCTAGTCGAAAGAACCAATGATGAAGAATT---TTTAGCAACAAGACTTGTTGGAACACCAGGCTACCTTCCACCAGAATCTGTGAAGGAGCTACAAGTGACAATAAAAACTGATGTATTTGCATTTGGAGTGGTTATATCTGAGTTGATAACAGGGAAACGTGCATTATTTCGTGACAACAAAGA-------AGC---TAATAATATGAAATCACTTATTGCAGTTGTTAACAAAATATTTCAAGATG---AGGACCCAGTAGCTGCTTTGG------AAGCTGTTGTAGATGGAAATCTTCTACGTAATTATCCTATAGAAGGTGTCTACAAGATGGCAGAATTATCACATTGGTGTTTGAGTGAAGAACCAGTGGACAGGCCTGAAATGAAAGAGATCGTTGTGGCAGTGTCAAAGATTGTAATGTCCTCAATAGAATGGGAAGCATCACTAGGTGGGGACAGTCAGGTTTTCAGCGGT---GTATTTGATGGAAGATAAA---

>Tp57577_TGAC_v2_LG4_CDS

---------------------------------------------------------------------------------------------------ATGGCTTATCTAATTCATCACCATCTTTCACTTTTTCTTCTTTTTCTAGCATT-------TTCAT---TAA-----CAAGTGTTTTTTCCATTG---AATTTTCAATCAAAA---AATCTTACATGGAACCTTTTAAATGTTCCACAAAG---AT-CAAAACATGC--AATGCATCACTCTATCACATAAACTACG---ATCACAA------CATAGAACAAATAGCAA---------------ATTTTTATTCTGTTGATTCT---TCCCAAATCAAACCGATTATTCGT---------AGCACGAAACA---AGATTACCTTATAAAAGTACCTTGTTCTTGCAAAAAAACCAATGA------GCTTAGTGGTTATTTCTATGATACAATCTATACC---GCGAGGCGTAATGAAAGTTTTGTCGATGTTAAGAATCTTGTTTATAGCGGTCAAACTTGGCAAGACGGCG------AAGATTTAGC------TGCAAATGAGAATGTAACAATACATATTCCTTGTGGATGTTCTGA---------------ATTTGAGTCAGAAATTGTTG-TTACATATACTGTTC-AGCAGAATGA-TACACCAACATCAATTTCTCTTTTGCTTAATGCTACTGTTGATGGCATTGTTAGAATGAACCAAATTTTGGTTCCTAACCCTTCATTTATAGATATTGGTTGGGTGTTATATGTTCCTGATGAGGAATTAAAACGGTCCTCACA------------------TTCCAATCGAAAAGAAAAGAAACACAA---GTGG---GTGATAATTATTGGTATCCTATTGAGTG---TGACATTACTTTCAGTTATTACTGTGATTGTTCTCA------TTCTTAGGAGAAATAAA---GCTTATCCAAC---CAGCAAAACTGATCCA---AGAACTTTCTCTAAAAGATCATTAGCCAATAG---------------------------AAC---TATTTCCTTAAGAAATCAC-GAAT-TTCATAAAGAATATATGGAAGATGCAACACAATTTGAGTCAGAAAGACCAGTGATTTATGATTTTGAGGAGATTGAAGTTGCTACAAATAATTTTGATGAAACTAGAAGAATTGGAGTTGGTGGATATGGTACTGTCTATTTTGGAATGTTAGAAGATAAAGAGGTTGCTGTGAAGAAAATGAAATCTACCAAATCTAAAGAGTTCTATGCAGAACTCAAAGCCTTATGCAAGATCCATCACATTAACATTGTGGAGTTGTTAGGATATGCAAGTGGAGATGATCACCTTTATTTGGTTTATGAGTATGTTCCTAATGGATCTCTAAGTGAACACCTTCATGATCCACTATTGAAAGGTCACCAACCTCTTTCTTGGTGTGCTAGAGCTCAAATTGCACTAGATTCAGCAAAAGGTATCGAATACATACATGATTACACGAAATCACGATACGTGCACCGTGATATAAAGACTAGCAATATTCTTCTTGATGAGAAGTTGAGAGCAAAGGTAGCCGATTTTGGACTTGCAAAATTAGTAGAAAGAACTAATGATGAAGAATT---CTTAGCTACAAGACTTGTCGGAACACCGGGCTACCTTCCACCAGAGTCTGTGAAGGAGCTTCAAGTGACAATAAAAACTGATGTATTTGCATTTGGAGTGGTTCTGTCTGAGCTGATAACAGGGAAACGTGCTTTATTTCGTGACAACAAAGA-------AGC---CAATAATATGAAATCACTTATTGCAATTGTTAACAAAATATTTGAAGATG---TTGATCCAGTGACTGCTTTAG------AAGCTGTTGTAGATGGAAATCTTCAACGTAATTATCCTATTGAAGATGTCTACAAGATGGCAGAAGTATCGCATTGGTGCATGAGTGAAAATCCAGTGGACAGGCCAGAAATGAAAGAAACAGTTATAGTATTGTCAAAGATTGTAATGTCTTCAATAGAATGGGAAGCTTCACTTGGTGGGGACAGCCAAGTTTTCAGCGGT---GTATTTGATGGAAGATGA----

>Cicari_Ca2_CDS

---------------------------------------------------------------------------------------------------ATGTCT---TATCTAACTCAAATTCTTTCACTTTTTCTCATTCTTCTAGCATC-------TTCAC---TAC-----TAAGTGTGTTTTCAATTG---AAGTTTCAATAAAAA---ACACATACATAGAACCTTTTAAATGTTCTACAAAA---AT-AAGAACATGC--AATGCTTCACTCTACCACATAAACTACG---ATCAAAA------GATAGAACAAATAGCCC---------------ATTTTTACTCTATTGGTGTT---TCACAAATCAAACCCATAATTCGT---------AGCAATAAACA---AGATTACCTAGTAAAAGTGCCTTGTTCTTGCAAAAACACCAATGA------CCTTAGTGGATATTTCTATGAAACAAACTACATG---GTTAGTCCTAATGAGAGTTTTGTGGATGTTAACACACTTGTTTATAGTGGTCAAGCTTGGCAAGTCAACG------AAGATTTGGT------TCCAAATGAGAATGTAACAATAAATATTCCTTGTGGTTGTTCGGA---------------ATTTGAGTCACAAATTGTTG-TTACATATACAGTTC-AACATAGTGA-TACACCAAATTCAATTGCTCTTATGCTAAATGCTACTGTTGATGGCATGGTTAGAATGAATCAAGTTTTGGGTCCTAACCCTACATTCATAGATATTGGTTGGGTGTTATATGTTCCTAA---GGAATTAAAAGGATCTCCACT------------------TTCCCATGGAAAAGGAAAGAAACACAA---GTGG---GAAGCTATTATTAGTATCTTAGTGAGTG---TGACATTACTTTCAGTTATTACCATGATTGTTCTCA------TTCTCAGGAGACATAAA---CCCTATGGAAC---CACCAAAAAGGATCCA---AAAACTGTCTCTAAAAGATCATTTGGCAATAG---------------------------AAC---TATTTCCATAAGGAATCAT-GACT-TTCATAAAGAATATATGGAAGATGCAACACAATTTGATCCAGAAAGACCAGTAATTTATGCTTTTGAGGAGATTGAAGATGCTACAAATAACTTTGATGAAACTAGAAGGATTGGAGTTGGTGGATATGGAACTGTCTATTTTGGAATGCTAGATGATAAAGAGGTTGCTGTTAAGAAGATGAGATCTAACAAATCCAAAGAGTTCTATGCAGAACTCAAAGCATTATGCAAGATCCATCACATTAACATTGTTGAGTTATTGGGATATGCAAGTGGAGATGACCACCTTTATTTGGTGTATGAGTATGTTCCTAATGGATCTCTTAGTGAACACCTTCATGATCCACTACTCAAAGGTCACCAGCCTCTTTCTTGGTGTGCTAGAACTCAAATTGCACTTGATGCAGCAAAAGGTGTTGAATACATACATGATTACACAAAAGCGCGATATGTTCACCGTGATATAAAGACTAGTAATATTCTTCTTGATGCGAAGCTCAGAGCAAAGGTAGCAGATTTTGGACTTGCAAAACTAGTAGAAAGAACCAATGATGAAGAATT---CTTAGCAACAAGGCTTGTTGGAACACCAGGCTACCTTCCACCAGAATCTGTGAAGGAGCTTCAAGTGACAATAAAAACTGATGTATTTGCATTTGGAGTGGTTCTGTCTGAATTGATAACAGGAAAAAGAGCATTATTTCGTGACAACCAAGA-------AGC---CAATAATATGAAATCACTTATTGCAGTTATTAACAAAATATTTCAAGATG---ATGACCCGGTGAGTGCTTTAG------AAGCAACTGTAGATGGAAATCTTCTACGTAGTTATCCTCTTGAAGATGTCTACAAGGTGGCAGAATTATCTCATTGGTGTTTGAGTGAAAATCCAGTGGACAGACCTGAAATGAAAGAAATGGTTGTAGCATTATCAAAGATTGTAATGTCCTCAATAGAATGGGAAGCATCACTAGGTGGGGACAGTCAAGTTTTCAGTGGG---GTATTTGATGGAAGATGA----

>LjEPR3_/_LjLYS3_CDS

------------------------------------------------------------------------------ATGTTTTATGATTTCACAACTATGGCT---TCTCTAACTCATCCTCTATGTGTTCTCCTTACTCTAATGGCTGC-------AGCTTCATTTG-----CAAGTGTGTTTTCCCTTG---AAGTTTCATCCAAAA---CAACTTACATGGAACCTTTTAACTGCTCTACAAAG---AT-CAGAACATGC--AATTCCTTGCTCTACCACATAAGCATCG---GTCTCAA------GGTCGAAGAAATAGCCC---------------GCTTTTACTCGGTTAATCTC---TCCCGAATCAAGCCAATAACTCGC---------GGTACCAAGCA---AGATTACCTTGTCTCAGTGCCTTGTACTTGCAGAAACACCAATGG------CCTTAATGGATATTTCTATCATACATCCTACAAG---GTTAAGGTTAATGACAGTTTTGTGGATATTCAGAACCTGTTCTATAGTGGACAAGCTTGGCCTGTGAATG------AAGATTTGGTGGT---TCCAAATGAGACTATGACAATACATATTCCTTGTGGGTGTTCAGA---------------AAGTGGCTCTCAAATTGTCG-TCACATACACAGTTC-AAAGGAATGA-TACACCATTATCAATTGCTCTTTTGCTAAATGCTACGGTTGAAGGCATGGTGAGTGTGAACTCAGTTATGGCTCCGAATCCCACATTCATAGATGTTGGTTGGGTGTTATATGTTCCCAA---GGAGTTGAA------TCCAAT------------------TTCCCATGGAAAAGAAAATAAACACAA---GCTG---GAGAAAATTATTGGCATCTTAGCGGGTG---TGATATTACTTTCAATTATTACCTTGATCATTCTTA------TCGTCAGGAGAAATAGA---TCCTATGAAAC---CTGCAAAGATGATCCA---CGCGCTATCTCAAAAAGATCAATCGGCAAAAG---------------------------AAC---TAGTTCCTTAATGAACCGC-GACT-TTCACAAAGAATACATGGAAGATGCGACATCATTTGACTCAGAAAGACCAGTAATTTATACTCTTGAGGAGATTGAACAAGCTACTAATGACTTCGATGAAACTCGAAGGATTGGAGTCGGTGGATATGGAACAGTGTATTTTGGAGTGTTAGGGGAGAAGGAGGTTGCTATAAAGAAGATGAAATCTAACAAATCCAAAGAATTCTATGCAGAACTCAAGGCCTTGTGTAAGATCCATCACATTAACATTGTGGAGTTATTAGGATATGCCAGCGGAGATGACCACCTTTACTTGGTGTATGAGTATGTGCCCAATGGATCTCTCAGTGAACATCTTCATGATCCCTTACTGAAAGGTCACCAGCCTCTTTCTTGGTGTGCTAGGATTCAAATTGCACTGGATTCAGCAAAAGGTATTGAATACATACATGATTACACAAAAGCACAGTATGTGCACCGCGATATAAAGACTAGTAATATTCTTCTTGATGAGAAGCTCAGAGCAAAGGTAGCAGATTTTGGGCTTGCAAAGCTAGTAGAACGAACCAATGATGAAGAATT---CATAGCAACAAGGCTTGTTGGAACACCAGGCTATCTTCCACCAGAATCTCTAAAGGAGCTTCAAGTGACAGTAAAAACTGATGTATTTGCATTTGGGGTGGTTATGTTAGAGTTGATAACAGGGAAACGTGCACTATTTCGTGACAACCAAGA-------AGC---CAACAATATGAGATCACTTGTTGCAGTTGTTAACCAAATATTTCAAGAAG---ATAACCCTGAGACTGCTTTAG------AAGTTACCGTGGATGGGAATCTACAACGTAGCTATCCTATGGAAGATGTCTACAATATGGCAGAACTATCACACTGGTGCTTGCGCGAAAATCCGGTGGACAGGCCTGAAATGAGTGAGATCGTTGTGAAATTGTCAAAGATTATAATGTCCTCGATAGAGTGGGAAGCATCACTTGGCGGAGACAGCCAAGTCTTCAGCGGG---GTATTTGATGGAAGATGA----

>Lup020722

------------------------------------------------------------------------------------------------------------------------------------------------------------------------------------------------------------------------------------------------------------------------------------------------------------------------------------------------------------------------------------------------ATGCGT---------GGCACCAAACA---AGATTACCTCATAACAGTACCTTGTTCTTGCAAAAAAACCATAGA------CCTTAGTGGATATTTCTATGATGCAACCTACAAG---GTGAACCCAAATGATAGTTTTTTGGACATAAAAACCATGATTTATAGTGGCCAAGCATGGCCTAATAATA------GTGTATTGGT------TTCAAATGAGAATTTAGTAATACATATTCCATGTGGGTGTTCCGA---------------AAGTGATTCTCAAATTGTTG-TAACATATACAGTCC-AGTGGAGTGA-TACACCAACGTCTATTACTAATCTGCTAAATGCAACATTTGATGGTTTGGTCAGTATGAACCAAGTTCTGAATCAAAACCCATCATTCATAGATATTGGTTGGGTGATATTTGTTCCTAT---GGAATTGAATGGGCTTCCACA------------------GCCCAATGGAAAAGGAAAAAAACACAT---TTGG---GTAATAATCATTGGGATTTTAGCAGGTG---TGGCATCACTTTCAATTATTACAATGATCTTTCTCA------TTTTCGGGAGGAAAAAA---CCCTATGAAAC---TAGCGAAGACGGTCCA---AACATTGTCTCCAAGAGATCAATTGCCAATAG---------------------------AAC---TATTTCCTTGAAGAACCAA----T-TTCATAGAGAATCTTTGGAAGG------ACAATTTGAAACAGAAAGACCAGTAGTTTATACTCTTGAGGAGATCGAAGATGCTACAAATAACTTCGATGAAACTCGAAAGATTGGAGTTGGTGGATATGGGGAGGTATATTTTGGAATGTTAGAA---AAGGAGGTTGCTGTGAAGAAGATGAAGTCTACTAAATCTAAAGAATTCTATGCAGAACTCAAGGCCTTATGTAATATCCATCATATTAACATTGTAGAGTTGTTGGGATATGCTAGTGGAGATGACCACCTTTATTTGGTGTATGAGTATGTTCCAAATGGATCTCTCAGTGAACATCTTCAAGATCCATTGCTGAAAGGTCACCAGCATCTTTCTTGGTGTGCTAGGACTCAGATTGCATTAGATGCAGCAAAAGGTCTTGAATACATACATGATTATACGAAATCACGATATGTTCACCGTGATGTAAAGACAAGCAATATTCTTCTTGATGAGAAGCTTAGAGCAAAGGTGGCAGATTTTGGACTTGCAAAGCTAGTAGAACGGACAAATGATGAAGAATT---CATAGCAACAAGGCTTGTTGGAACACCAGGCTATCTTCCACCAGAATCTCTGAAGGAGCTTCAGGTAACCGTAAAAACCGATGTATTTGCATTTGGAGTAGTTCTGTCAGAGTTGATAACAGGGAAACGTGCACTGTTTCGTGACAGCAAAGA-------AAC---CACCAAAATGAAATCGCTTATTGCAGTAGTTAATGGTATATTCCTAGACG---ATGAGCCAGAGGCTGCTTTAC------AAGAAGTCATTGATGTGAATCTTCAAAGTAACTATCCTGTAGAAGATATCTACAAGATGGCAGAGCTAGCTCACTGGTGCATGCGCGAAGATCCAGTGGACAGGCCTGAAATGAGGGAGGTTGTTGGCACATTATCACAGATTGTGATGTCCTCTATAGAGTGGGAAGCATCACTAGGTGGAGACAGCCAGGTTTTTAGTGGA---GTATTTAATGGTAGATGA----

>aradu.V14167.gnm1.ann1.Aradu.R7N81

------------------------------------------------------------------------------------ATGATGAAGAACACCATGGCTT---CTCTTACTCATCAACTCCCTCTTCTGCTTACCTTTGTGGCATCATATGCATTTGTTCTTACCACAACTAGTGTGTTCTCATCGA---AAGTTTCAATGAAAG---AAACAATTTTGGAACCTTTCAAGTGCTCCACAAAC---AT-AAAAACATGC--AATGCCTCACTCTACCACATAACCTACG---GGGATAA------TGATGATGACCAAACCATACAACATTACATATCTTCCAACTACACAAACTCC---TCCCAAATCAGACCAATCACACGT---------GGCACAAGACA---AGACCACCTCATAACAGTGCCTTGTTCTTGCAGAAACAACAACAA------CCTCAACGGTTATTACTATGACACAACCTACACT---GTGAAGCCAAATGATAATTTCTATGACATTAATAACTTTGTTTACAGTGGTCAAGCGATGCTTATAAATG------GTGTTTTGTA------CCCGGGTCAGAATCTGAGTATTCATATTCCCTGTGGGTGTTCAGA---------------AATTGAGTCACAAATTGTTG-TGACTTACACGGTTC-AAAGAAATGA-TACCTCATCTGGAATAGCTAAGCTTTTGAATTCTACGGTTGCTGAGATGGAGAGTGTGAATCATCTTTTGGATCAGAACCCATCATACATAGATGTGGGTTGGGTTTTGTTTGTTCCTAG---CCATTTTAATGGAATTCCACT------------------GCCTCCAACAAAAGAAAAGAAACCAAA---GTGG---CAGATAATCACTGGGGTCTTAGTGAGTG---TGACATTACTGTCAATGATTGCCGTCATCTTTCTCA------TTCTCAGGAGAAATAGG---GATAACGCAGCATCCGCCACAAATAATCCA---AAAAGTGTCACTAAAAGATCG---------------------------------------------ATTTCCAAAAAGAACTCT-GACA-TTCATAAAGAATACATGCAAGATGCTACATCATTTGAGTCAGAAAGACCAGTAATTTACTCAATTGAGGAGATAGAAGATGCCACAAATAACTTCGATGAAAACAGAAGAATTGGGGTTGGTGGATATGGCAGTGTATATTTTGGAATGTTAGGCCACAAGGAGGTTGCTGTGAAGAAGATGAAGTCCAATAAATCCAAGGAGTTCTTCGCAGAACTCAAGGCCTTATGTAAGATCCATCACATAAACATTGTGGAGTTGTTAGGGTATGCAAGTGGAGATGACCACCTTTATTTGGTGTACGAGTATGTTGCAAATGGATCTCTGAGTGATCATCTTCATGATCCTTTACTTAAAGGTCATCAGGCTCTATCTTGGTGTGCTAGGACTCAGATTGCATTGGATGCAGCAAAAGGAATTGAATACATACATGATTACACAAAAGCACAATATGTTCACCGTGATATAAAGACTACTAACATTCTTCTTGATCAGAAACTCAGAGCCAAGGTAGCAGATTTTGGCCTCGCAAAACTGGTGGAACGGACCAATGATGAAGAATT---CATTGCAACAAGGCTTGTTGGAACACCAGGCTACCTTCCACCCGAATCTGTGAAAGAATTACAAGTAACCATAAAAACGGATGTGTTTGCATTTGGAGTAGTTCTATCAGAGCTGATAACAGGAAGACGTGCGCTATTTCGTGACAGCCAAAA-------ACC---CAATCAAATGGAATCTCTTATTTCACTTGTTAAGAAAATATTCCAAGATA---ATGATCCCGTCACTGCTTTAG------AAAATGTCACAGATATGAATCTTCAACATAACTATCCTATAGACGCTATCTACAAGATGGGAGAAATAGCAGAGTGGTGCATGAGGGAGGAACCAATGGAGAGGCCTGAAATGAAGGAGATAGTTGGCGCGTTGTCAAAGATAGTTATGACATCCATCGAGTGGGAAGCATCGCTCGGAGGAGATAGCCAAGTTTTCAGCGGC---GTATTTATTGGAAGATGA----

>arahy.Tifrunner.gnm2.ann1.6V1TUE.1

------------------------------------------------------------------------------------ATGATGAAGAACACCATGGCTT---CTCTTACTCATCAACTCCCTCTTCTGCTTACCTTTGTGGCATCATATGCATTTGTTCTTACCACAACTAGTGTGTTCTCATCGA---AAGTTTCAATGAAAG---AAACAATTTTGGAACCTTTCAAGTGCTCCACAAAC---AT-AAAAACATGC--AATGCCTCACTCTACCACATAACCTACG---GGGATAA------TGATGATGACCAAACCATACAACATTACATATCTTCCAACTACACAAACTCC---TCCCAAATCAGACCAATCACACGT---------GGCACAAGACA---AGACCACCTCATAACAGTGCCTTGTTCTTGCAGAAACAACAACAA------CCTCACCGGTTATTACTATGACACAACCTACACT---GTGAAGCCAAATGATAATTTCTATGACATTAATAACTTTGTTTACAGTGGTCAAGCGATGCTTATAAATG------GTGTTTTGTA------CCCGGGTCAGAATCTGAGTATTCATATTCCTTGTGGGTGTTCAGA---------------AATTGAGTCACAAATTGTTG-TGACTTACACGGTTC-AAAGAAATGA-TACCTCATCTGGAATAGCTAAGCTTTTGAATTCTACGGTTGCTGAGATGGAGAGTGTGAATCATCTTTTGGATCAGAACCCATCATACATAGATGTGGGTTGGGTTTTGTTTGTTCCTAG---CCATTTTAATGGAATTCCACT------------------GCCTCCAACAAAAGAAAAGAAACCAAA---GTGG---CAGATAATCACTGGGGTCTTAGTGAGTG---TGACATTACTGTCAATGATTGCGGTCATCTTTCTCA------TTCTCAGGAGAAATAGG---GATAACGCAGCATCCGCCACAAATAATCCA---AAAAGTGTCACTAAAAGATCG---------------------------------------------ATTTCCAAAAAGAACTCT-GACA-TTCATAAAGAATACATGCAAGATGCTACATCATTTGAGTCAGAAAGACCAGTAATTTACTCAATTGAGGAGATAGAAGATGCCACAAATAACTTCGATGAAAACAGAAGAATTGGGGTTGGTGGATATGGCAGTGTATATTTTGGAATGTTAGGCCACAAGGAGGTTGCTGTGAAGAAGATGAAGTCCAATAAATCCAAGGAGTTCTTCGCAGAACTCAAGGCCTTATGTAAGATCCATCACATAAACATTGTGGAGTTGTTAGGGTATGCAAGTGGAGATGACCACCTTTATTTGGTGTATGAGTATGTTGCAAATGGATCTCTGAGTGATCATCTTCATGATCCTTTACTTAAAGGTCATCAGGCTCTATCTTGGTGTGCTAGGACTCAGATTGCATTGGATGCAGCAAAAGGAATTGAATACATACATGATTACACAAAAGCACAATATGTTCACCGTGATATAAAGACTACTAACATTCTTCTTGATCAGAAACTCAGAGCCAAGGTAGCAGATTTTGGCCTCGCAAAACTGGTGGAACGGACCAATGATGAAGAATT---CATTGCAACAAGGCTTGTTGGAACACCAGGCTACCTTCCACCCGAATCTGTGAAAGAATTACAAGTAACCATAAAAACGGATGTGTTTGCATTTGGAGTAGTTCTATCAGAGCTGATAACAGGAAAACGTGCGCTATTTCGTGACAGCCAAAA-------ACC---CAATCAAATGGAATCTCTTATTTCACTTGTTAAGAAAATATTCCAAGATA---ATGATCCCGTCACTGCTTTAG------AAAATGTTACAGATATGAATCTTCAACATAACTATCCTATAGACGCTATCTACAAGATGGGAGAAATAGCAGAGTGGTGCATGAGGGAGGAACCAATGGAGAGGCCTGAAATGAAGGAGATAGTTGGCGCGTTGTCAAAGATAGTTATGACATCCATCGAGTGGGAAGCATCGCTCGGAGGAGATAGCCAAGTTTTCAGCGGC---GTATTTATTGGAAGATGA----

>arahy.Tifrunner.gnm2.ann1.7J5ZWH.1

------------------------------------------------------------------------------------ATGATGAAGAACACCATGGCTT---CTTTTACTCATCAACTCCCTCTTCTGCTTACCTTTGTGGCATCATTTGCATTTGTTCTTACCACAACTAGTGTGTTCTCATCGA---AAGTTTCAATGAAAG---AAACAATTTTGGAACCTTTCAAGTGCTCCACAAAC---AT-AAAAACATGC--AATGCCTCACTCTACCACATAACCTACG---GTGATAA------TGATGATGACCAAACCATACAACATTACATATCTTCCAATTACACAAACTCC---TCCCAAATCAGACCAATCACACGT---------GGCACAAGACA---AGACCACCTCATAACAGTGCCTTGTTCATGCAGAAACAACAACAA------CCTCACCGGTTATTACTATGACACAACCTACACT---GTGAAGCCAAATGATAATTTCTATGACATTAATAACTTTGTTTACAGTGGTCAAGCGATGCTTATAAATG------GTGTTTTGTA------CCCGGGTCAGAAACTGAGTATTCATATTCCTTGTGGGTGTTCAGA---------------AATTGAGTCACAAATTGTTG-TGACTTACACGGTTC-AAAGAAATGA-TACCTCATCTGAAATAGCTAAGCTTTTGAATTCTACGGTTGCTGAGATGGAGAGTGTGAATCATCTTTTGGATCAGAACCCATCATACATAGATGTGGGTTGGGTTTTGTTTGTTCCTAG---CCATTTTAATGGAATTCCACT------------------GCCTCCAACAAAAGAAAAGAAACCAAA---GTGG---CAGATAATCATTGGGGTCTTAGTGAGTG---TGACATTACTTTCAGTGATTGCCGTCATCTTTCTCA------TTCTCAGGAGAAATAGG---GATACCGCAGCATCCGCCACAAATAATCCA---AAAAGTGTCACTAAAAGATCA---------------------------------------------ATTTCCAAAAAGAACTCT-GACA-TTCATAAAGAATACATGCAAGATGCTACATCATTTGAGTCAGAAAGACCAGTAATTTACTCAATTGAGGAGATAGAAGATGCCACAAATAACTTCGATGAAAACAGAAGAATTGGGGTTGGTGGATATGGCAGTGTATATTTTGGAATGTTAGGCCACAAGGAGGTTGCTGTGAAGAAGATGAAGTCCAATAAATCCAAGGAGTTCTTCGCAGAACTCAAGGCCTTATGTAAGATCCATCACATAAACATTGTGGAGTTGTTAGGGTATGCAAGTGGAGATGACCACCTTTATTTGGTGTATGAGTATGTTGCAAATGGATCTCTGAGTGATCATCTTCATGATCCTTTACTTAAAGGACATCAGGCTCTATCTTGGTGTGCTAGGACTCAGATTGCATTGGATGCAGCAAAAGGAATTGAATACATACATGATTACACAAAAGCACAATATGTTCACCGTGATATAAAGACTACTAACATTCTTCTTGATCAGAAACTCAGAGCCAAGGTAGCAGATTTTGGCCTTGCAAAACTGGTGGAACGGACAAATGATGAAGAATT---CATTGCAACAAGGCTTGTTGGAACACCAGGCTACCTTCCACCTGAATCTGTGAAAGAATTACAAGTGACCATAAAAACGGATGTGTTTGCATTTGGAGTAGTTCTATCAGAGCTGATAACAGGAAAACGTGCGCTATTTCGTGACAGCCAAAA-------ACC---CAATCAAATGGAATCTCTTATTTCACTTGTTAAGAAAATATTCCAAGATA---ATGATCCCGTCACTGCTTTAG------AAAATGTTACAGATATGAATCTTCAACATAACTATCCTATAGACGGTATCTACAAGATGGGAGAAATAGCAGAGTGGTGCATGAGGGAGGAACCAATGGAGAGGCCTGAAATGAAGGAGATAGTTGGCGCGTTGTCACAGATAGTTATGACATCCATCGAGTGGGAAGCATCGCTCGGAGGAGATAGCCAAGTTTTCAGCGGC---GTATTTATTGGAAGATAA----

>Araip.IX7QU

------------------------------------------------------------------------------------ATGATGAAGAACACCATGGCTT---CTTTTACTCATCAACTCCCTCTTCTGCTTACCTTTGTGGCATCATTTGCATTTGTTCTTACCACAACTAGTGTGTTCTCATCGA---AAGTTTCAATGAAAG---AAACAATTTTGGAACCTTTCAAGTGCTCCACAAAC---AT-AAAAACATGC--AATGCCTCACTCTACCACATAACCTACG---GTGATAA------TGATGATGACCAAACCATACAACATTACATATCTTCCAATTACACAAACTCC---TCCCAAATCAGACCAATCACACGT---------GGCACAAGACA---AGACCACCTCATAACAGTGCCTTGTTCATGCAGAAACAACAACAA------CCTCACCGGTTATTACTATGACACAACCTACACT---GTGAAGCCAAATGATAATTTCTATGACATTAATAACTTTGTTTACAGTGGTCAAGCGATGCTTATAAATG------GTGTTTTGTA------CCCGGGTCAGAAACTGAGTATTCATATTCCTTGTGGGTGTTCAGA---------------AATTGAGTCACAAATTGTTG-TGACTTACACGGTTC-AAAGAAATGA-TACCTCATCTGAAATAGCTAAGCTTTTGAATTCTACGGTTGCTGAGATGGAGAGTGTGAATCATCTTTTGGATCAGAACCCATCATACATAGATGTGGGTTGGGTTTTGTTTGTTCCTAG---CCATTTTAATGGAATTCCACT------------------GCCTCCAACAAAAGAAAAGAAACCAAA---GTGG---CAGATAATCATTGGGGTCTTAGTGAGTG---TGACATTACTTTCAGTGATTGCCGTCATCTTTCTCA------TTCTCAGGAGAAATAGG---GATACCGCAGCATCCGCCACAAATAATCCA---AAAAGTGTCACTAAAAGATCA---------------------------------------------ATTTCCAAAAAGAACTCT-GACA-TTCATAAAGAATACATGCAAGATGCTACATCATTTGAGTCAGAAAGACCAGTAATTTACTCAATTGAGGAGATAGAAGATGCCACAAATAACTTCGATGAAAACAGAAGAATTGGGGTTGGTGGATATGGCAGTGTATATTTTGGAATGTTAGGCCACAAGGAGGTTGCTGTGAAGAAGATGAAGTCCAATAAATCCAAGGAGTTCTTCGCAGAACTCAAGGCCTTATGTAAGATCCATCACATAAACATTGTGGAGTTGTTAGGGTATGCAAGTGGAGATGACCACCTTTATTTGGTGTATGAGTATGTTGCAAATGGATCTCTGAGTGATCATCTTCATGATCCTTTACTTAAAGGACATCAGGCTCTATCTTGGTGTGCTAGGACTCAGATTGCATTGGATGCAGCAAAAGGAATTGAATACATACATGATTACACAAAAGCACAATATGTTCACCGTGATATAAAGACTACTAACATTCTTCTTGATCAGAAACTCAGAGCCAAGGTAGCAGATTTTGGCCTTGCAAAACTGGTGGAACGGACAAATGATGAAGAATT---CATTGCAACAAGGCTTGTTGGAACACCAGGCTACCTTCCACCTGAATCTGTGAAAGAATTACAAGTGACCATAAAAACGGATGTGTTTGCATTTGGAGTAGTTCTATCAGAGCTGATAACAGGAAAACGTGCGCTATTTCGTGACAGCCAAAA-------ACC---CAATCAAATGGAATCTCTTATTTCACTTGTTAAGAAAATATTCCAAGATA---ATGATCCCGTCACTGCTTTAG------AAAATGTTACAGATATGAATCTTCAACATAACTATCCTATAGACGGTATCTACAAGATGGGAGAAATAGCAGAGTGGTGCATGAGGGAGGAACCAATGGAGAGGCCTGAAATGAAGGAGATAGTTGGCGCGTTGTCACAGATAGTTATGACATCCATCGAGTGGGAAGCATCGCTCGGAGGAGATAGCCAAGTTTTCAGCGGC---GTATTTATTGGAAGATAA----

>Mimpud_scaffold35782_cov175_CDS

---------------------------------------------------------------------------------------------------ATGGCTTCCTTTAGTCTTCTTCCTTCTCTTATTCATATCTCTTTGCTGGCAAC-------TCTACTT--------GTCACAGTGTCTTCCTTTC---AAACTTCCATCAAAG---TATCTTATATGGCCCCTTTTAAATGCTCTGAAAGT---AT-CAACACATGT--AATGCCTTGCTTTACCACATAAACCATG---GTCTTGC------CAAAGATGAAATAGCCT---------------CCTTTTACTCTGTCAATTCT---TCCCAAATCAAAACAATAAGTCAT---------GGTACCAACCA---AGACTACCTCATAACTGTTCCTTGTTCTTGTAAAACTGTCACTAAT------GTTAGGGGATATTTCCATGACACAACATACATA---GTGGAGAAAAATGATACATTTAATAATATTTCAGAATTCTATTACAGTGGACAAGCCTTTTCTCCTACTG------ATCATTTGAC------CATAGGTGAAAACTTAACAATACATCTTCCCTGTGGATGTATTCA---------------AAGTGATTCTCAAATCGTCG-TGACATATACTGTCC-AGCAGAATGA-TATAGTGACAGGAATTGCAAATCTGTTATCAGCCACACCTACTGACATTCAGAACATGAATGAAGTTTTAGCTAATGGGGCTTCATTCATAGATGTGGGTTGGGTGTTATATGTTCCTAT---GTATCTCAATGGGATTCCATC------------------CTCAAAAGGAAGTGGAAAGGGACTCAA------GCATTCTATTATCCTTGGTGTCTTAGTGGGTT---TACCATCACTTTCTTTTATCACAATACTCATTCTCA------TTCTTTGGAGAAAAAAA---GGCACTAAAAC---CAACAAAGAAGATACA---AAAGTCATCTCAAAAAGATCAACTTCAAAAGCATTATCTAAAAGGTCTATTTCCAATAGAACCGATCTTTCCTTGAGGAACCAT-CAAC-TTCATATAGAAACTATAGAAGAAATAACATCAATTGACTCCGAAAGACCAGTAACATTTTATCTTGAGGAGATTAAAGAGGCCATAAACAAG------GAAAATAAAATAATAGGGAAAGGAGGATATGGGACTGTGTATTTTGGAATTTTAGAAGATAAGGAAGTTGCCATTAAAGAGATGAGGTCTAATAAATCCAAAGAGTTCTATGCAGAACTAAAGGTCTTGTGCAGGATCCATCATATCAACATTGTGGAGCTATTGGGATACGCCAGTGGAGAAGATAATCTCTATTTGGTGTATGAATATGTTTCAAAAGGATCTCTCAGTGATCATCTTCACAATCCATTTCTAAAAGGTCATGAACCTCTTTCATGGACTGCTAGAGCACAGATTGCTTTGGATGCTGCAAAAGGTCTTGAATACATACACGATTACACAAAGGCACGGCATGTGCACCGAGATATCAAGACTAGCAATATTCTTCTTGACAATAAACTCAGAGCCAAGATTGCAGATTTTGGGCTAGTAAAGCTGGTAGAACGAACAAGTGATGATGAATT---CATAGCAACAAGGCTTGTTGGAACACCAGGCTACCTTCCCCCAGAATCTGTGAAGGAGCTTCAGGTGACCTCGAAAACAGATGTTTTCGCATTCGGAGTAGTTCTTGCAGAGCTAGTAACAGGAAGACCTGCTTTGTTTCGTGACAGCCAAGA-------AAC---CCCCAGAATGAGATCACTTATTTCTGTTATTAATGAAGTATTTGAAGCTA---ATGATCCAGAGAGTTCTTTAG------AGGATGTCATAGACAAAAACCTTCGGGATTGCTATCCTGTGGAGGAGGTCTTCAAGATGATAGAGATAGCAGAAAGATGCTTGCGTGAAGAACCAGTAGAGAGGCCAGAAATGAGGGAGATTGTGGCCACATTGTCACATGTTTTAATGTCCTCCACGGAGTGGGAAGCATCATTAGGTGGAAACAGTGAAGTCTTTAGTGGC---ATATTTACTGGAAGATGA----

>Proalb_NW_021636151.1_CDS

---------------------------------------------------------------------------------------------------ATGGCTTCCTTTAATCTCCTTCCTCCTCTAACTCATCTCCCTCTGCTGGCCAC-------TTTATTT--------GTTACAGTTTTTTCCTTTC---AGACTTCCATCAAAG---CATCTTATGTAGTCCATTTTAAATGCTCTGCGAAA---AT-CAACACATGT--AATGCCTTACTCTACCACATAAACCAAG---GTCTTGA------CATAGAAGACATAGCCT---------------CTCTTTACTCTGTTAATTCA---TCCCAAATCCAACCAATAACAAGG---------GGGGCCAATCA---AGACTATCTCATAACTGTCCCTTGTTCTTGTAAAGCCGTCACAAAC------CTTAGGGGATATTTCTTTGACACAACATACACA---GTGCAGCAAGATGAGACATATATGAATATCTCAAACTTCTATTACAGCGGACAAGCCTGGTCTCCTACTG------ATAATCTGAC------CACAGGTGAAAACTTAACAATACATCTTCCCTGTGGATGTACAGA---------------AAGTGATTCTCAAATCATTG-TGACATATACGGTCC-AGCAGAATGA-TACAACAGCAGAAATCGCCAATTTGCTATCAGCTACGCTTACTGGCATTCAGAACATGAATGGAGTTTTAGCTAATAGGCCTTCATTCATAGATGTGGGTTGGGTGTTATATGTTCCTAA---GGAGCTCAATGGGATTCCATC------------------TTCAAAAGGAAGTGGAAAGAAACGAAA------GCGTTCCATTATCATTGGTGTCTTGGCGGGTG---TGGCATTACTTTCTGCTATCACAATAACCATTCTCA------TTCGATGGAGAAAAAGA---GCCACTGAAAC---CAGCAATGAAGATGCA---AAAGCTGTATCGAAAAGATCAACTTCAAAAGCATTTTCTAAACGATCGATTTCCAATAGAAC---TCTTTCCTTAAAGAACCAT-CAGT-TTCATATAGAAACTATAGAAGAAGTAACATCAATTGACTCAGAAAGACCAGTAACATTTTATCTTGAGGAGATTGAGGAGGCCATAAACAAA------GAAGGTAAAATAATAGGAAAGGGCGGATATGGGACTGTGTATTTTGGAATTTTAGGAGATAAGGAGGTTGCTATAAAAGAGATGAGGTCTAGTAAATCCAAAGAGTTCTATGCAGAACTGAAGGTCTTATGCAGGATCCATCATATCAACATTGTGGAGCTATTGGGATATGCCAGTGGAGAAGATAACCTCTATTTAGTGTATGAGTATGTTTCAAAAGGATCTCTCAGTGACCATCTTCACGATCCATTTCTAAAAGGTCATGAACCTCTTTCATGGACTGTTAGAGCACAGATTGCTCTGGATGCTGCAAAAGGTCTTGAGTACATACATGATTACACAAAAGCACGGCATGTGCACCGAGATATCAAGACTAGCAATATTCTTCTCGACAATAAACTCAGAGCCAAGATTGCAGATTTTGGACTAGTAAAGCTGGTAGAACGAACAAACGATGATGATTT---TATAGCAACAAGGCTTGTTGGAACACCAGGCTACCTTCCCCCGGAATCTGTGAAGGAGCTTCAGCTGACCTCAAAAACCGACGTTTTTGCATTTGGAGTAGTTCTAGCAGAGCTAGTAACAGGGAAACCTGCTCTATTTCGTGACAGCCAAGA-------AAC---CTGCAGAATGAGATCGCTCATTTCTGTCATCAATAAAGTATTTGAAGCTA---ATGACCCAGAGAGTGCTTTAG------AGGATATCATAGACAAAAACCTTCGAGATACCTATCCTGTGGAGGATGTCTTCAAGATGATAGAAGTAGCTGAAAGATGCTTGCGTGAGGAACCAACAGAGAGGCCAGAAATGAGGGAGATTGTGTTGATTTTGTCACAGATTTTGATGTCCTCCACAGAATGGGAAGCATCATTAGGTGGAAACAGTGAAGTCTTTAGCGGC---GTATTTACTGGAAGATGA----

>Chafa3673S21651

---------------------------------------------------------------------------------------------------ATGGCTTCCCTTAATCTCCTTCCTTCTCTAGCTCTTCTCTCTCTTCTGTCTAC-------TTCATTT--------CTCACACTTTCTCTCTCAC---AAACTTCAAATAATAATTCATCTTACATATCCCCTTTTAAATGCTCTGAAAAA---AT-CAACACATGC--AATGCCTCACTTTACCACATAAACCAAA---ATCCAAATCCATCCATAGAAAATTTGGCCT---------------CTCTTTACTCTGTTGATCCA---TCAAAAATCAAACCAATAATGCAT---------GGAACCAATCA---AGATTACCTCATAACAGTGCCTTGTTCTTGCAGAAACACCAGTGAT------CTTAGTGGATATTTCTATGACACAAACTACACA---GTGAAGCAACATGAGACATTCTCATATATTTCAAACTTCTATTATAGTGGACAAGCATGGTCTGGTAGTG------ACAATATTAC------TTCAGGTCAAAACTTGACTATACATCTTCTTTGTGGATGCTCAGA---------------AAGTGGTTCTCAGATCGTCG-TGACCTATACCGTCC-AGAAAGGCGA-TACAACAACAGCAATTGCTGATCTTCTATCTGCTACACTCACTGGGATGCAGCAAATGAATGAACATTTGGCTGAAAACCCGGATTTCTTAGTTGCAGGTTGGGTGTTATATGTTCCTAA---GGAGTTGAATGGGATTCCATC------------------TTCAAAA---AGTGGACACAAAGCCAA------ACATGCTATTATCATTGGAGTCTTAGCAGGAA---TAACAACACTTTCTATAATGACAATGGTTATTCTCA------TTCTCTGGAAAAGAAAAAGTGGCAATCAAAC---CAGCAAAGTATTTCTG---AACTCTGCATCTAGAAGATCAGTTGCTAGAGATTTATCGAAAAGATC------------AAC---TCTTAGCTTGAAGAACCGT-CACC-TTCATAGTGAAAATATAGAAGATGCAACACCATTTGACTCAGAAAGACCAGTGATATACTATCTTGAGGAGATCGAAAAGGCTACGAATAATTTTGATGAAAGCCGAATAATCGGAAAAGGCGGATATGGGAGTGTGTATTTTGGAGTATTAGGGGACAAGGAGGTTGCTGTGAAAGTGATGAGGTCGAATAAATCCAAAGAGTTCTATGCAGAACTCAAGGTCTTATGTAGGATTCATCATATCAACATTGTGGAGCTATTAGGATATGCCAGTGGAGAAGACCATCTTTATTTGGTTTATGAGTATGTTTCAAATGGATCTCTCAGTGAACATCTTAATGATCCATTTAGTAAAGATCATCAACCTCTTTCATGGACTTCTAGAGTACAAATTGCTCTGGATGCTGCAAAAGGTCTAGAATACATACATTATTACACAAAAGAGCGATACGTGCACCGAGATATAAAGACTAGCAATATTCTTCTGGATGGCAAGCTCAGAGCAAAGGTAGCAGATTTTGGACTGGCAAAACTGTTAGTACAAGCAAATGATGAAGATTT---TGTAGCAACAAGGCTTGTTGGAACACCAGGCTACCTTCCACCAGAATCTGTGAAGGAGCTTCAGGTGAATGCGAAAACCGATGTGTTTGCATTCGGAGTGGTTCTAGCAGAACTGATAACCGGAAAACGCGCGTTATTTCGGGACAGCAAAGA-------TGC---CAATAGAATGAGGTCACTTATTTCAATTATTAATATGATATTCCAAGAAC---ATGATCCTGCAAGTGCTTTGG------AGGATGTGATAGATAAAAATCTTCAAGATAACTATCCCATGGAAGATGTCTTCAAGATGGCAGAAATAGCCGAAAAGTGCATGCGTGAAGATCCAATAGAGAGGCCAGGAATGAGGGAGATAGTGGTGATGTTGTCACAGATTGTGATGTCTTCAATAGAGTGGGAAGCATCACTCGGTGGAAATAGTCAAGTCTTTAGTGGT---TTATTTACCGGAAGATAA----

>Mimpud_scaffold5325_cov212_CDS_v2

---------------------------------------------------------------------------------------------------ATGTCTT---CTCTCACTCTTCTTTCTCTTTTACTTCTCCTTTTTCTAGCAAA-----------------------------CCCCTTCAATAT---GATCATCAAGACCCAAG-CATTTGACAAAGAACCCATGAATTGTTCTTCATCAACTTT-CTTGCAATGC--AATGCCTCCTTGTATCACATCACCAATAATGGTCTCACCGA---CAAAAAAGACATTGCTT---------------CACGTTACTCCGTGGACGTG---TCCCATTTGAAACCTATAAAGCGTCCTAACAATAATATTGGACACGAAGATTACCTTGTAACTGTCCCTTGTACTTGCCTGAATACCAAAGAC------CTTAGTGGTTACTTCTATAAGACAACCTACACA---GTGGAGCTTGGTGACACAGTGCACAATATTTCAGATTTTGTTTACAGTGGGCAAGCTTGGATCCTTGGGAATCATAGCCTTTTGAA------CATAAGTGAGATTTTAACAATATATCTTCTCTGTGGGTGTCCTACAAGTGGTAGTAGTGGTAACGGGCCTCAGATTTTTG-TGACGTATACGGTGC-AGAAGGATGA-ATCTCTCTTCACAATTGCTAGTCTTCTGAATTCAACCGTGGTTGACATGAAGTATTATAATTGGATTTTAAAGTCAAACCCATCTTTTTTAGGTGTAGGGTGGGTTCTATTTATTCCTAG---TTACTTAAATGGTATTCCATTCCTCAATGATAGTCATGGTTCTACTAGTAGAGGGAACCAGTTTAA------------ATTGGTCGCTGGCATATTGGCTGGTT---TCATTTTGCTTTTGTTGCCCATAGCAATTCTACTCA------TTAATAGAAGAAGAAAA----------AGA-----AGCAAAGAAG---------------CACCTAAAAAATCAGTTTCCCATAG----------------------------------AAGTCCTTCAAATTC------CC-TTCATAAAAAGATGGTAAAAGATGAAACAGCATTTGAATCAGAAAAACCAGGAATATTTAGTCTTGAGGAAATTGAAGAGGCTACTGATTACTTTGACGAAACCAGAAAAATTGGAGTTGGTGGATTTGGAAGTGTGTATTTTGGAGTCATGGGTGAAAAGGAGGTTGCAATTAAAAAGATGAGGTCTAATAAATCCAAAGAGTTCTATGCAGAACTGAAGATCTTATGTAAGGTCCATCATATCAACATTGTAGAGCTTTTGGGATATGCCAGTGGAGAAGACCACCTCTTTGTGGTGTATGAATATGTTGCAAATGGATCTCTCAGTGACCATCTTCACGATCCAGTACTAAAAGGTCATCAGCCTCTTTCTTGGAATGCCAGAGTTCAGATTGCTCTAGATGCTGGAAAAGGTCTTGAATATATACATGATTACACCAAAATGCGATATGTTCATCGTGATGTAAAGACTAGCAATATCCTTCTTGACGAGAAGCTCAGAGCAAAGGTGGCAGATTTTGGACTTGCAAAACTGATAGAAGGAACAAATGATGAAGATACTAACATAGCAACAAGGCTTGTTGGAACACCTGGCTACCTTCCACCAGAATCTGTGAAGGAGTTTAAGACGACTCCAAAATCTGATGTGTTTGCATTTGGAGTGGTTCTATCAGAGCTGGTAACAGGGAAACGTGCCTTGTTTCGTGACAGCCAAGA-------GTG---CAACAAAATGAAATCCCTCATTACACTTGTCAATGATATATTCGAAGACA---ATGACCCTGAGGCTGCTTTGG------AAAATGTCATCGATCGGAATCTCCAAGGTTGCTACCCTCCCGAGGATGTATTCAAGATGATAGAGTTAGCTGAGAGATGTTTAAGGGAAGATCCATCGGAGAGGCCTGATATGAAGCAGGTGGTTGAAGTACTGACATATCTCGTGATGTCGTCCATGGATTGGGAAGCTTCACTTAGAGAGAAAAGTCAGGTCTTCAATGTCCTCGTATGTAAAGCAAGATGA----

>Datgl1333S01924

---------------------------------------------------------------------------------------------------ATGAAAGCTTCCTTCAATTCACTGTCTTGTCTCTTCTTTCTTGTAGTTTTTGCA------TATTTTT------------CTGATATCTCATCTA---GCATTTCTAATGAAA---------CCATGTCTCCAATGACATGTTCTTCACAA---AA-CAAGCCATGT--AATGCCTCTTTGTACCACATCAACAATG---GCCTCAA------TGAATCTGAAATTGCTT---------------CATCTTACTCCGTAAAGGTT---TCCCAAATTAGAACCATAATGGTA---------GGTAGCAGACA---AGACTACCTAATATCAGTCCCTTGTTCTTGTCAAGCTGTTAATGGC------ATTTCTGCGTACTTTTACAGCACTTTCTACAAC---GTTCAAATTGGTGACAGTTCTGACAGCATTTCTGAGGGTAGATATAATGGGCAAGCTTTTATGGTTGGCA------AAAATCAAACGCTTTTTCCGGGGAAAAATT---CTGTTCAATTGTTGTGTGGGTGTGTGGA---------------AAGTGAGTCAGAAATTGTGG-TCACATACACAGTTC-AGAGACATGA-TACTTTGTCACAAATTGCTACTCTGTTATCTGCTCATGAAAGTAATATAGAGAGCTTAAACAGAAATTTGATTAAGAATCCACAGTTCATAGACGAAGGTTGGGTGTTGTTTGTTCCCAT---GGAAAAGAATGGGATTCTGCAGC---------------CAACATCAACAAAAGAAAAGAGTCGTCA---ATGG---ATTATAATAGTGGGAATATTATCAGCCG---TTTCTATAATTTCAATATGTTCATTGATAATCATCA------TTCTGAGGAGAAAAAGA--TTGCAACAAAGC----AATGAGGAAGATCCAAAATTAGCCGTGTCCAAGAGCCTGAGTGGCAACAG---------------------------AAA---CTTTTCCTTGCAGCATCTG-TCCC-TGAACAAGGAAAACATGCAAGATGCACCGGGTTTCAAAACAGAAAAACCAGTAGTTTTTACCATTGAACAGATTGAAGAGGCCACAAATAATTTTGATGAGACACAGAAAATTGGAGAAGGTGGATATGGAACTGTTTATTTTGGAGTAATAGGTGACCAGGAAATTGCAGTAAAAAAGATGAAATCTAATAAATCCAAAGAGTTCTTGATAGAGCTAAGGGTTCTATGTAAGATTCATCACATTAATGTGGTGGAGCTGTTGGGATATGCCAGTGGAGATGATCACCTCTACTTAATCTATGAATTTGTTCAAAATGGATCCCTGAGTGACCATCTCCATGATCCATTACTGAAAGGTTACCAGCCTCTCTCTTGGACAGCAAGAACACAAATTGCTTTGGATGCTGCAAAGGGTATCGAATACATTCATGACCACACGAAAGCACAGTATGTGCACCGTGATATAAAGACGACTAACATCCTACTTGATGAAAGCCTCAGAGCAAAGGTAGCTGATTTTGGTTTAGCAAAGTTAGTTGGAAGAACCAACGAAGATGACCT---CATTGCTACACGACTAGTCGGAACACCGGGTTACCTTCCTCCAGAATCTGTGAAGGAGCTCCAAGTTACCCCGAAAACAGATGTTTTTGCATTTGGTGTCGTCTTAGCGGAGCTTATTACAGGGACACGAGCACTTGTTCGGGACAAACAGGA-------ACC---CAACAAGACAAAGTCTCTCATAAAACTTGTCAATAAGATATTTGAAGATG---AAGATCCCGAGACAGCTTTAG------AAGCTTCCGTAGATGGAAATTTACAAGGCAACTACCCTTTTGAGGATGCGTATAAGATGGCAGAAATAGCTGCATGGTGTTTGAGTGAAGATGCAGTAAACAGACCAGAGATGAGGGAGATTGTTACAACATTATCTCAGATTGTGCAAGCTGCTATAGAGTGGGAAGCTTCATTAGGTGGGAATAGCCAGGTTTTCAGCGGG---CTTTTCAGTGGAAGATGA----

>LjLYS5

---------------------------------------------------------------------------ATGATTCCTTTAGCAAAACCCCAATGGAAGTTTCTCC------TCCTTTTTCTGGTTTTCATTCATTTCAAGAGAACCAGTTCTTACCCTATGGAACCCATGAACTGTACGGACACAAGCCGTGTCTGCACTTCTTTCATGGCCTTTAAGCCTCAACCGAAGCAGACACTGGCGGAGATACAGAGCATGTTTGATGTGTTGCCTGGTGACATCACTGTTGAAGG---CAATGGCTGGGACTACATGTTCATCAGGAAGA---------ATTGTTCTTGTGCTGCTGGTA--TCAAGAAATATGTGTCTAACACCACCT----TCACTGTGAAATCTAATGAAGGGTGGGTG--TATGATTTGGTAATGGATGCCTATGATGGGCTTG--TGATCCTTCCTAATACCACAA---GGAGGGCAAG---GAATGGTGCTGTTATCTCTCTGAGGTTGTTCTGTGGCTG--TTCCAGTGGACT-TTGGAACTATCTGATG-----AGTTATGTGAT------GACAGATGGGGATAGTGTCGAATCTCTGGCGAGCAGATTCGGGGTTAGTA---------TGGGCAGCATTGAGTCTGTAAATGGCATTGGTGATCCTGATAATGTGACTGTGGGCTCACTTTATTACATACCTATGAATTCGGTTCCTGGTGATCCTTATCCCCTGAAGAATGCTTCTCCACCAGCTCCTGTTCCTACCCCATCTGTTGATAATTTTTCAGGTGATCAAGTCGATCATAAGGCTCATGTACCATATGGATGGATTATTGGGGGTCTAGGAGTTGGTCTTTTTCTGATAATATTA---AGCGTAATGCTCTGTGTTTGTATGAGATCA-TCAAGTTGTTTTGGTGAAGCC---AGA-AGTCATGAAAAAGATGCTGACGGAAAGATCTCTCATAAATTCCATATTCTTCGGAATCCAAGTTTCTTTTGTGGTTCCGGAAGGTACATCTGTGGCAAACATGTAGGCCAGAAG------CAAAAAGATGGTGAATCCAGCAATCACACGATTACCATTCCCAAAGCTTCAACTTTGGGGCCTGACATATTTGACATGGATAAGCCTGTAGTTTTTACATACGATGAGATTTTCCCCTCAACTGATGGATTCTCTGATTCAAATCTACTTGGGCATGGAACATATGGATCTGTTTACTATTGCCTCCTTCGTGACCAGGAAGTTGCTATTAAAAGAATGACTGCTACAAAAACAAAAGAATTTATGGCAGAGATAAAAGTTCTGTGCAAGGTTCATCATGCTAATCTGGTAGAATTGATTGGCTATGCAGCAAGTCATGATGAGTTTTTCCTAGTTTATGAATATGCTCAAAAGGGTTCACTCAGAAGCCATTTGCATGATCCTCAAAATAAGGGGCATTCCCCTCTTTCTTGGATCATGAGGGTGCAGATCGCACTTGACGCTGCAAGGGGCCTTGAATACATTCATGAACACACAAAAACTCATTATGTCCACCGTGATATCAAGACAAGCAACATTTTACTGGATGCTTCCTTTAGAGCAAAGATTTCAGATTTTGGATTAGCAAAACTTGTTGGGAAAACAAATGAAGGAGAAGT---ATCAACTACCAAAGTTGTTGGTACATATGGATATCTTGCTCCAGAATACTTGAGTAATGGCCTTGCAACTACCAAAAGTGATGTATATGCATTTGGTGTTGTCCTTTTTGAGATTATATCAGGAAAGGAGGCCATCATTCG-AACAGAAGGCACAGTGACAAAAAATCCGGAAAGACGTTCACTGGCTTCAGTAATGTTGGCAGCTCTTAGGAACTCACCTGATTCCATGAGCATGTCAGGCGTGAGAGATTATATTGATCCAAATATGATGAATCTGTATCCCCATGATTGTGTATTTAAGATGGCCATGCTGGCAAAGCAATGCGTGGACGATGATCCAATCTTACGACCTGATATGAAGCAGATTGTGATTTCTCTCTCACAGATTCTCCTTTCTACTGTTGAGTGGGAAGCCACTCTAGCTGGGAATAGCCAAGTATTCAGTGGC---CTTGTTCAGGGAAGATAG----

>MtLYK11

---------------------------------------------------------------------------ATGATTCTCGTAGGAAAACCCCATTTGAAGTTGCTTCAATTTGTTCTGTTTCTGTTCTACCTTAATTTTAACAGAAGTAGTTCTAATCAGATGGCTCCAATGAACTGTACGGACACAAGACGAGTTTGCACATCTTTCTTGGCCTATAAGCCTCAACAGAATCAATCTTTGGGAGTGATTCAAAGCATGTTTGATGTGTTACCAAGTGATATCACAGTTGAAGG---AAATGGTTGGGATTATATATTCATAAGGAAGA---------ATTGTTCTTGTGCATCTGGGA--TCAAGAAATACGTGTCTAATACAACTT----TTACTGTGAAAACCAATGAAGGGTTTGTG--GATGATTTGGTTATGGATGCTTATGATGGGCTTA--TTTTGCTTCCAAATACTTCAA---GGAAGGCAAG---GAATGGTGCTGTTATATCATTGAGGTTGTTTTGTGGCTG--TTCTAGTGGATT-ATGGAATTATTTGTTG-----AGTTATGTGTT------GAGAGATGGGGATAGTGTTGAATCTTTGGCTAGTAGATTTGGGGTTAGTA---------TGGATAGTATTGAGGGTGTGAATGGCCTTGATGGTCCTGATAATGTTACTGTCGGTTCACTTTATTATATTCCTCTTGATTCTGTTCCTGGTGATCCTTATCCTCTGAAGAATGCTTCTCCACCAGCTTCTGTTCCTACCCCTTCTGTCGATAATATTTCAGGTGATCAGGACAATCATAAATATCATGTACCCTATGGATGGATCATTGGAGGTTTAGGAGTTGGTCTTATTCTGATAATATTA---GGCATAATTCTCTGTGTTTGCCTGAGATCA-TCAAATTGCTTTTCTGATTCT---CGA-AGTCATGAAAAAGATGCTGAGGGAAAGGTCTCTCATAAATTCCAAATTCTTCGGAATCCAAGTTTTTTTTGTGGTTCTGGACGGTACATATGCGGCAAACATGTTGACCAAAAG------CAAACAGACGGCGACTCCAGCACTCACACGATTACTGTTCCCAAAGCTTCAACACTAGGGCCAGACGTATTTGACATGGATAAGCCGGTAGTTTTTGCATATGAAGAGATTTTTTCCTCAACTGAAGGCTTCTCTGATTCAAATCTACTTGGACATGGAACATATGGATCTGTCTATTATTGCCTCCTTCGTGACCAGGAAGTCGCTATTAAAAGAATGACAGCTACAAAAACAAAAGAGTTTACATCAGAGATTAAAGTTCTGTGCAAGGTTCATCATGCTAATCTGGTAGAATTGATTGGCTATGCAGCTAGTCACGATGAGCTTTTCCTAGTTTATGAATATGCTCAGAAGGGTTCACTCAGAAGCCATTTGCATGATCCTCAAAATAAGGGTCATTCACCACTTTCATGGATCATGAGGGTTCAGATTGCACTTGATGCTGCTAGGGGACTTGAATATATACATGAGCACACAAAAGCTCATTATGTCCACCGTGATATCAAGACAAGCAACATTTTACTTGATGCTTCTTTTAAAGCAAAGATTTCTGATTTTGGGTTGGCGAAACTTGTCGGGATAACAAATGAGGGAGATGT---TTCAACTACAAAAGTTGTTGGTACATATGGATATCTTGCTCCGGAATACTTGAGTGACGGCCTTGCAACAACCAAAAGTGATGTCTATGCATTTGGTGTTGTCCTTTTTGAGACTATAACTGGAAAGGAGGCCATTATTCG-AACAGAAGGCATGATGACAAAAAATCCTGAAAGACGATCACTCGCATCGATAATGTTGGCAGTTCTTAGGAACTCACCTGATTCCTTGAGCATGTCAAGCATGAAAGATTACATTGATCCAAATATGATGAATCTATATCCCCATGATTGTGTATTTAAGATGGCTATGCTGGCGAAGCAATGTGTGGACGACGATCCGATCTTACGACCTGATATGAAAACAGTAGTGATTTCCATCTCACAGATTCTTTTGTCTTCTATTGAGTGGGAAGCAACACTAGCTGGGAATAGCCAAGTATTTAGTGGA---CTTGTTCAGGGAAGATAG----

>LjLYS4

---------------------------------------------------------------------------ATGTATCTTACACAGAAACCATGTCTGAAGTTGCTAC---TGCACTTCCCTATCTTTCTGCTTCACTTTTACAGCATCTATTCTTATCCCACA---CCCATGAACTGCACCGACTCAACCCGCGTCTGCACTTCTTTCTTGGCCTTTAAGCCTCAACCAAACCAGACCTTAGCAGTGATAGAGAGCATGTTTGATGTTTTGCCTGGTGACATCACTGTTGAAGG---CAATGGCTGGGGTTACACATTCATCAGAAAAA---------ACTGTTCTTGTGCTGCTGGGA--TTAAAAAATATGTTTCTAACACCACAT----TCACAGTGAAATCCCATGGAGGGTTTGTG--ACTGACATGGTGATGGATGCCTATGATGGGCTTG--TTTTCTTGCCCAACACGACGACACGCTGGGCAAG---AGAAGGTTCTGTGGTGCCTCTGAGCTTGTTCTGTGGCTG--CTCCAGTGGACT-GTGGAATTATTTGGTG-----AGTTATGTGAT------CAGAGATGGGGACAGTGTTGAATCTTTGGCAAGTAGGTTTGGGGTTAGTA---------TGGATAGCATTGAGACAGTGAATGGCATCAGCAATCCTGATAGTGTCATTGTTGGCTCTCTTTACTATATACCTTTGAATTCAGTTCCTGGTGAGCCTTATCACCTGAAGAATGATACTTCTCCGGTTCCTGTTCCTTCACCATCTGTTGATAATTTTTCAGCTGATGATATCAACCGTAAAGCTCACGTACCCTATGGATTGATCATGGGAGGTATAGGGGTTGGTCTTGCACTAATAATTATA---AGCATGATACTGTGTGTGTTCCTGAGATCA-TCAAATTGTTTAGTTGAAGCC---AGA-AATCAAGCAAAGGTTGCTGAGGGTAATATCTCTCATAAGCTCCATA------GGAGTCCAATTTTGTTTTGTGGGCCTGGAAGATTCATATGCTGCAAACCTGTAGAC------------CAAACTGATGGTGAATCCAGCAGTGACCAAATTACTGCTCCTAAACCTTCAACTCTAATGCCAGAAGTTTTTAACATGGATAAGCCAGTAGTTTTTACATATGAAGAGATTTTTTCCTCAACTGATGGTTTCTCAGATTCAAATCTTCTTGGCTACAAAACATATGGTTCTGTTTACTATGGTCTCCTTCGTGACCAGGAAGTTGCCATTAAGAGAATAACAGCTACTAAAACCAAAGAATTTATGTCAGAGATGAAAGTTCTGTGCAAGGTCCATCATGCTAATCTGGTAGAATTCATCGGCTATGCGCCTAGTCATGATGAGGTTTTCCTTGTTTTTGAATATGCTCAGAAAGGTTCACTCAGTAGCCATTTGCATGATCCTCAGAATAAGGGTCATTCATCACTTTCTTGGATCACAAGGGTTCAAATTGCACTTGATGCTGCTAGGGGCCTTGAATACATACATGAGCACACAAAAACTCGTTACGTCCATCAAGATATCAATACAAGCAACATTCTTCTTGATGCTTCCTTCAGAGCCAAGATATCAGATTTTGGGTTAGCAAAACTTGTTAGTGAAACAATTGAGGGAGGAAC---CACAACAACCAAAGGTGTTAGTACATATGGATATCTTGCTCCTGAATACTTGAGCAATCGCATTGCGACGTCCAAAAGTGATGTCTATGCATTTGGTGTTGTTCTTTATGAGATTATTTCAGGGAAGAAAGCCATTATTCA-AACACAAGGTA------CACAAGGTCCTGAAAGACGATCATTGGCATCTATAATGTTGGAAGTTCTTAGGACCGTACCTGATTCATTGAGCACGCCAAGCATTAGAAACCACGTTGATCCTATCATGAAGGATCTGTATTCCCATGATTGTGTATTGCAGATGGCCATGCTGGCAAAGCAATGTGTGGAGGAGGATCCCATTTTACGTCCTGATATGAAGCAAGTTGTGCTTTCCCTGTCCCAGATCCATCTTTCTTCTTTTGAGTGGGAAGCCACTCTAGCTGGGAAAAGCCAGGTTTTCAGTGGG---CTTATTCAGGGAAGATAG----

>PanLYK4

---------------------------------------------------------------------------ATGAATCTGAGATCG--ACCCCATTTGGGATTCTAGCA-TGCATTCTTCTCCTTCTCTTGCTCCAAGCTCGCTCTCTGTTCTCGTACCCCACG---CCCATGAACTGTACGGACACGACTCGGCTCTGTACCTCTTTCTTGGCTTTCAAGCCGAAGGAGAACCAGACCCTGGCCGTGATCCAGAGCATGTTCGACGTTTTGCCCGGCGACGTGACCGTCGAGGGGGCCGGCGGACGGGGATACGTGTTCGTCAGGAAGA---------ACTGCTCGTGCGCGTCGACGA--TCAAGAAGTATGTGACGAATACAACTT----TTACGGTCAAATCAAGTGAAGGGTTGGTA--TACGACATGGTTATGGAGGCCTACGATGGGTTGG--CTTTACTGCCCAATACGACGA---GGCCGGCGAG---GTACGGCGCTGTCGTTTCGTTGACGCTCTTCTGTGGGTG--CTCTAGTGGGCT-GTGGAACTATCTGATG-----AGCTATGTGAT------GGAGGATGGGGATACCATCGAATCGTTGGCGAGTCGGTTCGGTGTTAGTA---------TGGATAGCATCGAGAAAGTTAATAGGATCGACAATCCTAACAATGTCAGTGTGGGTTCTCTGTTTTACATACCTCTAGATTCAGTTCCTGGTGATCCTTATCCTTTAAAGGCTGATGTTCCTCCGGCTCCTGCCCCTGCTCCATCAAGTGACAGTTTTTTAGGAAATCAAGTAATTCATAAAGCTCATGCACCCTATATATGGATTGTCGGGAGTTTGGGGCTTGTTCTCGCTCTTATTGTGATA---GGTATAGTGGTATATGTTTCCTTGAGGTCA-TCAAAGTGCTTTACTGAAGCACAGAGA-GATCATTCGAAAGATTCAGACAGCAAGAATTCTTATAAGTTCCACATTCTTCGAAAGCCAAGTTTCTGTTGTGGTTCAGGAAGATACATCTGCTGCAAGTCTGGAGATTGCAAG------CAAAATAATGGAGAACCTAAGAGCCACCAAATAACTATTCCCAAAGCT------CTTGGGACTGATGTATTTGAAATGGAGAAGCCTGTGGTTTTCACTTATGAGGAAATGTTTTCTTCCACCGATGGCTTCTCAGATTCAAATCTTCTGGGGAATGGCACTTATGGCTCTGTGTACTATGGCCTCCTTCATGACCAGGAAGTTGCTGTTAAAAGAATGACTGCTACAAAAACTAAAGAATTTCTGGCAGAAATGAAAGTTCTATGCAAGGTTCATCATACAAATCTGGTAGAATTGATTGGGTATGCAGCTACTGATGATGAGCTCTTTCTCATATATGAGTATGCACAAAAAGGTTCACTTAGAAATCATTTACACGATCTTCAAACCAAGGGTAATACATCACTTTCCTGGATCATGAGGGTCCAGATTGCACTTGATGCCGCTAGAGGTCTTGAGTACATTCATGAGCACACAAAAGCTCATTATGTCCACCGAGATATTAAAACAAGCAACATCTTACTTGATAGCACCTTCAGGGCAAAGATTTCAGATTTTGGGTTGGCAAAACTTGTAGGAATAACAAGTGAAGGGGAATC---TACAACAACAAAAGTTGTTGGTACATTTGGCTATCTAGCTCCAGAATATTTGAGTGATGGCCGTGCTACAGCAAAAAGTGATGTTTATGCATTTGGAGTCGTTCTATTTGAGATTATATCAGGAAAAGAGGCTACTATTCG-AACAGAAGGTGTGGCCATGAAAAATCCTGAAAGGCGTTCTCTGGTATCCATTATGTTAGGAGCTCTAAAGAACACGTCCGACTCCATGAGTATGTCAAACATGAAAGACTATATTGATCCTAACATGATGGATTTGTATCCGCATGATTGTCTGTTCAAGATGGCCATGCTGGCAAAACAATGTGTGGAGGATGATCCAATGTACCGGCCTGACATGAAACAAATAGTGATTTCTCTGTCACAGATACTCCTCTCCTCTGTGGAGTGGGAAGCAACTCTTGCCGGGACCAGCCAAGTATTCAGTGGC---CTTGTCCAAGGAAGATAG----
